# Synthesis of multivalent carbohydrate mimetics with aminopolyol end groups and their evaluation as L-selectin inhibitors

**DOI:** 10.3762/bjoc.11.72

**Published:** 2015-05-05

**Authors:** Joana Salta, Jens Dernedde, Hans-Ulrich Reissig

**Affiliations:** 1 Freie Universität Berlin, Institut für Chemie und Biochemie, Takustrasse 3, D-14195 Berlin, Germany; 2 Charité Universitätsmedizin Berlin, Institut für Laboratoriumsmedizin, Klinische Chemie und Pathobiochemie, Campus Virchow Klinikum, Augustenburger Platz 1, D-13353 Berlin, Germany

**Keywords:** aminopolyols, carbohydrate mimetics, carboxylic acid amides, inhibition, multivalency, selectins, sulfation

## Abstract

In this article a series of divalent and trivalent carbohydrate mimetics on the basis of an enantiopure aminopyran and of serinol is described. These aminopolyols are connected by amide bonds to carboxylic acid derived spacer units either by Schotten–Baumann acylation or by coupling employing HATU as reagent. The *O*-sulfation employing the SO_3_·DMF complex was optimized. It was crucial to follow this process by 700 MHz ^1^H NMR spectroscopy to ensure full conversion and to use a refined neutralization and purification protocol. Many of the compounds could not be tested as L-selectin inhibitor by SPR due to their insolubility in water, nevertheless, a divalent and a trivalent amide showed surprisingly good activities with IC_50_ values in the low micromolar range.

## Introduction

**Scheme 1:**
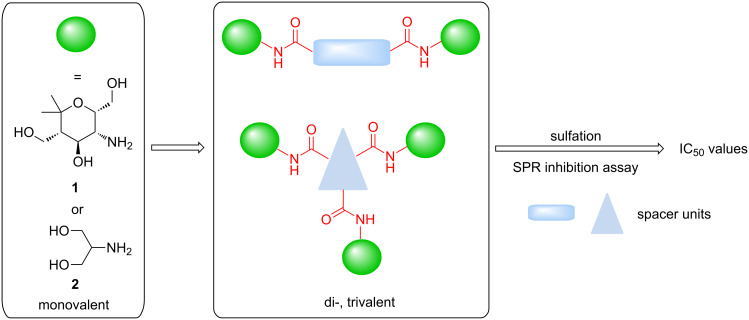
General approach to divalent or trivalent carbohydrate mimetics on the basis of aminopyran **1** or serinol (**2**) and their evaluation as selectin inhibitors.

In a series of publications [1–6] our group reported on the syntheses of carbohydrate mimetics [7–11] that are based on aminopyrans, aminooxepanes or other aminopolyols. These compounds and their conjugates were prepared to be examined as selectin inhibitors. There we have found that sulfated aminopyrans connected by amide bonds to gold nanoparticles are highly potent inhibitors of L- and P-selectin with IC_50_ values in the subnanomolar range [12–13]. These lectins are crucial in the inflammatory process [14–18] and hence compounds inhibiting their activity are of interest as potential therapeutics [19–23]. In a previous report [24] we described the synthesis of divalent carbohydrate mimetics connecting aminopyran **1** or its simplified analog serinol (**2**) (Scheme 1) to different linker units by reductive amination of aldehydes. We now enclose our results on the preparation of related di- and trivalent carbohydrate mimetics in which compounds **1** or **2** are connected to carboxylic acid cores by amide bonds. A series of compounds with spacer units of different length and rigidity were prepared in order to find smaller inhibitors than the above mentioned nanoparticles and also to examine multivalency effects [25–26]. Several of these compounds could successfully be sulfated and tested as L-selectin inhibitors.

## Results and Discussion

**Scheme 2:**
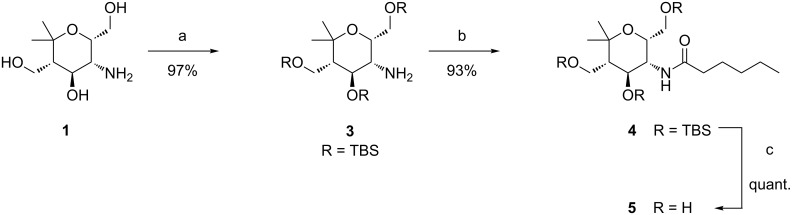
Hydroxy group protection of aminopyran **1** to give compound **3**, synthesis of amide **4** and subsequent deprotection. Conditions: a) TBSOTf, Et_3_N, DMAP, DMF, 5 d, 0 °C to rt; b) hexanoyl chloride, Et_3_N, CH_2_Cl_2_, 18 h, rt; c) HF∙pyridine, 24 h, 0 °C to rt.

Aminopyran **1** was easily available following the previously reported synthetic route [24,27–28], whereas serinol (**2**) is commercially available. As a first approach to construct amide derivatives we envisioned the Schotten–Baumann acylation using acid chlorides. For this purpose a protection of the hydroxy groups of aminopyran **1** with the *tert-*butyldimethylsilyl (TBS) group was chosen. Reaction of **1** with *tert*-butyldimethylsilyl triflate (TBSOTf) and a tertiary amine as base under standard conditions furnished compound **3** (Scheme 2). This transformation required remarkably long reaction times when applied to compound **1** and only after 5 days a yield of 97% could be obtained. As a first model reaction protected aminopyran **3** was treated with commercially available hexanoyl chloride affording the desired amide **4** in excellent yield. After cleavage of the TBS protecting groups, the fully deprotected monovalent aminopyran derivative **5** was isolated in quantitative yield.

**Scheme 3:**
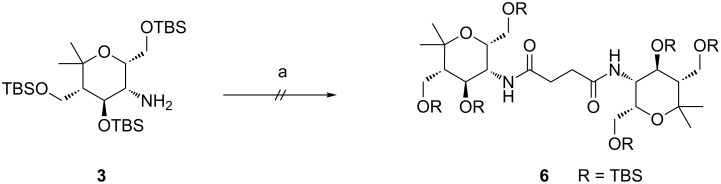
Attempt to synthesize protected divalent compound **6**. Conditions: a) succinic acid dichloride, Et_3_N, CH_2_Cl_2_, 24 h, rt.

After the successful synthesis of the monovalent compound **5**, the same conditions were examined for the synthesis of related divalent systems. When these reaction conditions were applied to protected aminopyran **3** with succinic acid dichloride, the desired divalent product **6** was not formed (Scheme 3). After several attempts changing reaction time and equivalents of protected aminopyran **3** and succinic acid dichloride, neither the desired product **6** nor the corresponding pyrrolidine-2,5-dione resulting from an intramolecular reaction were formed.

It was quite unexpected that we could not achieve this transformation since in the literature similar conditions were found for the synthesis of multivalent acetyl-protected carbohydrates [29]. As possible explanation we assume that the formation of product **6** is sterically too hindered due to the bulkiness of the TBS-protecting groups of **3** and the short distance between the two aminopyran units. For this reason, other dicarboxylic acid derivatives with longer chains and different flexibility were tested and gratifyingly the desired products could be prepared (Table 1). The reaction with the aromatic linker terephthaloyl dichloride (**7**, Table 1, entry 1) afforded the desired protected divalent compound **10** in excellent yield. Using the aliphatic sebacoyl dichloride (**8**) as linker (Table 1, entry 2), the expected product **11** could be isolated in 58% yield. The interesting *trans*-azobenzene derivative **9** [30] was also employed as precursor and the divalent compound **12** was obtained in excellent yield (Table 1, entry 3). This product is particular intriguing since it offers the possibility to generate a light-switchable divalent carbohydrate mimetic. Deprotection using HF∙pyridine complex proved to be an adequate method and all fully deprotected amides **13**–**15** were isolated in excellent yields.

**Table 1:** Synthesis of amides **10**–**12** and subsequent deprotection to give divalent compounds **13**–**15**.

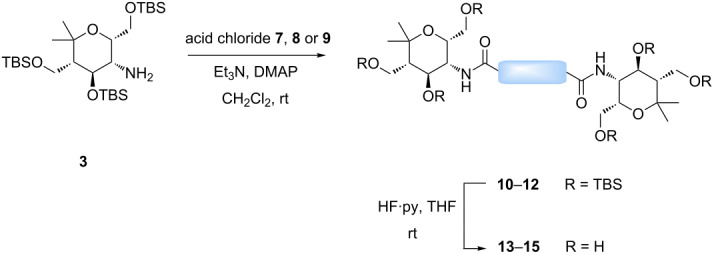							

Entry	Acid dichloride	Time 1^a^ [h]	Product	Yield [%]	Time 2^b^ [h]	Product	Yield [%]

1	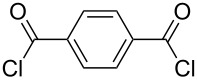 **7**	20	**10**	quant.	22	**13**	quant.
2	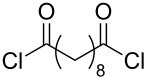 **8**	20	**11**	58	24	**14**	80
3	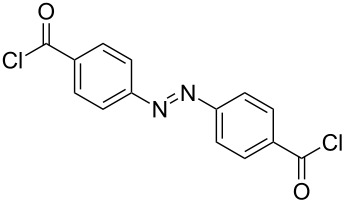 **9**	24	**12**	quant.	24	**15**	quant.

^a^First step; ^b^second step.

As previously mentioned, an additional goal of this study was the investigation of multivalent compounds starting from the simple aminopolyol **2**. Analogously to aminopyran **1**, the hydroxy groups of **2** were first protected with TBS groups under standard conditions to furnish compound **16**. To be able to compare aminopyran **1** with aminopolyol **2** two divalent amides were synthesized from compound **16** using the same carboxylic acid dichlorides **7** and **8** as linkers affording the desired compounds **18** and **19** in excellent yields (Table 2, entries 1 and 2). Moreover, another divalent amide **20** with a longer spacer unit was synthesized using adipic acid dichloride (**17**) as precursor (Table 2, entry 3). Although TBS deprotection with the HF∙pyridine complex proved to be a fairly efficient method (see Table 1), other options were searched in order to find milder conditions, cheaper reagents and a simpler work-up protocol for the very hydrophilic products. Acid-promoted solvolysis in the absence of water [31] was considered as good alternative that should have the advantage of generating side products that can easily be removed in vacuo, making further purification unnecessary. First attempts with acetyl chloride (0.6 equivalents) as source of dry hydrochloric acid and methanol as protic solvent gave only poor conversions, probably due to the low solubility of the starting material **18** in this alcohol. On the other hand, excellent results could be achieved with 2-propanol as solvent. Under these conditions the fully deprotected divalent amides **21**–**23** were isolated in an operationally very simple manner and in excellent yields (Table 2).

**Table 2:** Synthesis of amides **18**–**20** from protected serinol **16** and subsequent deprotection to give divalent compounds **21**–**23**.

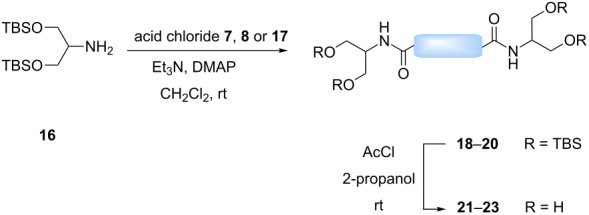							

Entry	Acid dichloride	Time 1^a^ [h]	Product	Yield [%]	Time 2^b^ [h]	Product	Yield [%]

1	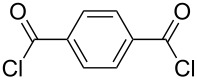 **7**	17	**18**	83	1.5	**21**	90
2	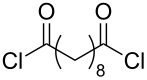 **8**	18	**19**	75	2.5	**22**	quant.
3	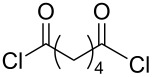 **17**	17	**20**	62	1.5	**23**	97

^a^First step; ^b^second step.

**Scheme 4:**
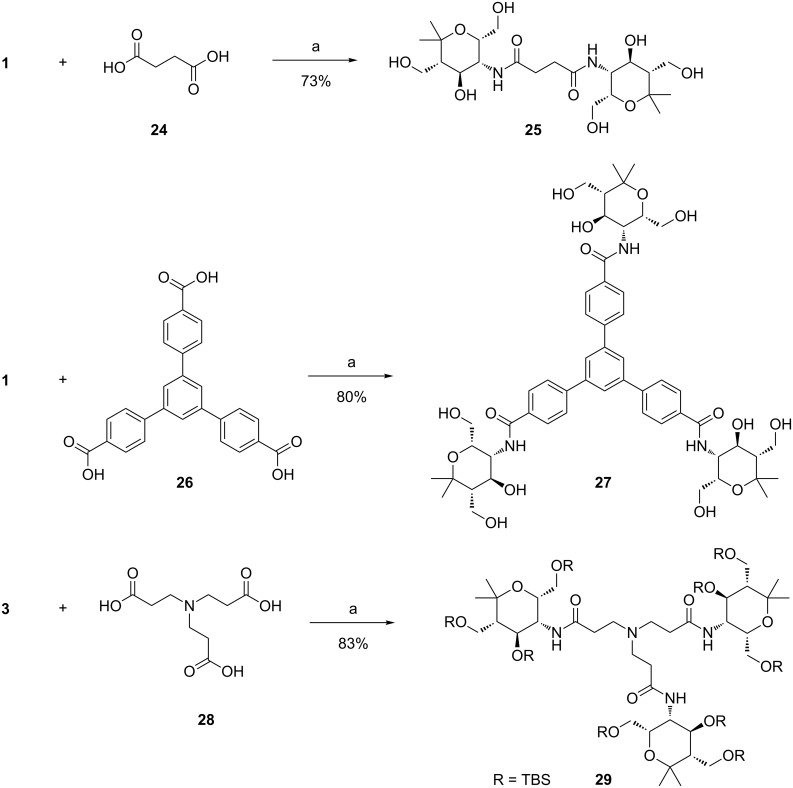
HATU-mediated synthesis of divalent amide **25** and trivalent amides **27** and **29**. Conditions: a) HATU, Et_3_N, DMF, 24 h, rt.

In order to directly obtain the unprotected multivalent carbohydrate mimetics we looked for alternative methods not requiring the protection of the hydroxy groups of **1** or **2**. The most common method in modern synthetic chemistry to generate amide bonds is the use of coupling reagents that first activate the carboxylic acid which subsequently reacts with an amine, also in the presence of unprotected hydroxy groups. From the many known coupling reagents [32–33] we selected HATU (1-[bis(dimethylamino)methylene]-1*H*-1,2,3-triazolo[4,5-*b*]pyridinium-3-oxide hexafluorophosphate), a coupling reagent frequently used in peptide synthesis [34]. We applied standard conditions for the preparation of one divalent and two trivalent amides (Scheme 4). To our pleasure divalent compound **25**, from reaction of unprotected aminopyran **1** and succinic acid (**24**), was isolated in good yield. This successful transformation is evidence that the TBS protected aminopyran **3** is probably sterically too hindered to react with succinic acid dichloride (see above, Scheme 3). Using this procedure, trivalent carbohydrate mimetics **27** and **29** were synthesized in very good yields. Once again it was noticed that the reaction proceeds efficiently even without a large excess of the corresponding aminopyran **1**. For the synthesis of each of the trivalent compounds **27** and **29**, respectively, only 1.3 equivalents of amine per carboxylic acid unit were used. With the aromatic tricarboxylic acid **26** as rigid linker unit, the polarity of the final product is only moderate and the reaction and purification proceeded perfectly. Starting from the aminopyran **1** the desired trivalent compound **27** was received in very good yield. On the other hand, unprotected compound **1** and tricarboxylic acid **28** did not furnish the expected product, most probably due to the high polarity of the coupling product which is then lost during the attempted purification by column chromatography. To overcome these difficulties, TBS-protected aminopyran **3** was used and coupled with **28** efficiently affording the protected trivalent compound **29**.

**Scheme 5:**
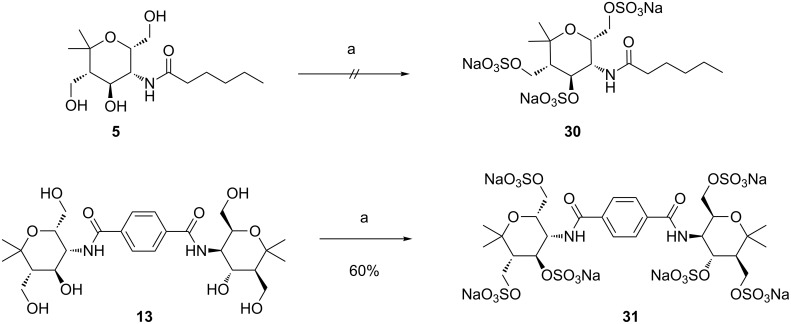
Polysulfations of amides **5** and **13**. Conditions: a) 1) SO_3_∙DMF, DMF-*d*_7_, 1 d, rt; 2) 1 M NaOH, 0 °C; 3) dialysis, H_2_O.

For our planned examination of the multivalent compounds as selectin inhibitors, the *O*-sulfated derivatives were also required. Since the introduction of sulfate groups drastically changes the physical and chemical properties of the molecules, isolation of pure fully *O*-sulfated compounds continues to be a great challenge. The polysulfation of the presented carbohydrate mimetics proved to be the most demanding step of the synthesis requiring many attempts and optimizations to find a suitable and reasonably reproducible procedure. Since other sulfation methods such as SO_3_∙pyridine [35] provided unsatisfactory results an excess of SO_3_∙DMF [13,36] was used as sulfating agent and the resulting sulfuric acid monoesters (in a mixture with an excess of the sulfating reagent) were directly converted into the corresponding sodium salts using a 1 M solution of sodium hydroxide. Subsequent purification by dialysis against water should afford the desired pure polysulfated compounds. We performed the sulfation reactions in deuterated DMF as solvent in order to allow the direct reaction control by ^1^H NMR spectroscopy. When by ^1^H NMR control a mixture of products was observed, additional equivalents of the sulfating agent were added and stirring was continued for another day; this procedure was repeated until full conversion of the compound was observed. Unfortunately, with this protocol the *O*-sulfation and purification of the monovalent model compound **5** did not lead to a homogenous product (Scheme 5). In this case, we tried to follow the reaction progress by ^1^H NMR spectroscopy at 400 MHz which is apparently not sufficiently sensitive. Hence product **31** was contaminated by other compounds. Gratifyingly, the polysulfation of divalent amide **13** afforded the hexasulfated product **31** in 60% yield (full conversion already after one day as observed by ^1^H NMR spectroscopy at 700 MHz).

**Scheme 6:**
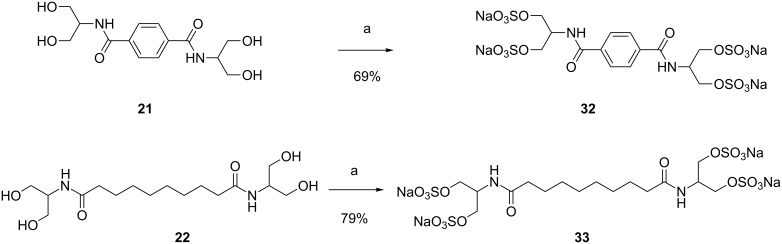
Polysulfation of divalent amides **21** and **22** leading to tetrasulfated amides **32** and **33**. Conditions: a) 1) SO_3_∙DMF, DMF-*d*_7_, 5 d (for **32**), 1 d (for **33**), rt; 2) 1 M NaOH, 0 °C; 3) dialysis, H_2_O.

A polysulfation reaction was also performed with amides derived from serinol (Scheme 6). The sulfation of compound **21** was carried out using 3 equivalents of sulfating agent per hydroxy group for five days. The desired polysulfated compound **32** could be isolated with a good yield. When the sulfation reaction was performed using diamide **22**, the reaction was much faster and after one day a homogeneous product was shown by 1H NMR spectroscopy. In this case no additional equivalents of the sulfating agent were added to the mixture and after dialysis the desired product **33** was isolated in very good yield.

**Scheme 7:**
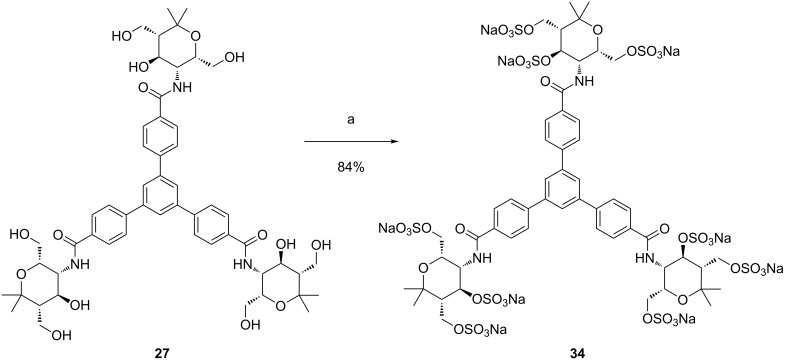
Conversion of trivalent compound **27** into nonasulfated carbohydrate mimetic **34**. Conditions: a) 1) SO_3_∙DMF, DMF-*d*_7_, 3 d, rt; 2) 0.5 M NaOH, 0 °C, DOWEX^®^ Na^+^; 3) dialysis, H_2_O.

The examples depicted in Scheme 5 and Scheme 6 were selected from quite a number of experiments. Often these transformations were not well reproducible due to purification problems. Although the reason for this irreproducibility was not clear, it was noted that during neutralization even addition of small amounts of 1 M sodium hydroxide solution to the reaction mixture did not allow accurate pH control. The resulting highly basic conditions could lead to decomposition or regeneration of the hydroxy groups leading to inhomogeneous mixtures. A better pH control could be achieved using 0.5 M sodium hydroxide solution and hence the pH could be stopped close to neutrality. Additionally, the obtained mixture was filtrated through an ion exchange DOWEX^®^ Na^+^ (50WX8-200) column to assure that all sulfuric acid monoesters as well as the excess of the sulfating agent were converted into the corresponding sodium salts. After this filtration a dialysis of the mixture generally afforded pure products. The modified procedure was applied to the *O*-sulfation of amide **27** and the reaction was complete after 3 days. Sodium hydroxide solution (0.5 M) was added until pH 9 was reached and the mixture was filtrated through a DOWEX^®^ Na^+^ column. After purification, the desired sodium salt **34** was successfully obtained in excellent yield (Scheme 7).

**Figure 1:**
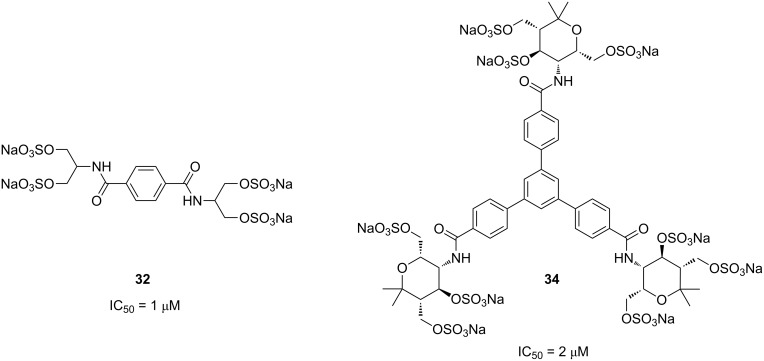
Structures of *O*-sulfated divalent amide **32** and trivalent amide **34** and their respective IC_50_ values for L-selectin as determined by SPR.

For the evaluation of the synthesized carbohydrate mimetics as potential selectin inhibitors, surface plasmon resonance (SPR) spectroscopy [37] was applied. Following the same competitive binding assay previously established for the test of such compounds [38–39], only a few of the presented unsulfated or sulfated compounds could be screened as potential inhibitors. The unsulfated amides **14**, **21** and **22** were not soluble in water and therefore not applicable for testing. The soluble divalent compound **23** (Table 2) did not show any inhibition even at the tested maximum concentration of 1 mM. These negative results are similar to those with the related unsulfated amine derivatives previously reported [24]. We expected that all polysulfated amides are water soluble at concentrations suitable for the SPR assay, but disappointingly amides **31** and **33** (Scheme 5 and Scheme 6) were not sufficiently soluble and therefore no tests could be performed with these compounds. At least divalent amide **32** and trivalent amide **34** showed inhibitory activity as L-selectin ligands in the 1 μmolar range (Figure 1). In a qualitative test compound **34** also inhibited P-selectin, a result to be confirmed in quantitative studies. Quite surprisingly, the flexible divalent serinol derivative **32** showed a good inhibitory potential with an IC_50_ value of 1 μM. The rigid trivalent compound **34** has a slightly inferior activity with an IC_50_ value of 2 μM, but is still a fairly good inhibitor.

When comparing the two *O*-sulfated amides **32** and **34** a multivalency effect is not evident and a sound structure–activity discussion is not feasible as the two compounds have different end groups and different flexibilities. A series of related compounds is required to have a better understanding of structure–property relationships and the influence of multivalency. Unfortunately, only a few of our prepared compounds were sufficiently soluble in water to be suitable for the SPR test. However, a series of other multivalent conjugates was synthesized by using click chemistry with the azide derived from aminopyran **1** and results will be published in due time [40].

## Conclusion

In this article we disclose the preparation of divalent and trivalent carbohydrate mimetics with end groups derived from aminopyran **1** and serinol (**2**). The units were connected by amide bonds that were either formed by Schotten–Baumann reaction using the corresponding acid chlorides or by a coupling of the amines to carboxylic acids using HATU as reagent. The subsequent *O*-sulfation of the obtained compounds with SO_3_∙DMF was optimized with the help of ^1^H NMR spectroscopic control (700 MHz). A crucial detail is also the neutralization step which works reliable only with 0.5 M sodium hydroxide solution. By these methods a few oligovalent *O*-sulfated carbohydrate mimetics could be prepared and tested as L-selectin inhibitors by SPR. The divalent amide **32** and trivalent amide **34** showed surprisingly good activities with IC_50_ values in the micromolar range. Further studies are required to reveal a multivalency effect and to understand structure–property relationships of compounds of this type.

## Supporting Information

File 1 General information, experimental procedures and analytical data as well as copies of NMR spectra of all compounds.
