# Supplementary material for: Synthesis of multivalent carbohydrate mimetics with aminopolyol end groups and their evaluation as L-selectin inhibitors
Source: Beilstein J Org Chem. 2015 May 5;11:638–46. doi: 10.3762/bjoc.11.72 (PMC4464473; doi:10.3762/bjoc.11.72)

Supporting Information File

for

Synthesis of multivalent carbohydrate mimetics with aminopolyol end groups and their evaluation as L-selectin inhibitors

Joana Salta[1], Jens Dernedde[2] and Hans-Ulrich Reissig*[1]

Address: [1]Freie Universität Berlin, Institut für Chemie und Biochemie, Takustrasse 3, D-14195 Berlin, Germany and [2]Charité Universitätsmedizin Berlin, Institut für Laboratoriumsmedizin, Klinische Chemie und Pathobiochemie, Campus Virchow Klinikum, Augustenburger Platz 1, D-13353 Berlin, Germany

Email: Hans-Ulrich Reissig - hreissig@chemie.fu-berlin.de

*Corresponding author

General information, experimental procedures and analytical data as well as copies of NMR spectra of all compounds

Experimental procedures

Table of contents:

- General information page S2–S3
- General procedures and analytical data page S4–S29
- Copies of ^{1}H and ^{13}C NMR spectra and SPR plots page S30–S52

General information

Reactions were generally performed under inert atmosphere (argon) in flame-dried flasks. Solvents and reagents were added by syringe. Solvents were dried using standard procedures and were purified with a MB SPS-800-dry solvent system. Triethylamine was distilled from CaH_2 and stored over KOH under argon atmosphere. Commercially available reagents were used as received without further purification unless otherwise stated. Products were purified by flash chromatography on silica gel (230–400 mesh, Merck or MACHERY-NAGEL) or by ion exchange resin (DOWEX® 50WX8-200 Sigma-Aldrich). DOWEX® Na^+ was freshly prepared by washing DOWEX® with a saturated solution of NaCl. Unless otherwise stated, yields refer to analytically pure samples. TLC-analyses were performed on silica gel coated aluminium plates purchased from Merck. Products were detected by UV-activity and by using staining reagents (cerium/molybdenum reagent, $KMnO_4$ and ninhydrine). NMR spectra were recorded on BRUKER (AV 500, AV 700) and JEOL (ECP 500) instruments. Chemical shifts (δ) are listed in parts per million (ppm) and are reported relative to solvent residual signals: $CDCl_3$ (^{1}H: δ = 7.26 ppm, ^{13}C: δ = 77.2 ppm), CD_3OD (^{1}H: δ = 3.31 ppm, ^{13}C: δ = 49.0 ppm), DMSO-d_6 (^{1}H: δ = 2.50 ppm, ^{13}C: δ = 116.6 ppm), DMF-d_7 (^{1}H: δ = 2.75 ppm, ^{13}C: δ = 29.8 ppm) or D_2O (^{1}H: δ = 4.79 ppm). Integrals are in accordance with assignments; coupling constants (*J*) are given in Hz. All ^{13}C NMR spectra are proton decoupled. Multiplicity is indicated as follows: s (singlet), bs (broad singlet), d (doublet), bd (broad doublet), t (triplet), q (quartet), quint. (quintet), dd (doublet of doublet), dt (doublet of triplet), td (triplet of doublet), m (multiplet), m_c (centered multiplet). For detailed peak assignments 2D spectra were measured (COSY and HMQC). IR spectra were measured with a Jasco spectrometer (FT/IR-4100 with DLATGS Detector). HRMS analyses were performed with Agilent 6210 (ESI–TOF, 10 µL/min, 1.0 bar, 4 kV) and Varian/Agilent Ionspec QFT-7 (ESI–

FTICR, 4 µL/min, 1.0 bar, 4 kV) instruments. Elemental analyses were carried out with instruments from PerkinElmer (CHN-Analyzer 2400) and from Elementar (Vario, Vario EL, Vario EL III). Melting points were measured with a Reichert apparatus (Thermovar) and are uncorrected.

General procedures and analytical data

Amide bond by Schotten–Baumann reaction (GP-1): Under argon atmosphere, the amine (1.0 equiv) was dissolved in CH_2Cl_2 (8 mL/mmol) and the solution was cooled to 0 °C. After addition of Et_3N (2.0 equiv) and the corresponding acid chloride (1.2 equiv) the reaction mixture was stirred from 0 °C to rt during the indicated time. H_2O was added to the mixture and the aqueous phase was extracted with CH_2Cl_2 (3×). The combined organic phases were dried with Na_2SO_4 and the solvents removed in vacuo. The crude product was purified by flash column chromatography.

Amide bond by coupling (GP-2): Under argon atmosphere, the amine (1.0 equiv), the corresponding carboxylic acid (1.0 equiv) and HATU (1.0 equiv) were dissolved in DMF (8 mL/mmol). After addition of Et_3N (4.5 equiv), the reaction mixture was stirred at rt during the indicated time. After removing the solvents in vacuo, the crude product was purified by flash column chromatography.

TBS deprotection with HF·pyridine (GP-3): To a stirred solution of starting material (1 equiv) in THF (9 mL/mmol) at 0 °C, HF·pyridine (ca. 65–70% HF, 8 equiv) was added under argon atmosphere. After warming up to rt, the reaction mixture was stirred during the indicated time. H_2O was added to the mixture and the aqueous layer was extracted with CH_2Cl_2 (3×). The combined organic phases were dried with

Na_2SO_4 and the solvents were removed in vacuo. The crude product was purified by flash column chromatography.

TBS deprotection by solvolysis (GP-4): To a stirred solution of starting material (1.0 equiv) in 2-propanol (3 mL/mmol), AcCl (0.6 equiv) was added at 0 °C. The reaction mixture was stirred at rt for the indicated time. All volatiles were removed in vacuo affording the desired product without further purification.

Polysulfation (GP-5): The polyol (1.0 equiv) was dissolved in DMF-d_7 (0.6–1.0 mL). The solution was cooled to 0 °C and SO_3·DMF (3.0 equiv per OH) was added. The reaction mixture was stirred at rt for the indicated time. The reaction conversion was followed by ^{1}H NMR spectroscopy (700 MHz). When indicated, additional SO_3·DMF (1.0–3.0 equiv for each OH group) was added and the reaction mixture was stirred at rt for the additional given time until full conversion was observed. The obtained sulfated intermediates were directly converted into the corresponding sodium salts according to Method A or Method B.

Method A: The reaction mixture was cooled to 0 °C and an aq. 1 M solution of NaOH was added dropwise until pH 10–12 was reached. The solvents were removed in vacuo and the crude product was purified by dialysis in H_2O.

Method B: The reaction solution was cooled to 0 °C and an aq. 0.5 M solution of NaOH was added dropwise until pH 7–9 was reached. The reaction mixture was filtrated through an ion exchange DOWEX® Na^+ column. The solvents were removed in vacuo and the crude product was purified by dialysis in H_2O.

The final products were filtrated through a syringe filter (diam. 25 mm; pore size 0.2 µm; PTFE membrane) when indicated.

Surface plasmon resonance (SPR) assay: Experiments were performed on a BIACORE X instrument (GE Healthcare, Freiburg, Germany) at 25 °C using a sensor chip (sensor chip SA, GE Healthcare).

The running buffer during the assay consisted of 20 mM HEPES pH 7.4, with 150 mM NaCl and 1 mM $CaCl_2$.

Assay protocol: Selectins (L-, P- or E-) Fc chimeras (R&D Systems GmbH, Wiesbaden-Nordenstadt, Germany) were coupled to Protein A gold particles (AuNP) (diam. 15 nm, Biotrend Chemikalien GmbH, Cologne, Germany) and led over a sensor chip surface with two flow cells (Fc1 and Fc2). On Fc2 was immobilized the selectin ligand composed of sulfated tyrosine and tetrasaccharide sialyl-Le^X presented in a multivalent fashion on a polyacrylamide backbone (sTyr/sLe^X-PAA). On Fc1 (reference lane) was immobilized *N*-acetyllactosamine (LacNac-PAA) as background control. The signal from Fc1 was automatically subtracted from Fc2 during each measurement.

To evaluate selectin binding of potential inhibitors, before loading over the sensor chip, each sample was incubated with inhibitor (protein A gold particles coated with the respective selectin) for 18 min at rt at the desired final inhibitor concentrations.

The samples (35 µL) were injected over the reference lane and over the sTyr/SLe^x-PAA lane at a flow rate of 20 µL/min. Each cycle consisted of aprox. 1 min waiting period for monitoring baseline stability, a 105 s period of association phase and 180 s

dissociation phase. Regeneration of the surface was done by injecting 4 M $MgCl_2$ at a flow rate of 100 µL/min for 60 s.

Measurements without inhibitor served as 100% binding references and were performed before and after each assay series to control baseline deviations. Each concentration was measured in duplicates.

Data evaluation: Reference lane data were subtracted from sTyr/sLe^x-PAA lane data. Responses of the sample injections were extracted between report points set at the start of the injection (0 s) and at the end of the dissociation phase (285 s). Each point represents the mean value of 2 measurements.

The mean value of the first and last 100% values were plotted against total number of data points. A linear regression between both points was set and using the resulting formula, 100% values were calculated for each data point. To obtain the relative binding value all mean results were divided by the respective 100% value. The relative binding was plotted against the corresponding inhibitor concentration and the IC_{50} was determined manually.

(2*S*,3*S*,4*S*,5*S*)-4-(*tert*-Butyldimethylsiloxy)-2,5-bis[(*tert*-butyldimethylsiloxy)-methyl]-6,6-dimethyltetrahydro-2*H*-pyran-3-amine (3)

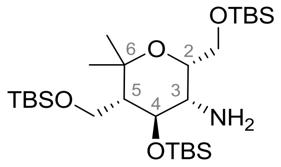

3

To a solution of aminopyran **1** (100 mg, 487 μmol) in dry DMF (5 mL), DMAP (6 mg, 49 μmol) and Et_3N (0.54 mL, 3.90 mmol) were added under argon atmosphere. After cooling to 0 °C, TBSOTf (901 mg, 0.78 mL, 3.41 mmol) was added dropwise and the reaction mixture was stirred for 1 h at 0 °C until it reached rt. After stirring for 5 d at this temperature, a sat. aq. $NaHCO_3$ solution (15 mL) was added to the reaction mixture followed by extraction with EtOAc (3 × 40 mL). The combined organic phases were dried with Na_2SO_4 and the solvent was removed in vacuo. The crude product was purified by flash column chromatography (silica gel, hexanes 100%, hexanes/EtOAc 10:1, 9:1) affording TBS protected aminopyran **3** (268 mg, 97%) as a yellow oil.

$[\alpha]_D^{22}$ +0.72 (c = 1.95, $CHCl_3$); ^1H NMR (500 MHz, $CDCl_3$): δ = 0.040, 0.047, 0.053, 0.055, 0.069, 0.080 (6 s, 3 H each, CH_3), 0.87, 0.88, 0.89 (3 s, 9 H each, *t*Bu), 1.16, 1.44 (2 s, 3 H each, CH_3), 1.66 (dt, $J \approx 3.4$, 7.7 Hz, 1 H, 5-H), 2.75 (m_c, 1 H, 3-H), 3.65 – 3.66 (m, 2 H, 2-CH_2), 3.71, 3.77 (AB part of ABX system, J_{AX} = 7.5 Hz, J_{BX} = 7.9 Hz, J_{AB} = 10.2 Hz, 1 H each, 5-CH_2), 4.01 – 4.04 (m, 2 H, 4-H, 2-H) ppm; ^{13}C NMR (125 MHz, $CDCl_3$): δ = -5.5, -5.3, -5.2, -5.1, -4.7, -4.6 (6 q, $SiCH_3$), 18.1, 18.2, 18.3 [3 s, Si*C*(CH_3)$_3$], 25.9, 26.0, 26.1 [3 q, SiC(*C*H_3)$_3$], 27.4, 27.6 (2 q, CH_3), 49.1 (d, C-5), 52.7 (d, C-3), 62.7 (t, 2-CH_2), 62.9 (t, 5-CH_2), 68.5 (d, C-2), 72.9 (d, C-4), 74.1

(s, C-6) ppm; IR (ATR): $\tilde{\nu}$ = 3390 (N-H), 2955-2860 (C-H), 1255, 1070 (C-O) cm^{-1}; HRMS (ESI-TOF): m/z calcd. for $C_{27}H_{62}NO_4Si_3$ $[M + H]^+$: 548.3981; found: 548.4013.

TBS protected amine 16

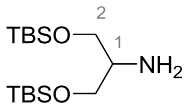

16

2-Amino-1,3-diol (**2**, 8.02 g, 88.0 mmol) and DMAP (50 mg, 0.40 mmol) were dissolved in CH_2Cl_2 (100 mL) under argon. The reaction mixture was stirred at rt and Et_3N (48 mL, 344 mmol) was added. In a second flask *tert*-butyldimethylsilyl chloride (34.0 g, 225 mmol) was dissolved in CH_2Cl_2 (50 mL). The solution was added to the reaction mixture and stirred at rt overnight. H_2O (100 mL) was added to the mixture and the aqueous phase extracted with CH_2Cl_2 (3 × 150 mL). The combined organic phases were dried with Na_2SO_4 and solvents removed in vacuo. The crude product was purified by flash column chromatography (silica gel, hexanes/EtOAc, 2:1) affording **16** (27.0 g, 96%) as a colorless liquid.

^{1}H NMR (500 MHz, $CDCl_3$): δ = 0.04 (s, 12 H, CH_3), 0.88 (s, 18 H, *t*Bu), 2.85 (quint., $J \approx 5.5$ Hz, 1 H, 1-H), 3.50, 3.59 (AB part of ABX system, J_{AB} = 9.8 Hz, J_{AX} = 5.2 Hz, J_{BX} = 5.7 Hz, 2 H each, 2-H) ppm; ^{13}C NMR (125 MHz, $CDCl_3$): δ = -5.3, -3.4 (2 q, $SiCH_3$), 18.4 [s, Si*C*$(CH_3)_3$], 26.0 [q, SiC$(\mathit{C}H_3)_3$], 54.5 (d, C-1), 64.9 (t, C-2) ppm; IR (ATR): $\tilde{\nu}$ = 3370 (N-H), 1250 (C-O) cm^{-1}; HRMS (ESI-TOF): m/z calcd. for $C_{15}H_{38}NO_2Si_2$ $[M + H]^+$: 320.2436; found: 320.2447.

(2*S*,3*S*,4*S*,5*S*)-4-(*tert*-Butyldimethylsiloxy)-2,5-bis[(*tert*-butyldimethylsiloxy)-methyl]-6,6-dimethyltetrahydro-2*H*-pyran-3-hexanamide (**4**)

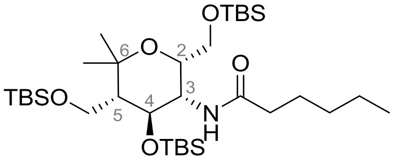

4

According to **GP-1**, protected aminopyran **3** (208 mg, 0.38 mmol) was dissolved in CH_2Cl_2 (2.9 mL) and the solution was cooled to 0 °C under argon atmosphere. After addition of Et_3N (0.1 mL, 0.59 mmol) and hexanoyl chloride (61 mg, 64 µL, 0.45 mmol) the reaction mixture was stirred at 0 °C for 2.5 h, and at rt overnight. H_2O (5 mL) was then added to the reaction mixture and the aqueous phase was extracted with CH_2Cl_2 (3 × 10 mL). The combined organic phases were dried with Na_2SO_4 and the solvents removed in vacuo. The crude product was purified by flash column chromatography (silica gel, hexanes/EtOAc, 6:1) affording **4** (227 mg, 93%) as a pale yellow oil.

$[\alpha]_D^{22}$ = +1.20 (c = 1.51, $CHCl_3$); ^{1}H NMR (500 MHz, $CDCl_3$): δ = 0.015, 0.018, 0.028, 0.033, 0.101, 0.174 (6 s, 3 H each, CH_3), 0.87– 0.90 (m, 30 H, *t*Bu, CH_3), 1.18 (s, 3 H, CH_3), 1.27 – 1.34 (m, 4 H, CH_2), 1.49 (s, 3 H, CH_3), 1.59 – 1.66 (m, 3 H, 5-H, CH_2), 2.03– 2.18 (m, 2 H, CH_2), 3.44 (dd, *J* = 7.2, 10.2 Hz, 1 H, 5-CH_2), 3.60 (d, *J* = 5.9 Hz, 2 H, 2-CH_2), 3.71 – 3.78 (m, 2 H, 4-H, 5-CH_2), 4.15 – 4.17 (m, 1 H, 2-H), 4.24 (m_c, 1 H, 3-H), 5.80 (d, *J* = 7.5 Hz, 1 H, NH) ppm; ^{13}C NMR (125 MHz, $CDCl_3$): δ = -4.7, -5.01, -5,02, -5.15, -5.26, -5,27 (6 q, $SiCH_3$), 14.1 (q, CH_3), 18.0, 18.8, 18.4 [3 s, Si*C*(CH_3)$_3$], 22.6 (t, CH_2), 25.4 (t, CH_2), 25.9, 25.96, 26.0 [3 q, SiC(*C*H_3)$_3$], 27.3, 28.3 (2 q, CH_3), 31.7 (t, CH_2), 37.1 (t, CH_2), 49.5 (d, C-5), 50.2 (d, C-3), 62.8 (t, 5-CH_2), 63.5 (t, 2-CH_2), 67.2 (d, C-2), 68.9 (d, C-4), 74.2 (s, C-6), 172.5 (s, C=O) ppm; IR (ATR): $\tilde{\nu}$ = 2960 (N-H), 2930-2860 (C-H), 1680 (C=O), 1250 (C-O), 1070 (C-O-C)

cm^{-1}; HRMS (ESI-TOF): m/z calcd. for $C_{33}H_{72}NO_5Si_3$ [M + H]$^+$: 646.4713; found: 646.4705; $C_{33}H_{71}NNaO_5Si_3$ [M + Na]$^+$: 668.4532; found: 668.4528; Elemental Analysis calcd. (%) for $C_{33}H_{71}NO_5Si_3$ (646.2): C 61.34, H 11.07, N 2.17; found: C 61.15, H 11.07, N 1.99.

(2*S*,3*R*,4*S*,5*S*)-4-Hydroxy-2,5-bis(hydroxymethyl)-6,6-dimethyltetrahydro-2*H*-pyran-3-hexanamide (**5**)

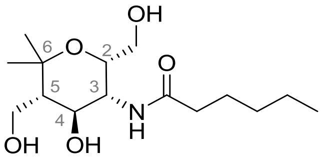

5

According to **GP-3**, to a stirred solution of **4** (83 mg, 0.13 mmol) in THF (1 mL) at 0 °C, HF·pyridine (1.1 mL, 1.1 mmol) was added. After warming up to rt, the reaction mixture was stirred for 24 h. H_2O (5 mL) was added and the aqueous phase was extracted with CH_2Cl_2 (3 × 10 mL). The combined organic phases were dried with Na_2SO_4 and the solvents were removed in vacuo. The crude product was purified by flash column chromatography (silica gel, CH_2Cl_2/MeOH, 95:5) affording **5** (49 mg, quant.) as a colorless solid.

m.p. 108 – 110 ºC; $[\alpha]_D^{22}$ = +42.7 (c = 1.49, MeOH); ^{1}H NMR (500 MHz, CD_2Cl_2): δ = 0.90 (t, J = 6.9 Hz, 3 H, CH_3), 1.06, 1.28 (2 s, 3 H each, CH_3), 1.30 – 1.36 (m, 2 H, CH_2), 1.57 – 1.65 (m, 4 H, CH_2), 1.84 – 1.89 (m, 1 H, 5-H), 2.20 – 2.25 (m, 2 H, CH_2), 3.53 – 3.60 (m, 1 H, 2-CH_2), 3.67 – 3.72 (m, 1 H, 5-CH_2), 3.75 (B part of ABX system, J_{AB} = 11.0, Hz, J_{BX} = 8.0 Hz, 1 H, 5-CH_2), 3.80 (m_c, 1 H, 4-H), 3.81 – 3.86 (m, 2 H, 2-H, 2-CH_2), 3.95 – 3.98 (m, 1 H, 3-H), 7.98 (s, 1 H, NH) ppm; ^{13}C NMR (125 MHz CD_2Cl_2): δ = 14.6 (q, CH_3), 24.3 (q, CH_3), 26.0 (t, CH_2), 26.8 (q, CH_3), 27.3 (t, CH_2), 32.2 (t, CH_2), 37.4 (t, CH_2), 48.9 (d, C-5), 55.2 (d, C-3), 63.6 (t, 5-CH_2), 63.7 (t, 2-

CH_2), 71.5 (d, C-2), 74.0 (d, C-4), 76.0 (s, C-6), 129.0 (s, C=O) ppm; IR (ATR): $\tilde{\nu}$ = 3450 (N-H), 3310 (O-H), 2960-2860 (C-H), 1620 (C=O), 1230 (C-O), 1080 (C-O-C) cm^{-1}; HRMS (ESI-TOF): m/z calcd. for $C_{15}H_{29}NNaO_5$ $[M + Na]^+$: 326.1938; found: 326.1966; Elemental analysis calcd. (%) $C_{15}H_{29}NO_5$ (303.4): C 59.38, H 9.63, N 4.62; found: C 59.15, H 9.63, N 4.38.

Divalent amide 10

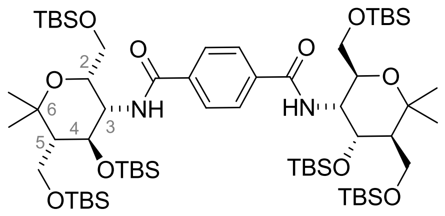

10

According to **GP-1**, protected aminopyran **3** (200 mg, 0.36 mmol) was dissolved in CH_2Cl_2 (3.2 mL) and the solution was cooled to 0 °C. After addition of Et_3N (0.1 mL, 0.73 mmol) and terephthaloyl chloride (**7**, 45 mg, 0.22 mmol) the reaction mixture was stirred at 0 °C for 1 h and at rt for 20 h. H_2O (5 mL) was added and the aqueous layer was extracted with CH_2Cl_2 (3 × 10mL). The combined organic layers were dried with Na_2SO_4 and the solvents were removed in vacuo. The crude product was purified by flash column chromatography (silica gel, hexanes/EtOAc, 6:1) affording **10** (223 mg, quant.) as colorless solid.

Melting range: 105 – 110 °C; $[\alpha]_D^{22}$ = +33.8 (c = 0.73, $CHCl_3$); ^1H NMR (500 MHz, $CDCl_3$): δ = -0.20, -0.10 (2 s, 6 H each, CH_3), -0.01 (s, 12 H, CH_3), 0.14, 0.24 (2 s, 6 H each, CH_3), 0.75, 0.83, 0.92 (3 s, 18 H each, *t*Bu), 1.21, 1.54 (2 s, 6 H each, CH_3), 1.69 (bt, *J* ≈ 7.6 Hz, 2 H, 5-H), 3.50 (dd, *J* = 8.6, 10.1 Hz, 2 H, 5-CH_2), 3.72 (d, *J* = 5.5 Hz, 4 H, 2-CH_2), 3.79 (dd, *J* = 7.1, 10.1 Hz, 2 H, 5-CH_2), 3.92 – 3.96 (m, 2 H, 3-H), 4.26 (dt, *J* = 2.1, 5.5 Hz, 2 H, 2-H), 4.52 – 4.55 (m, 2 H, 4-H), 6.82 (d, *J* = 6.8 Hz,

2 H, NH), 7.78 (s, 4 H, Ar) ppm; ^{13}C NMR (125 MHz, $CDCl_3$): δ = -5.45, -5.41, -5.28, -5.25, -4.93, -4.65 (6 q, $SiCH_3$), 18.0, 18.1, 18.5 [3 s, Si*C*$(CH_3)_3$], 25.88, 25.91, 25.95 [3 q, SiC(*C*$H_3)_3$], 27.3, 28.2 (2 q, CH_3), 49.0 (d, C-5), 51.7 (d, C-3), 62.4 (t, 5-CH_2), 64.0 (t, 2-CH_2), 66.5 (d, C-2), 67.9 (d, C-4), 74.3 (s, C-6), 127.2 (d, Ar), 137.4 (s, Ar), 166.1 (s, C=O) ppm; IR (ATR): $\tilde{\nu}$ = 3380 (N-H), 2860 (C-H), 1670 (C=O), 1525 (C=C), 1250 (C-O), 1075 (C-O-C) cm^{-1}; HRMS (ESI-TOF): m/z calcd. for $C_{62}H_{124}N_2NaO_{10}Si_6$ [M + Na]$^+$: 1248.7786; found: 1248.7760; Elemental analysis calcd. (%) $C_{62}H_{124}N_2O_{10}Si_6$ (1226.2): C 60.73, H 10.19, N 2.28; found: C 60.85, H 10.24, N 2.36.

Divalent amide 13

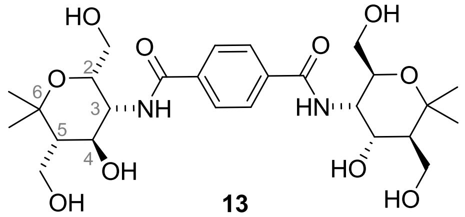

13

According to **GP-3**, to a stirred solution of **10** (116 mg, 0.095 mmol) in THF (0.7 mL) at 0 °C, HF·pyridine (0.15 mL, 0.15 mmol) was added. After warming up to rt, the reaction mixture was stirred for 22 h. MeOH was added to the mixture and the solvents were removed in vacuo. The crude product was purified by flash column chromatography (silica gel, CH_2Cl_2/MeOH, 9:1) affording **13** (52 mg, quant.) as a slightly pink solid.

m.p. 210 °C; $[\alpha]_D^{22}$ = +45.2 (c = 0.43, MeOH); ^{1}H NMR (700 MHz, CD_3OD): δ = 1.33, 1.46 (2 s, 6 H each, CH_3), 1.78 (m_c, 2 H, 5-H), 3.51, 3.56 (AB part of ABX system, J_{AB} = 11.6 Hz, J_{AX} = 4.8 Hz, J_{BX} = 6.9 Hz, 2 H each, 2-CH_2), 3.67 (dd, J = 4.2, 11.1 Hz, 2 H, 5-CH_2), 3.96 – 3.99 (m, 4 H, 5-CH_2, 4-H), 4.18 – 4.21 (m, 4 H, 3-H, 2-H), 7.91 (s, 4 H, Ar) ppm; ^{13}C NMR (175 MHz, CD_3OD): δ = 26.3, 27.7 (2 q, CH_3), 48.8 (d, C-5),

54.2 (d, C-3), 63.2 (t, 2-CH_2), 63.3 (t, 5-CH_2), 70.9 (d, C-2), 74.0 (d, C-4), 75.9 (s, C-6), 128.6 (d, Ar), 138.4 (s, Ar), 169.5 (s, C=O) ppm; IR (ATR): $\tilde{\nu}$ = 3290 (O-H, N-H), 2980-2910 (C-H), 1725 (C=O), 1440 (C=C), 1250 (C-O), 1070 (C-O-C) cm^{-1}; HRMS (ESI-TOF): m/z calcd. for $C_{26}H_{40}N_2NaO_{10}$ $[M+Na]^+$: 563.2575; found: 563.2581.

Divalent amide 11

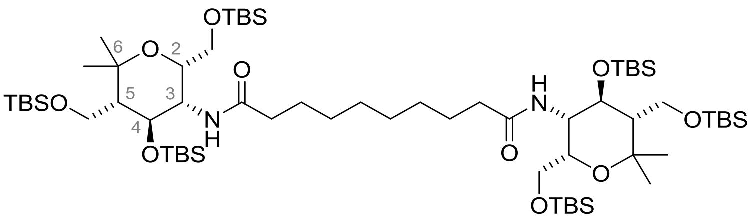

11

According to **GP-1**, protected aminopyran **3** (200 mg, 0.36 mmol) was dissolved in CH_2Cl_2 (3.2 mL) and the solution was cooled to 0 °C. After addition of Et_3N (0.1 mL, 0.73 mmol) and sebacoyl chloride (**8**, 46.7 µL, 0.219 mmol) the reaction mixture was stirred at 0 °C for 1 h and at rt for 20 h. Water (5 mL) was added and the aqueous layer was extracted with CH_2Cl_2 (3 × 10 mL). The combined organic layers were dried with Na_2SO_4 and the solvents were removed under reduced pressure. The crude product was purified by flash column chromatography (silica gel, hexanes/EtOAc, 6:1) affording **11** (132 mg, 58%) as colorless oil.

$[\alpha]_D^{22}$ = +32.0 (c = 0.95, $CHCl_3$); ^1H NMR (500 MHz, $CDCl_3$): δ = 0.009, 0.013, 0.022, 0.028, 0.10, 0.17 (6 s, 6 H each, CH_3), 0.87 (s, 36 H, *t*Bu), 0.89 (s, 18 H, *t*Bu), 1.18 (s, 6 H, CH_3), 1.28 (bs, 8 H, CH_2), 1.48 (s, 6 H, CH_3), 1.57 – 1.62 (m, 6 H, CH_2, 5-H), 2.04 – 2.16 (m, 4 H, CH_2), 3.43 (dd, *J* = 7.4, 10.2 Hz, 2 H, 5-CH_2), 3.59 (d, *J* = 5.9 Hz, 4 H, 2-CH_2), 3.70 – 3.77 (m, 4 H, 5-CH_2, 3-H), 4.15 (dt, *J* = 2.1, 5.9 Hz, 2 H, 2-H), 4.22 – 4.25 (m, 2 H, 4-H), 5.80 (d, *J* = 7.8 Hz, 2 H, NH) ppm; ^{13}C NMR (125 MHz, $CDCl_3$): δ = -5.32, -5.32, -5.31, -5.21, -5.06, -4.77 (6 q, $SiCH_3$), 17.9, 18.2, 18.3 [3 s,

Si*C*(CH_3)$_3$], 25.7 (t, CH_2), 25.87, 25.90, 25.97 [3 q, SiC(*C*H_3)$_3$], 27.3, 28.2 (2 q, CH_3), 29.4, 29.6 (2 t, CH_2), 37.0 (t, CH_2), 49.4 (d, C-5), 50.2 (d, C-3), 62.7 (t, 5-CH_2), 63.5 (t, 2-CH_2), 67.1 (d, C-2), 68.8 (d, C-4), 74.2 (s, C-6), 172.4 (s, C=O) ppm; IR (ATR): $\tilde{\nu}$ = 3275 (N-H), 2970-2855 (C-H), 1640 (C=O), 1230 (C-O), 1070 (C-O-C) cm^{-1}; HRMS (ESI-TOF): m/z calcd. for $C_{64}H_{136}N_2NaO_{10}Si_6$ $[M + Na]^+$: 1283.8703; found: 1283.8759.

Divalent amide 14

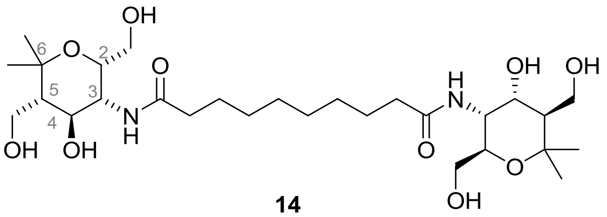

14

According to **GP-3**, to a stirred solution of **11** (68 mg, 0.054 mmol) in THF (0.4 mL) at 0 °C, HF·pyridine (89.6 µL, 0.089 mmol) was added. After warming up to rt, the reaction mixture was stirred for 24 h. MeOH was added to the reaction mixture and the solvents were removed in vacuo. The crude product was purified by flash column chromatography (silica gel, CH_2Cl_2/MeOH 9:1 to 4:1) affording **14** (25 mg, 80%) as a colorless solid.

m.p. 215 – 217 °C; $[\alpha]_D^{22}$ = +54.9 (c = 1.24, MeOH); ^1H NMR (500 MHz, CD_3OD): δ = 1.23 (s, 6 H, CH_3), 1.34 (s, 8 H, CH_2), 1.39 (s, 6 H, CH_3), 1.61 – 1.63 (m, 4 H, CH_2), 1.78 (td, *J* ≈ 5.5, 7.8 Hz, 2 H, 5-H), 2.23 (t, *J* = 7.2 Hz, 4 H, CH_2), 3.46 (d, *J* = 6.1 Hz, 4 H, 2-CH_2), 3.65 (dd, *J* = 5.4, 11.2 Hz, 2 H, 5-CH_2), 3.77 (dd, *J* = 7.8, 5.2 Hz, 2 H, 4-H), 3.83 (dd, *J* = 5.7, 11.2 Hz, 2 H, 5-CH_2), 3.91 – 3.94 (m, 2 H, 3-H), 4.05 (dt, *J* = 3.9, 6.1 Hz, 2 H, 2-H) ppm; ^{13}C NMR (125 MHz, CD_3OD): δ = 25.4 (q, CH_3), 26.9 (t, CH_2), 27.3 (q, CH_3), 30.0, 30.2, 37.1 (3 t, CH_2), 49.5 (d, C-5), 55.1 (d, C-3), 62.9 (t, 5-CH_2), 63.0 (t, 2-CH_2), 71.1 (d, C-2), 73.6 (d, C-4), 76.0 (s, C-6), 128.8 (s)* ppm;

*Signal could not be attributed. C=O singlet could not be detected; IR (ATR): $\tilde{\nu}$ = 3320 (O-H, N-H), 2970-2890 (C-H), 1675 (C=O), 1245 (C-O), 1070 (C-O-C) cm^{-1}; HRMS (ESI-TOF): m/z calcd. for $C_{28}H_{52}N_2NaO_{10}$ $[M + Na]^+$: 601.3671; found: 601.3642.

Divalent amide 12

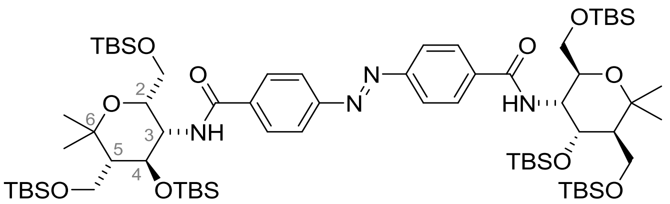

12

According to **GP-1**, protected aminopyran **3** (103 mg, 0.19 mmol) was dissolved in CH_2Cl_2 (2 mL) and the solution was cooled to 0 °C. After addition of Et_3N (0.1 mL, 0.73 mmol) and acid chloride **9** (26 mg, 0.08 mmol) the reaction mixture was stirred at 0 °C for 1 h and at rt for 24 h. H_2O (5 mL) was added and the aqueous phase was extracted with CH_2Cl_2 (3 × 10 mL). The combined organic phases were dried with Na_2SO_4 and the solvents removed in vacuo. The crude product was purified by flash column chromatography (silica gel, hexanes/EtOAc, 6:1) affording **12** (114 mg, quant.) as a pink solid.

Melting range: 170 – 175 °C; $[\alpha]_D^{22}$ = +40.2 (c = 1.13, $CHCl_3$); ^1H NMR (500 MHz, $CDCl_3$): δ = -0.16, -0.08, 0.02, 0.07, 0.16, 0.26 (6 s, 6 H each, CH_3), 0.76, 0.85, 0.93 (3 s, 18 H each, *t*Bu), 1.22, 1.25 (2 s, 6 H each, CH_3), 1.66 – 1.69 (m, 2 H, 5-H), 3.53 (dd, *J* = 8.6, 10.1 Hz, 2 H, 5-CH_2), 3.74, 3.76 (AB part of ABX system, J_{AB} = 10.9 Hz, J_{AX} = 5.1 Hz, J_{BX} = 5.9 Hz, 2 H each, 2-CH_2), 3.81 (dd, *J* = 7.1, 10.1 Hz, 2 H, 5-CH_2), 3.96 – 3.99 (m, 2 H, 3-H), 4.28 (dt, *J* ≈ 2.0, 5.5 Hz, 2 H, 2-H), 4.54 (t, *J* = 2.3 Hz, 2 H, 4-H), 6.84 (d, *J* = 7.0 Hz, 2 H, NH), 7.91 (d, *J* = 8.5 Hz, 4 H, Ar), 7.98 (d, *J* = 8.5 Hz, 4 H, Ar) ppm; ^{13}C NMR (125 MHz, $CDCl_3$): δ = -5.45, -5.43, -5.25, -5.22, -4.93, -4.65 (6

q, $SiCH_3$), 18.0, 18.2, 18.5 [3 s, Si*C*(CH_3)$_3$], 25.89, 25.94, 25.96 [3 q, SiC(*C*H_3)$_3$], 27.3, 28.2 (2 q, CH_3), 49.0 (d, C-5), 51.6 (d, C-3), 62.5 (t, 5-CH_2), 64.0 (t, 2-CH_2), 66.7 (d, C-2), 67.9 (d, C-4), 74.3 (s, C-6), 123.2, 128.1 (2 d, Ar), 137.1, 154.1 (2 s, Ar), 166.1 (s, C=O) ppm; IR (ATR): $\tilde{\nu}$ = 3310 (N-H), 2960-2850 (C-H), 1735 (C=O), 1470 (C=C), 1250 (C-O), 1060 (C-O-C) cm^{-1}; HRMS (ESI-TOF): m/z calcd. for $C_{68}H_{128}N_4NaO_{10}Si_6$ $[M+Na]^+$: 1351.8133; found: 1351.8136.

Divalent amide 15

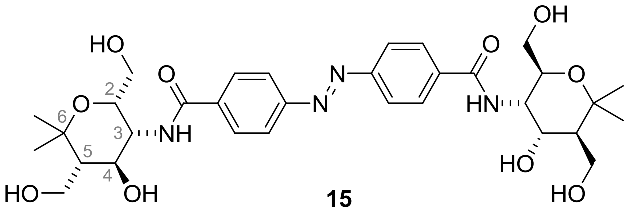

15

According to **GP-3**, to a stirred solution of **12** (44 mg, 0.033 mmol) in THF (0.3 mL) at 0 °C, HF·pyridine (40 µL, 0.397 mmol) was added. After warming up to rt, the reaction mixture was stirred for 24 h. MeOH was added to the mixture and the solvents were removed in vacuo. The crude product was purified by flash column chromatography (silica gel, CH_2Cl_2/MeOH, 95:5) affording **15** (27 mg, quant.) as an orange solid.

m.p. 249 ºC; $[\alpha]_D^{22}$ = +71.8 (c = 0.66, MeOH); ^{1}H NMR (700 MHz, CD_3OD): δ = 1.34, 1.47 (2 s, 6 H each, CH_3), 1.79 (m_c, 2 H, 5-H), 3.54, 3.59 (AB part of ABX system, J_{AB} = 11.7 Hz, J_{AX} = 4.4 Hz, J_{BX} = 6.7 Hz, 2 H each, 2-CH_2), 3.70 (dd, *J* = 4.1, 11.2 Hz, 2 H, 5-CH_2), 3.97 – 4.02 (m, 4 H, 5-CH_2, 4-H), 4.18 – 4.23 (m, 4 H, 2-H, 3-H), 8.01 (d, *J* = 8.6 Hz, 4 H, Ar), 8.04 (d, *J* = 8.6 Hz, 4 H, Ar) ppm; ^{13}C NMR (175 MHz, CD_3OD): δ = 26.3, 27.7 (2 q, CH_3), 48.8 (d, C-5), 54.2 (d, C-3), 63.2 (t, 2-CH_2), 63.3 (t, 5-CH_2), 70.9 (d, C-2), 74.1 (d, C-4), 75.9 (s, C-6), 124.0, 129.7 (2 d, Ar), 138.1, 155.6 (2 s, Ar), 169.5 (s, C=O) ppm; IR (ATR): $\tilde{\nu}$ = 3320 (O-H, N-H), 2960-2855 (C-H), 1730

(C=O), 1440 (C=C), 1250 (C-O), 1060 (C-O-C) cm^{-1}; HRMS (ESI-TOF): m/z calcd. for $C_{32}H_{44}N_4NaO_{10}$ $[M + Na]^+$: 667.2944; found: 667.2943; Elemental analysis calcd. (%) $C_{32}H_{44}N_4O_{10}$ (644.7) + 6 H_2O: C 51.05, H 7.50, N 7.44; found: C 51.06, H 6.08, N 7.11.

Divalent amide 18

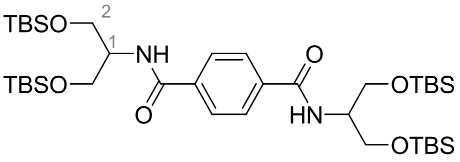

18

According to **GP-1**, protected serinol **16** (200 mg, 0.626 mmol) was dissolved in CH_2Cl_2 (5.4 mL) and the solution was cooled to 0 °C. After addition of Et_3N (0.17 mL, 1.25 mmol) and terephthaloyl chloride (**7**, 76 mg, 0.38 mmol) the reaction mixture was stirred at 0 °C for 1 h and at rt for 17 h. H_2O (10 mL) was added to the reaction mixture and the aqueous layer was extracted with CH_2Cl_2 (3 × 20 mL). The combined organic layers were dried with Na_2SO_4 and the solvents removed in vacuo. The crude product was purified by flash column chromatography (silica gel, hexanes/EtOAc, 5:1) affording **18** (201 mg, 83%) as colorless solid.

m.p. 142 ºC; ^{1}H NMR (500 MHz, $CDCl_3$): δ = 0.07, 0.09 (2 s, 12 H each, CH_3), 0.91 (s, 36 H, *t*Bu), 3.65 (dd, *J* = 6.4, 9.6 Hz, 4 H, 2-H), 3.87 (dd, *J* = 3.5, 9.6 Hz, 4 H, 2-H), 4.13 – 4.19 (m, 2 H, 1-H), 6.58 (d, *J* = 8.3 Hz, 2 H, NH), 7.80 (s, 4 H, Ar) ppm; ^{13}C NMR (125 MHz, $CDCl_3$): δ = -5.33, -5.25 (2 q, $SiCH_3$), 18.4 [s, Si*C*$(CH_3)_3$], 26.0 [q, $SiC(CH_3)_3$], 52.1 (d, C-1), 60.5 (t, C-2), 127.3 (d, Ar), 137.4 (s, Ar), 166.0 (s, C=O) ppm; IR (ATR): $\tilde{\nu}$ = 3275 (N-H), 2880 (C-H), 1630 (C=O), 1545 (C=C), 1255 (C-O) cm^{-1}; HRMS (ESI-TOF): m/z calcd. for $C_{38}H_{76}N_2NaO_6Si_4$ $[M + Na]^+$: 791.4673; found:

791.4743; Elemental analysis calcd. (%) $C_{38}H_{76}N_2O_6Si_4$ (769.4): C 59.32, H 9.96, N 3.64; found: C 59.25, H 9.93, N 3.52.

Divalent amide 21

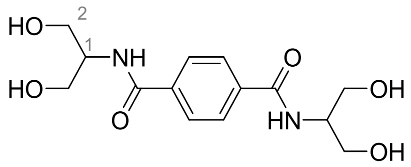

21

According to **GP-4**, to a stirred suspension of **18** (200 mg, 0.260 mmol) in 2-propanol (0.9 mL), AcCl (10 µL, 0.16 mmol) was added at 0 °C. The reaction mixture was stirred at rt for 1.5 h. All volatiles were removed in vacuo affording **21** (73 mg, 90%) as a colorless solid.

Melting range: 178 – 182 °C; ^{1}H NMR (500 MHz, DMSO-d$_6$): δ = 3.47 – 3.57 (m, 8 H, 2-H), 3.93 – 4.02 (m, 2 H, 1-H), 4.68 (t, *J* = 5.7 Hz, 4 H, OH), 7.93 (s, 4 H, Ar), 8.09 (d, *J* = 8.1 Hz, 1 H, NH) ppm; ^{13}C NMR (125 MHz, DMSO-d$_6$): δ = 54.0 (d, C-1), 60.4 (t, C-2), 127.2 (d, Ar), 136.8 (s, Ar), 165.6 (s, C=O) ppm; IR (ATR): $\tilde{\nu}$ = 3290-3230 (O-H, N-H), 2970-2840 (C-H), 1630 (C=O), 1555 (C=C), 1230 (C-O) cm^{-1}; HRMS (ESI-TOF): m/z cald. for $C_{14}H_{20}N_2NaO_6$ [M + Na]$^+$: 335.1214; found: 335.1216; Elemental analysis calcd. (%) $C_{14}H_{20}N_2O_6$ (312.3): C 53.84, H 6.45, N 8.97; found: C 53.81, H 6.59, N 8.99.

Divalent amide 19

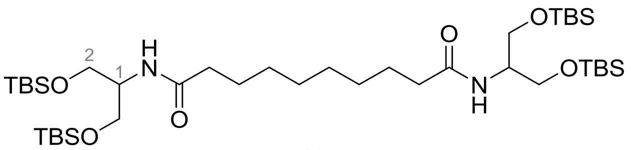

19

According to **GP-1**, protected serinol **16** (200 mg, 0.626 mmol) was dissolved in CH_2Cl_2 (5.4 mL) and the solution was cooled to 0 °C. After addition of Et_3N (0.17 mL, 1.25 mmol) and sebacoyl chloride (**8**, 90 mg, 0.38 mmol) the reaction mixture was stirred at 0 °C for 1 h and at rt for 18 h. H_2O (10 mL) was added to the reaction mixture and the aqueous layer was extracted with CH_2Cl_2 (3 × 20 mL). The combined organic layers were dried with Na_2SO_4 and the solvents removed in vacuo. The crude product was purified by flash column chromatography (silica gel, hexanes/EtOAc, 5:1) affording **19** (190 mg, 75%) as colorless solid.

m.p. 75 ºC; ^{1}H NMR (500 MHz, $CDCl_3$): δ = 0.05, 0.06 (2 s, 12 H each, CH_3), 0.89 (s, 36 H, *t*Bu), 1.29 (bs, 8 H, CH_2), 1.58 – 1.61 (m, 4 H, CH_2), 2.12 – 2.17 (m, 4 H, CH_2), 3.52 (dd, *J* = 6.4, 9.6 Hz, 4 H, 2-H), 3.73 (dd, *J* = 3.6, 9.6 Hz, 4 H, 2-H), 3.92 – 3.98 (m, 2 H, 1-H), 5.73 (d, *J* = 8.2 Hz, 2 H, NH) ppm; ^{13}C NMR (125 MHz, $CDCl_3$): δ = -5.4, -5.3 (q, $SiCH_3$), 18.3 [s, Si*C*(CH_3)$_3$], 25.8 (t, CH_2), 25.9 [q, SiC(*C*H_3)$_3$], 29.2, 29.3, 37.0 (3 t, CH_2), 51.4 (d, C-1), 60.6 (t, C-2), 172.6 (s, C=O) ppm; IR (ATR): $\tilde{\nu}$ = 3290 (N-H), 2955-2855 (C-H), 1640 (C=O), 1250 (C-O) cm^{-1}; HRMS (ESI-TOF): m/z calcd. for $C_{40}H_{88}N_2NaO_6Si_4$ $[M + Na]^+$: 827.5606; found: 827.5625; Elemental analysis calcd. (%) $C_{40}H_{88}N_2O_6Si_4$ (805.5) + 1 H_2O: C 58.34, H 11.02, N 3.40**;** found: C 58.37, H 9.73, N 3.22.

Divalent amide 22

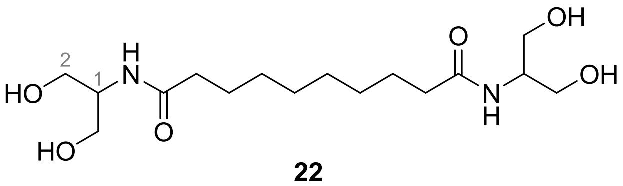

22

According to **GP-4**, to a stirred solution of **19** (200 mg, 0.248 mmol) in isopropanol (0.9 mL), AcCl (10.6 µL, 0.160 mmol) was added at 0 °C. The reaction mixture was stirred at rt for 2.5 h. All volatiles were removed in vacuo affording **22** (88 mg, quant.) as a colorless solid.

m.p. 170 – 171 °C; ^{1}H NMR (500 MHz, DMSO-d$_6$): δ = 1.20 – 1.25 (m, 8 H, CH_2), 1.43 – 1.50 (m, 4 H, CH_2), 2.06 (t, *J* = 7.5 Hz, 4 H, CH_2), 3.37 (bd, *J* = 5.7 Hz, 8 H, 2-H), 3.62 – 3.73 (m, 2 H, 1-H), 4.57 (bs, 4 H, OH), 7.43 (d, *J* = 8.1 Hz, 2 H, NH) ppm; ^{13}C NMR (125 MHz, DMSO-d$_6$): δ = 25.3, 28.6, 28.7, 35.4 (4 t, CH_2), 52.7 (d, C-1), 60.2 (t, C-2), 172.1 (s, C=O) ppm; IR (ATR): $\tilde{\nu}$ = 3300 (O-H, N-H), 2920-2850 (C-H), 1640 (C=O), 1255 (C-O) cm^{-1}; HRMS (ESI-TOF): m/z calcd. for $C_{16}H_{32}N_2NaO_6$ [M + Na]$^+$: 371.2153; found: 371.2154; Elemental analysis calcd. (%) $C_{16}H_{32}N_2O_6$ (348.4): C 55.15, H 9.26, N 8.04; found: C 55.01, H 9.31, N 7.88.

Divalent amide 20

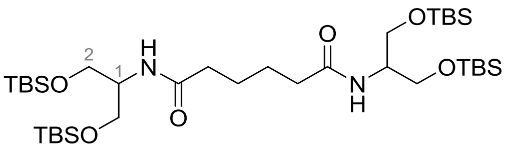

20

According to **GP-1**, protected serinol **16** (2.00 g, 6.26 mmol) was dissolved in CH_2Cl_2 (54 mL) and the solution was cooled to 0 °C. After addition of Et_3N (1.75 mL, 12.5 mmol) and adipoyl chloride (**17**, 687 mg, 3.75 mmol) the reaction mixture was stirred at 0 °C for 1 h and at rt for 17 h. H_2O (100 mL) was added to the reaction and the

aqueous layer was extracted with CH_2Cl_2 (3 × 200 mL). The combined organic layers were dried with Na_2SO_4 and the solvents removed in vacuo. The crude product was purified by flash column chromatography (silica gel, hexanes/EtOAc, 5:1) affording **20** (1.45 g, 62%) as colorless solid.

m.p. 101 °C; ^{1}H NMR (500 MHz, $CDCl_3$): δ = 0.04, 0.05 (2 s, 12 H each, CH_3), 0.88 (s, 36 H, *t*Bu), 1.61 – 1.68 (m, 4 H, CH_2), 2.16 – 2.19 (m, 4 H, CH_2), 3.52 (dd, *J* = 6.4, 9.6 Hz, 4 H, 2-H), 3.72 (dd, *J* = 3.7, 9.6 Hz, 4 H, 2-H), 3.94 (m_c, 2 H, 1-H), 5.77 (d, *J* = 8.5 Hz, 2 H, NH) ppm; ^{13}C NMR (125 MHz, $CDCl_3$): δ = -5.35, -5.27 (2 q, $SiCH_3$), 18.4 [s, Si*C*(CH_3)$_3$], 25.3 (t, CH_2), 26.0 [q, SiC(*C*H_3)$_3$], 36.5 (t, CH_2), 51.6 (d, C-1), 60.6 (t, C-2), 172.2 (s, C=O) ppm; IR (ATR): $\tilde{\nu}$ = 3295 (N-H), 2960-2855 (C-H), 1710 (C=O), 1250 (C-O) cm^{-1}; HRMS (ESI-TOF): m/z calcd. for $C_{36}H_{80}N_2NaO_6Si_4$ [M + Na]$^+$: 771.4986; found: 771.4960; Elemental analysis calcd. (%) $C_{36}H_{80}N_2O_6Si_4$ (749.4): C 57.70, H 10.76, N 3.74; found: C 57.88, H 10.63, N 3.71.

Divalent amide 23

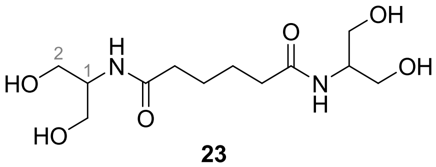

23

According to **GP-4**, to a stirred solution of **20** (200 mg, 0.267 mmol) in 2-propanol (0.9 mL), AcCl (11.4 µL, 0.160 mmol) was added at 0 °C. The reaction mixture was stirred at rt for 1 h and 40 min. All volatiles were removed in vacuo affording **23** (76 mg, 97%) as a colorless solid.

m.p. 174 – 175 ºC; ^{1}H NMR (500 MHz, DMSO-d_6): δ = 1.42 – 1.47 (m, 4 H, CH_2), 2.07 (t, *J* = 6.6 Hz, 4 H, CH_2), 3.38 (d, *J* = 5.7 Hz, 8 H, 2-H), 3.65 – 3.72 (m, 2 H, 1-H), 4.27 (bs, 4 H, OH), 7.45 (d, *J* = 8.1 Hz, 2 H, NH) ppm; ^{13}C NMR (125 MHz, DMSO-d_6): δ = 25.0, 35.2 (2 t, CH_2), 52.8 (d, C-1), 60.2 (t, C-2), 172.0 (s, C=O) ppm;

IR (ATR): $\tilde{\nu}$ = 3295 (O-H, N-H), 2965-2855 (C-H), 1635 (C=O), 1260 (C-O) cm^{-1}; HRMS (ESI-TOF): m/z calcd. for $C_{12}H_{24}N_2NaO_6$ $[M + Na]^+$: 315.1521; found: 315.1526; Elemental analysis calcd. (%) $C_{12}H_{24}N_2O_6$ (292.3): C 49.30, H 8.28, N 9.85; found: C 49.36, H 8.30, N 9.55.

Divalent amide 25

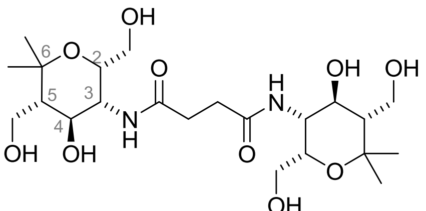

25

According to **GP-2**, aminopyran **1** (50 mg, 0.24 mmol), succinic acid **24** (11 mg, 0.97 mmol) and HATU (93 mg, 0.24 mmol) were dissolved in DMF (2.0 mL). After addition of Et_3N (0.4 mL, 2.8 mmol), the reaction mixture was stirred at rt under argon atmosphere. After 24 h the solvent was removed in vacuo. The crude product was purified by flash column chromatography on silica gel (DCM/MeOH 9:1 to 4:1) affording **25** (35 mg, 73%) as a colorless solid.

Melting range: 185 – 201 °C; $[\alpha]_D^{22}$ = +47.2 (c = 1.40, MeOH); ^1H NMR (500 MHz, CD_3OD): δ = 1.21, 1.40 (2 s, 6 H each, CH_3), 1.77 – 1.81 (m, 2 H, 5-H), 2.54 (bs, 4 H, CH_2), 3.48 (d, J = 6.1 Hz, 4 H, 2-CH_2), 3.66 (dd, J = 5.5, 11.2 Hz, 2 H, 5-CH_2), 3.79 – 3.83 (m, 4 H, 5-CH_2, 4-H), 3.92 (dd, J = 3.9, 5.2 Hz, 2 H, 3-H), 4.06 (dt, J = 3.9, 6.1 Hz, 2 H, 2-H) ppm; ^{13}C NMR (125 MHz, CD_3OD): δ = 25.5, 27.3 (2 q, CH_3), 32.1 (t, CH_2), 49.4 (d, C-5), 55.2 (d, C-3), 62.7 (t, 2-CH_2), 62.8 (t, 5-CH_2), 71.0 (d, C-2), 73.1 (d, C-4), 76.0 (s, C-6), 175.2 (C=O) ppm; IR (ATR): $\tilde{\nu}$ = 3410-3260 (O-H, N-H), 2970-2890 (C-H), 1640 (C=O), 1230 (C-O), 1075 (C-O-C) cm^{-1}; HRMS (ESI-TOF): m/z calcd. for $C_{22}H_{40}N_2NaO_{10}$ $[M + Na]^+$: 515.2575; found: 515.2599.

Trivalent amide 27

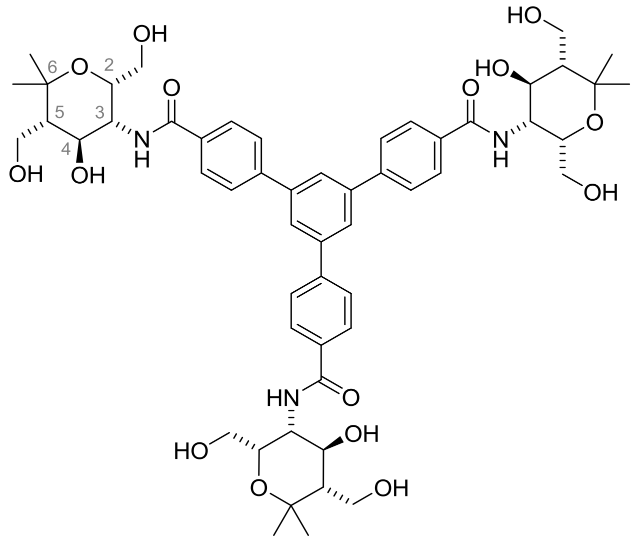

27

According to **GP-2**, aminopyran **1** (50 mg, 0.24 mmol), acid **26** (27 mg, 61 µmol) and HATU (93 mg, 0.24 mmol) were dissolved in DMF (2.0 mL). After addition of Et_3N (0.6 mL, 4.4 mmol), the reaction mixture was stirred at rt for 24 h. The solvent was removed in vacuo and the crude product was purified by flash column chromatography (silica gel, CH_2Cl_2/MeOH 6:1 to 4:1) affording **27** (49 mg, 80%) as a colorless solid.

m.p. 245 °C; $[\alpha]_D^{22}$ = +150.5 (c = 0.74, MeOH); ^{1}H NMR (700 MHz, CD_3OD): δ = 1.34, 1.47 (2 s, 9 H each, CH_3), 1.80 (td, J = 4.8, 10.0 Hz, 3 H, 5-H), 3.56, 3.61 (AB part of ABX system, J_{AB} = 11.7 Hz, J_{AX} = 4.6 Hz, J_{BX} = 6.6 Hz, 3 H each, 2-CH_2), 3.71, 4.00 (2 dd, J = 4.8, 11.1 Hz, 3 H each, 5-CH_2), 4.02 – 4.05 (m, 3 H, 4-H), 4.20 – 4.25 (m, 6 H, 2-H, 3-H), 7.76 (d, J = 8.3 Hz, 6 H, Ar), 7.82 (s, 3 H, Ar), 7.94 (d, J = 8.3 Hz, 6 H, Ar) ppm; ^{13}C NMR (175 MHz, CD_3OD): δ = 26.3, 27.7 (2 q, CH_3), 49.9 (d, C-5), 54.2 (d, C-2), 63.2 (t, 2-CH_2), 63.3 (t, 5-CH_2), 70.9 (d, C-3), 74.1 (d, C-4), 75.9 (s, C-6), 126.6 , 128.4, 129.2 (3 d, Ar), 134.6, 142.8, 145.0 (3 s, Ar), 170.1 (s, C=O) ppm; IR (ATR): $\tilde{\nu}$ = 3320 (O-H, N-H), 2970-2920 (C-H), 1630 (C=O), 1440 (C=C), 1230 (C-O),

1085 (C-O-C) cm^{-1}; HRMS (ESI-TOF): m/z calcd. for $C_{54}H_{69}N_3NaO_{15}$ $[M + Na]^+$: 1022.4615; found: 1022.4612.

Trivalent amide 29

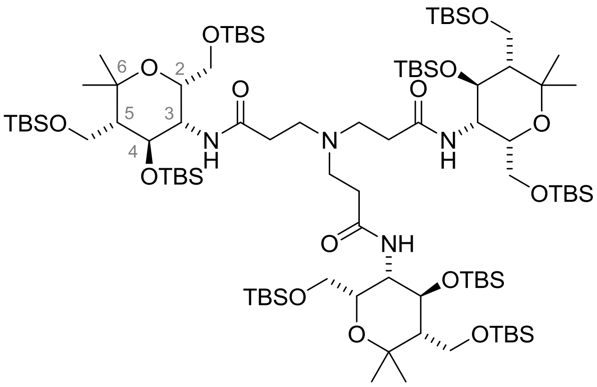

29

According to **GP-2**, aminopyran **3** (87 mg, 159 µmol), 3,3',3''-nitrilotripropanoic acid **28** (9 mg, 39 µmol) and HATU (60 mg, 159 µmol) were dissolved in DMF (2.8 mL). After addition of Et_3N (0.1 mL, 0.7 mmol), the reaction mixture was stirred at rt for 24 h. Saturated $NaHCO_3$ solution (10 mL) was added and the aqueous layer was extracted with EtOAc (3 × 20 mL). The combined organic phases were washed with saturated NaCl solution, dried with Na_2SO_4 and the solvent was removed in vacuo. The crude product was purified by flash column chromatography (silica gel, hexanes, hexanes/EtOAc 10:1 to 6:1) affording **29** (60 mg, 83%) as a colorless oil.

$[\alpha]_D^{22}$ = + 10.8 (c = 2.56, $CHCl_3$); ^1H NMR (700 MHz, $CDCl_3$): δ = 0.01 – 0.03 (m, 36 H, CH_3), 0.09 (s, 9 H, CH_3), 0.16 (s, 9 H, CH_3), 0.85 – 0.88 (m, 54 H, *t*-Bu), 0.89 (s, 27 H, *t*-Bu), 1.17, 1.47 (2 s, 9 H each, CH_3), 1.61 (m_c, 3 H, 5-H), 2.32 (t, J = 7.4 Hz, 6 H, CH_2CO), 2.73 (td, J = 7.4, 14.1 Hz, 3 H, NCH_2), 2.82 (td, J = 7.4, 14.1 Hz, 3 H, NCH_2), 3.45 (dd, J = 8.0, 10.2 Hz, 3 H, 5-CH_2), 3.52, 3.57 (AB part of ABX system, J_{AB} = 10.5 Hz, J_{AB} = J_{BX} = 6.1 Hz, 3 H each, 2-CH_2), 3.70 – 3.76 (m, 6 H, 5-CH_2, 3-H), 4.16 (dt, J = 1.7, 6.1 Hz, 3 H, 2-H), 4.22 (m_c, 3 H, 4-H), 6.30 (d, J = 8.5 Hz, 3 H, NH)

ppm; ^{13}C NMR (175 MHz, $CDCl_3$): δ = -5.14, -5.11, -5.09, -5.00, -4.97, -4.65 (6 q, CH_3), 18.0, 18.2, 18.3 [3 s, Si*C*(CH_3)$_3$], 25.9, 26.0, 26.0 [3 q, SiC(*C*H_3)$_3$], 27.4 , 28.0 (2 q, CH_3), 33.3 (t, CH_2CO), 49.0 (t, NCH_2), 49.5 (d, C-5), 50.2 (d, C-3), 62.6 (t, 5-CH_2), 63.3 (t, 2-CH_2), 67.7 (d, C-2), 68.7 (d, C-4), 74.3 (s, C-6), 171.0 (s, C=O) ppm. IR (ATR): $\tilde{\nu}$ = 3440 (N-H), 2960-2855 (C-H), 1660, 1505 (C=O), 1245 (C-O-C) cm^{-1}; HRMS (ESI-TOF): m/z cald. for $C_{90}H_{193}N_4O_{15}Si_9$ [M + H]$^+$: 1822.2387; found: 1822.2400; calcd. for $C_{90}H_{192}N_4NaO_{15}Si_9$ [M + Na]$^+$: 1844.2206; found: 1844.2203; Elemental analysis calcd. (%) $C_{90}H_{192}N_4O_{15}Si_9$ (1823.3): C 59.29, H 10.61, N 3.07; found: C 59.74, H 10.49, N 2.97.

Sulfated divalent amide 31

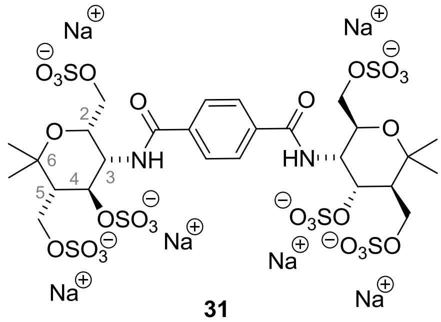

31

According to **GP-5**, polyol **13** (15 mg, 0.028 mmol), SO_3·DMF (97%, 77 mg, 0.50 mmol) and DMF-d_7 (0.6 mL) were stirred overnight. According to method A, 1 M NaOH was added dropwise until pH 10 was reached. The solvents were removed in vacuo and the crude product was purified twice by dialysis (tube width: 10–16 mm, molecular weight cut off: 100–500 Da) affording **31** (19 mg, 60%) as a colorless solid. m.p. 275 °C (decomposition); $[\alpha]_D^{22}$ = +33.7 (c = 0.08, H_2O); ^1H NMR (700 MHz, D_2O): δ = 1.36, 1.52 (2 s, 6 H each, CH_3), 2.53 (td, $J \approx 7.6$, 14.3 Hz, 2 H, 5-H), 4.09, 4.19 (AB part of ABX system, J_{AB} = 10.9 Hz, J_{AX} = 4.3 Hz, J_{BX} = 7.5 Hz, 2 H each, 2-CH_2), 4.25, 4.32 (AB part of ABX system, J_{AB} = 10.5 Hz, J_{AX} = 6.3 Hz, J_{BX} = 7.9 Hz, 2 H

each, 5-CH_2), 4.53 – 4.57 (m, 2 H, 3-H), 4.57 – 4.62 (m, 2 H, 2-H), 4.69 – 4.76 (m, 2 H, 4-H), 7.88 (s, 4 H, Ar) ppm; ^{13}C NMR (175 MHz, D_2O): δ = 24.8, 26.4 (2 q, CH_3), 44.2 (d, C-5), 52.3 (d, C-2), 67.3 (t, 2-CH_2), 67.6 (t, 5-CH_2), 67.7 (d, C-3), 76.2 (d, C-4), 76.6 (s, C-6), 128.7 (d, Ar) ppm; signals for C=O and Ar singlet could not be detected; IR (ATR): $\tilde{\nu}$ = 3480 (N-H), 2985 (C-H), 1535 (C=C), 1635 (C=O), 1255 (C-O), 1130 ($SO_3^-Na^+$) cm^{-1}; HRMS (ESI-TOF): m/z calcd. for $C_{26}H_{34}N_2Na_7O_{28}S_6$ [M + Na]$^+$: 1174.8906; found: 1174.8819.

Sulfated divalent amide 32

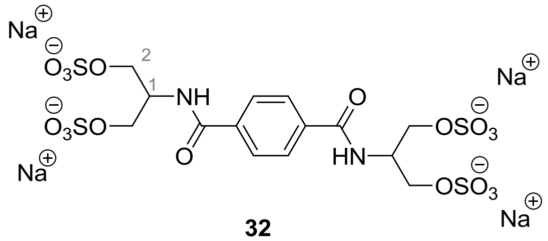

32

According to **GP-5**, polyol **21** (20 mg, 0.064 mmol), SO_3·DMF (118 mg, 0.770 mmol) and DMF-d_7 (0.6 mL) were stirred overnight. After ^1H NMR control, the reaction mixture was stirred for 4 d and each day a new portion of SO_3·DMF (118 mg) was added. According to method A, 1 M NaOH was added dropwise until pH 12 was reached. The solvents were removed in vacuo and the crude product was purified by dialysis (tube width: 10–16 mm, molecular weight cut off: 100–500 Da) affording **32** (32 mg, 69%) as a colorless solid.

m.p. 215 – 217 °C (decomposition); ^1H NMR (500 MHz, D_2O): δ = 4.20, 4.23 (AB part of ABX system, J_{AB} = 9.7 Hz, J_{AX} = 4.1 Hz, J_{BX} = 5.0 Hz, 4 H each, 2-H), 4.58 – 4.63 (m, 2 H, 1-H), 7.81 (s, 4 H, Ar) ppm; ^{13}C NMR (125 MHz, D_2O): δ = 49.0 (d, C-1), 66.7 (t, C-2), 127.9 (d, Ar), 136.9 (s, Ar), 170.6 (s, C=O) ppm; IR (ATR): $\tilde{\nu}$ = 3375 (N-H), 2985 (C-H), 1535 (C=C), 1645 (C=O), 1290 (C-O), 1130 ($SO_3^-Na^+$) cm^{-1}; HRMS (ESI-TOF): m/z calcd. for $C_{14}H_{16}N_2Na_5O_{18}S_4$ [M + Na]$^+$: 742.8758; found: 742.8763

Sulfated divalent amide 33

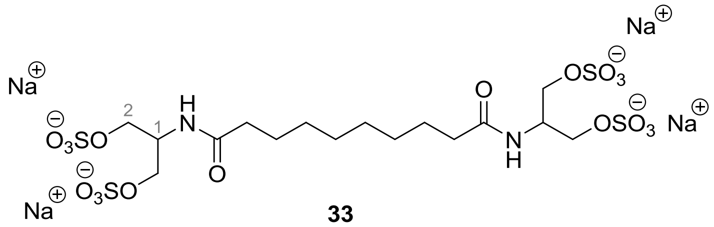

33

According to **GP-5**, polyol **22** (20 mg, 0.057 mmol), SO_3·DMF (106 mg, 0.689 mmol) and DMF-d_7 (0.6 mL) were stirred overnight. According to method A, 1 M NaOH was added dropwise until pH 12 was reached. The solvents were removed in vacuo and the crude product was purified by dialysis (tube width: 10–16 mm, molecular weight cut off: 100–500 Da) affording **33** (34 mg, 79%) as a colorless solid.

Melting range: 200 – 205 °C (decomposition); ^{1}H NMR (500 MHz, D_2O): δ = 1.36 (bs, 8 H, CH_2), 1.61 – 1.69 (m, 4 H, CH_2), 2.34 (t, J = 7.4 Hz, 4 H, CH_2), 4.17, 4.20 (AB part of ABX system, J_{AB} = 9.0 Hz, J_{AX} = 3.6 Hz, J_{BX} = 4.3 Hz, 4 H each, 2-H), 4.47 (m_c, 2 H, 1-H) ppm; ^{13}C NMR (125 MHz, D_2O): δ = 25.6, 28.4, 28.5, 36.1 (4 t, CH_2), 48.2 (d, C-1), 66.9 (t, C-2), 177.8 (s, C=O) ppm; IR (ATR): $\tilde{\nu}$ = 3385 (N-H), 2980 (C-H), 1640 (C=O), 1255 (C-O), 1130 ($SO_3^-Na^+$) cm^{-1} HRMS (ESI-TOF): m/z calcd. for $C_{16}H_{28}N_2Na_5O_{18}S_4$ $[M + Na]^+$: 778.9703; found: 778.9682.

Sulfated trivalent amide 34

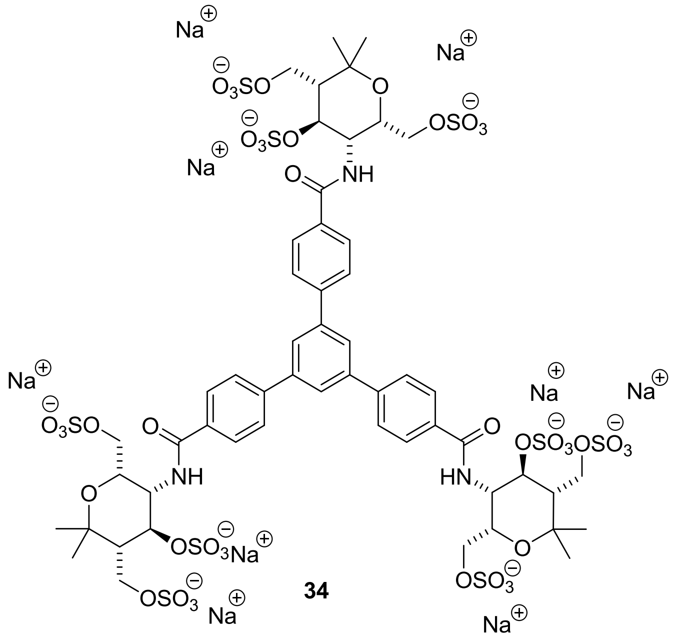

According to **GP-5**, polyol **27** (32 mg, 0.032 mmol), SO_3·DMF (97%, 136 mg, 0.864 mmol) and DMF-d_7 (0.7 mL) were stirred overnight. After ^{1}H NMR control, the reaction mixture was stirred for 2 d and each day a new portion of SO_3·DMF (136 mg) was added. According to method B, NaOH 0.5 M was added dropwise until pH 9 was reached. The reaction mixture was filtrated through an ion exchange DOWEX® Na^+ column. The solvents were removed in vacuo and the crude product was purified by dialysis (tube width: 10–16 mm, molecular weight cut off: 500–1000 Da). The final product was filtrated through a syringe filter affording **34** (51 mg, 84%) as a colorless solid.

m.p. 260 °C (decomposition); $[\alpha]_D^{22}$ = +23.4 (c = 0.5, H_2O); ^{1}H NMR (700 MHz, D_2O): δ = 1.41, 1.55 (2 s, 9 H each, CH_3), 2.56 (m_c, 3 H, 5-H), 4.14 (A part of ABX system, J_{AB} = 10.2 Hz, J_{AX} = 7.8 Hz, 3 H, 2-CH_2), 4.23 – 4.26 (m, 3 H, 2-CH_2), 4.27 – 4.30 (m, 3 H, 5-CH_2), 4.36 – 4.38 (m, 3 H, 5-CH_2), 4.61 (m_c, 3 H, 2-H), 4.62 – 4.66 (m, 3 H, 3-H), 8.00 (s, 12 H, Ar), 8.17 (s, 3 H, Ar) ppm; the signal of 4-H could not be detected (overlapping with D_2O peak ≈ 4.77 ppm); ^{13}C NMR (175 MHz, D_2O): δ = 24.2, 26.0 (2

q, CH_3), 43.9 (d, C-5), 52.0 (d, C-2), 67.1 (t, 5-CH_2), 67.4 (t, 2-CH_2), 67.5 (d, C-3), 76.3 (d, C-4), 76.4 (s, C-6), 125.8, 127.4, 128.2 (3 d, Ar), 132.9, 141.3, 143.7 (3 s, Ar), 171.0 (s, C=O) ppm; IR (ATR): $\tilde{\nu}$ = 3460 (N-H), 2960 (C-H), 1535 (C=C), 1635 (C=O), 1220 (C-O), 1130 ($SO_3^-Na^+$) cm^{-1}; HRMS (ESI-TOF): m/z cald. for $C_{54}H_{60}N_3Na_{11}O_{42}S_9$ [M + 2Na]$^{2+}$: 981.9495; found: 981.9458.

^{1}H NMR (500 MHz, $CDCl_3$):

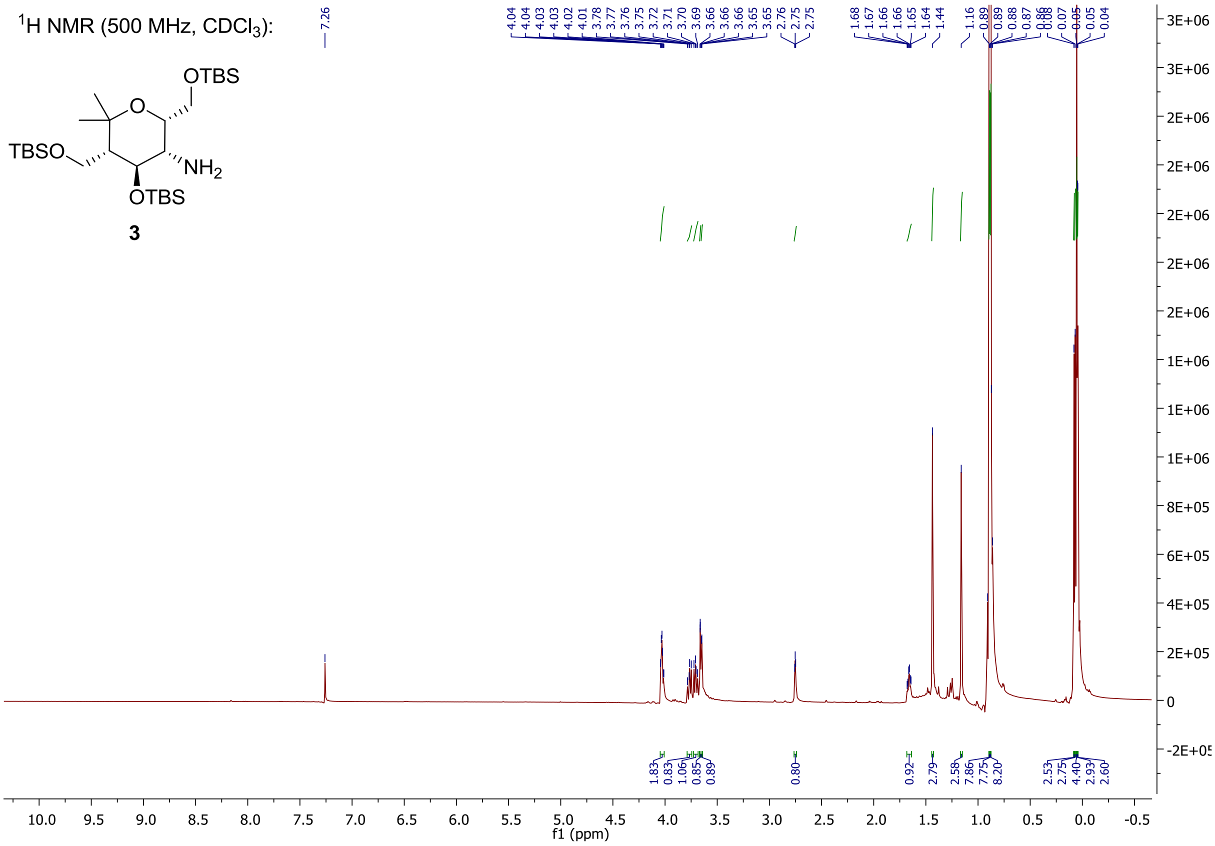

^{13}C NMR (125 MHz, $CDCl_3$):

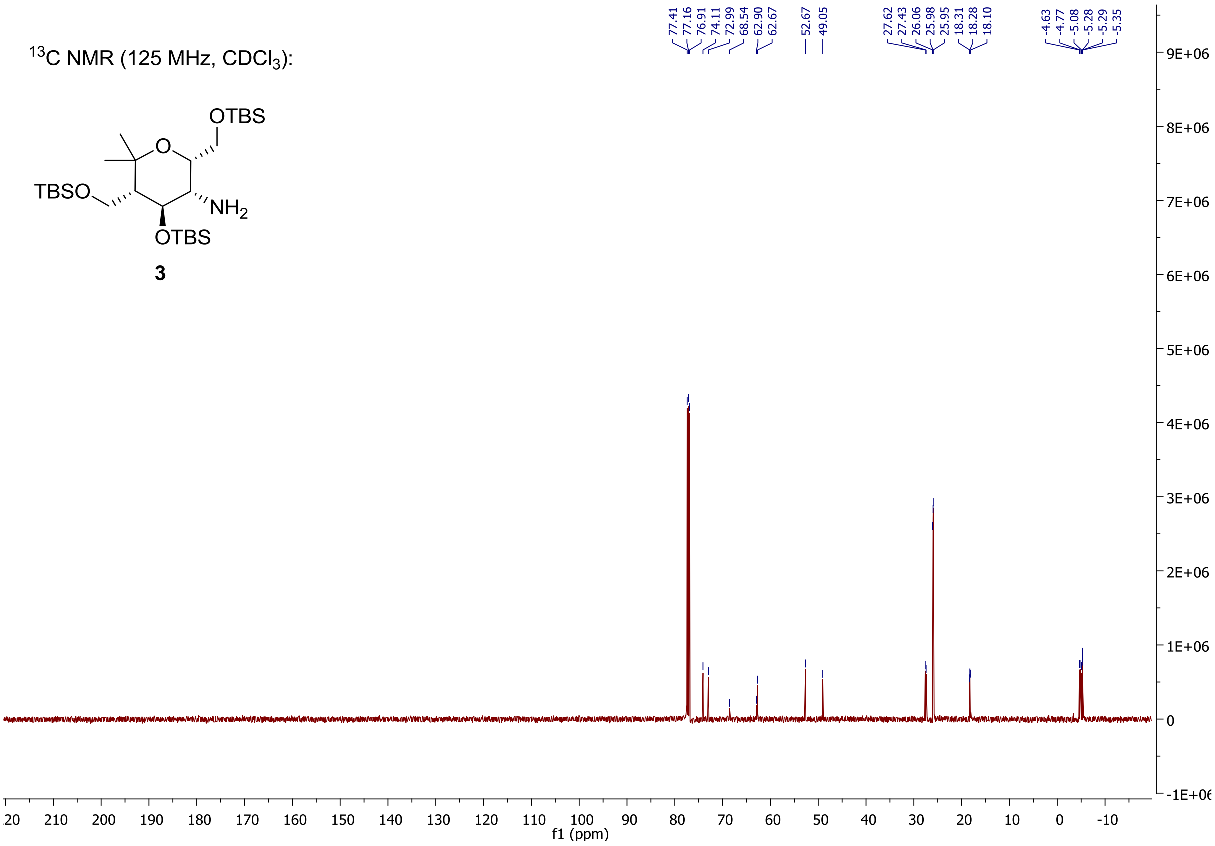

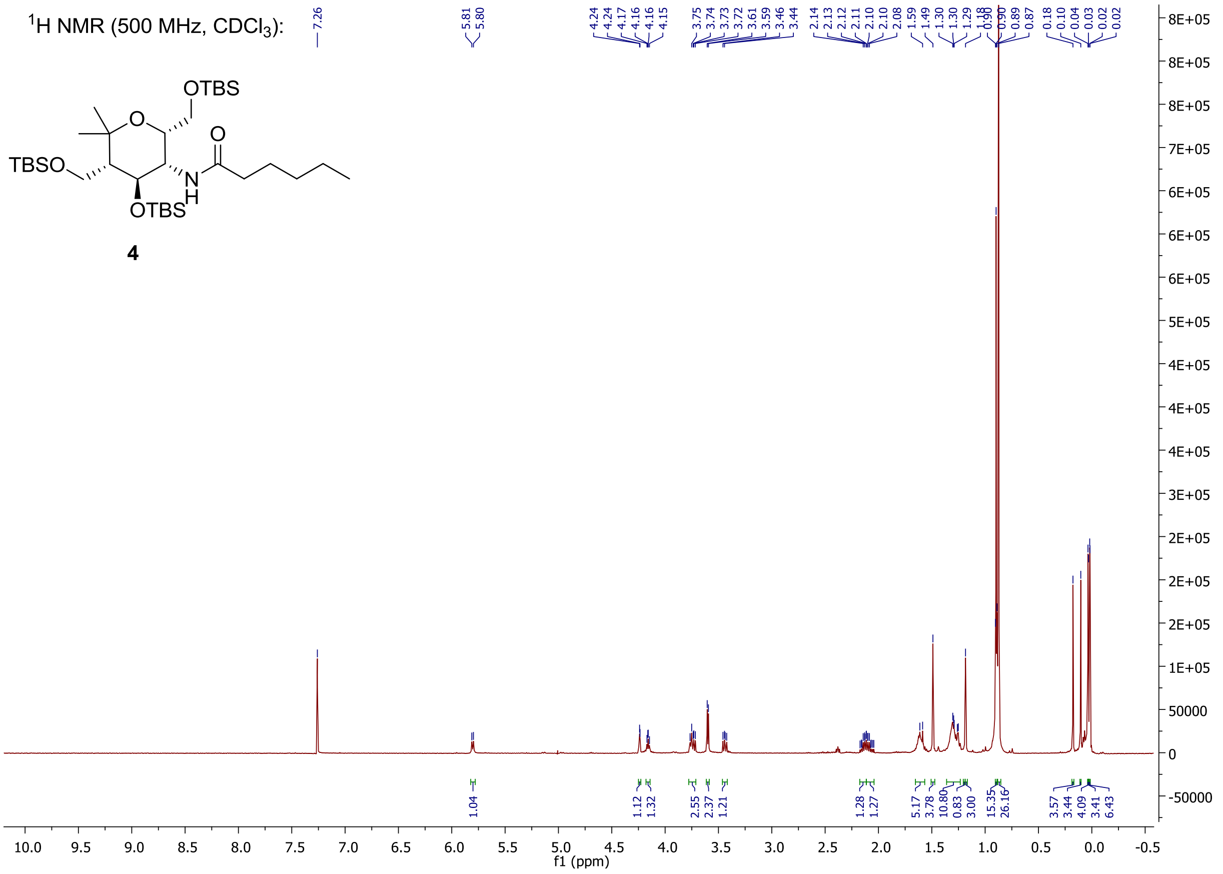

1H NMR (500 MHz, CDCl3):
OTBS
O
TBSO
N
H
OTBS
4
7.26
5.81
5.80
4.24
4.24
4.17
4.16
4.16
4.15
3.75
3.74
3.73
3.72
3.61
3.59
3.46
3.44
2.14
2.13
2.12
2.11
2.10
2.10
2.08
1.59
1.49
1.30
1.30
1.29
1.18
0.90
0.89
0.87
0.18
0.10
0.04
0.03
0.02
0.02
1.04
1.12
1.32
2.55
2.37
1.21
1.28
1.27
5.17
3.78
10.80
0.83
3.00
15.35
26.16
3.57
3.44
4.09
3.41
6.43
f1 (ppm)

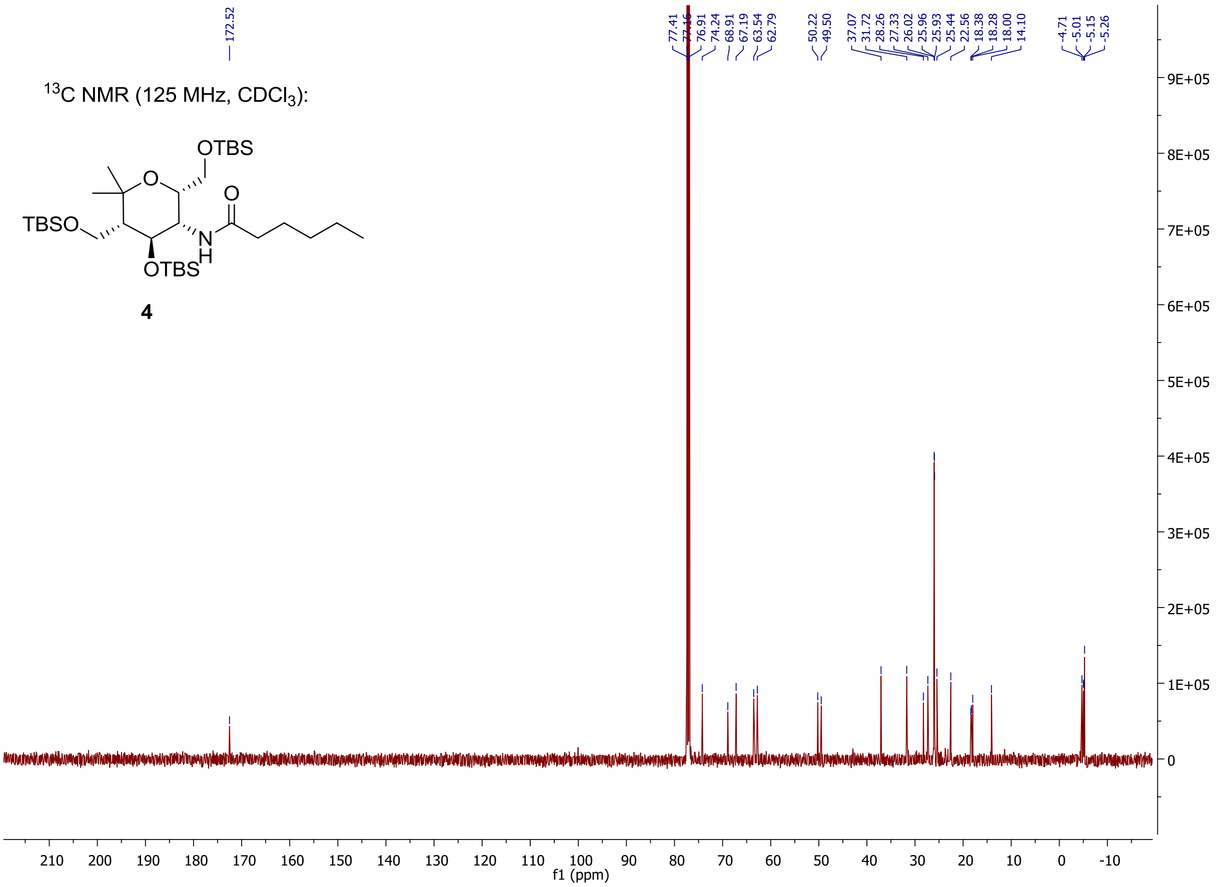

13C NMR (125 MHz, CDCl3):
OTBS
O
TBSO
N
H
OTBS
4
172.52
77.41
77.16
76.91
74.24
68.91
67.19
63.54
62.79
50.22
49.50
37.07
31.72
28.26
27.33
26.02
25.96
25.93
25.44
22.56
18.38
18.28
18.00
14.10
-4.71
-5.01
-5.15
-5.26
f1 (ppm)

^1H NMR (500 MHz, $CDCl_3$):

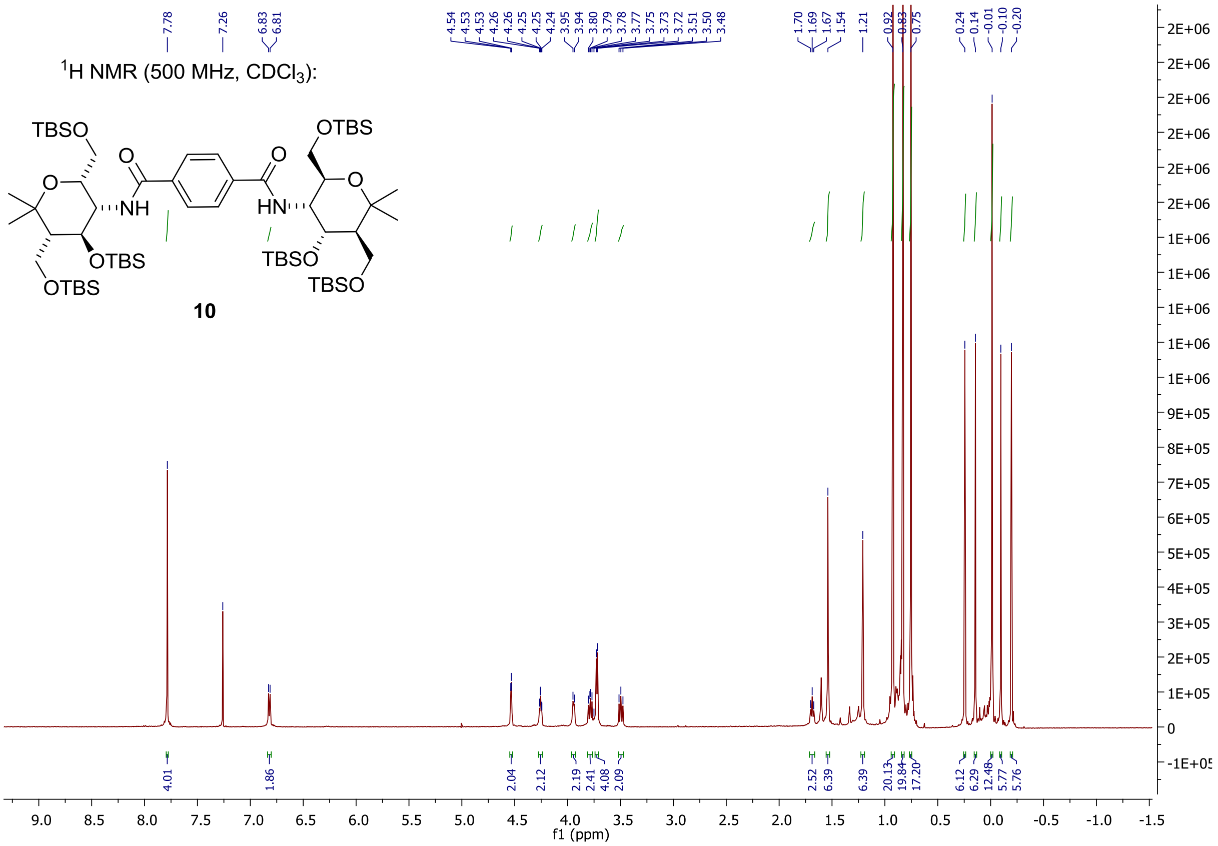

10

^{13}C NMR (125 MHz, $CDCl_3$):

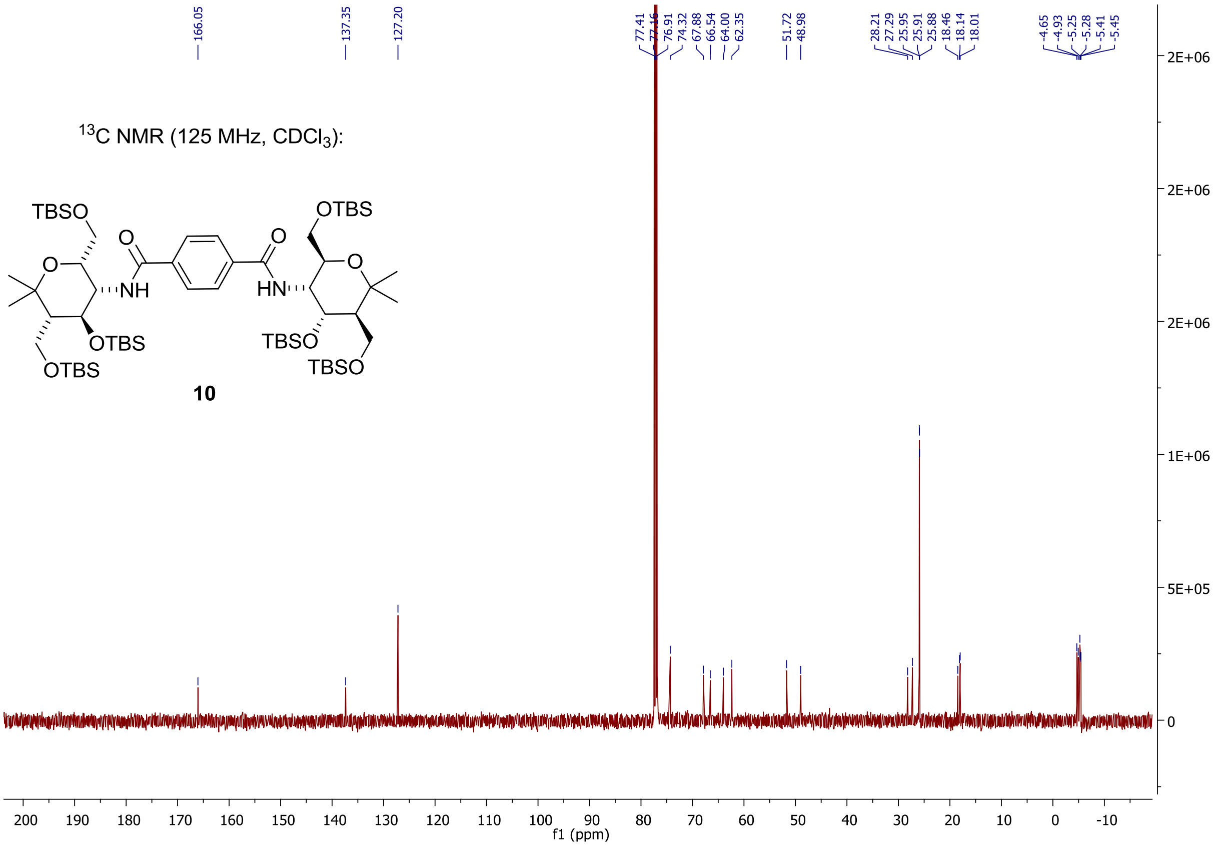

10

^1H NMR (700 MHz, CD_3OD):

13

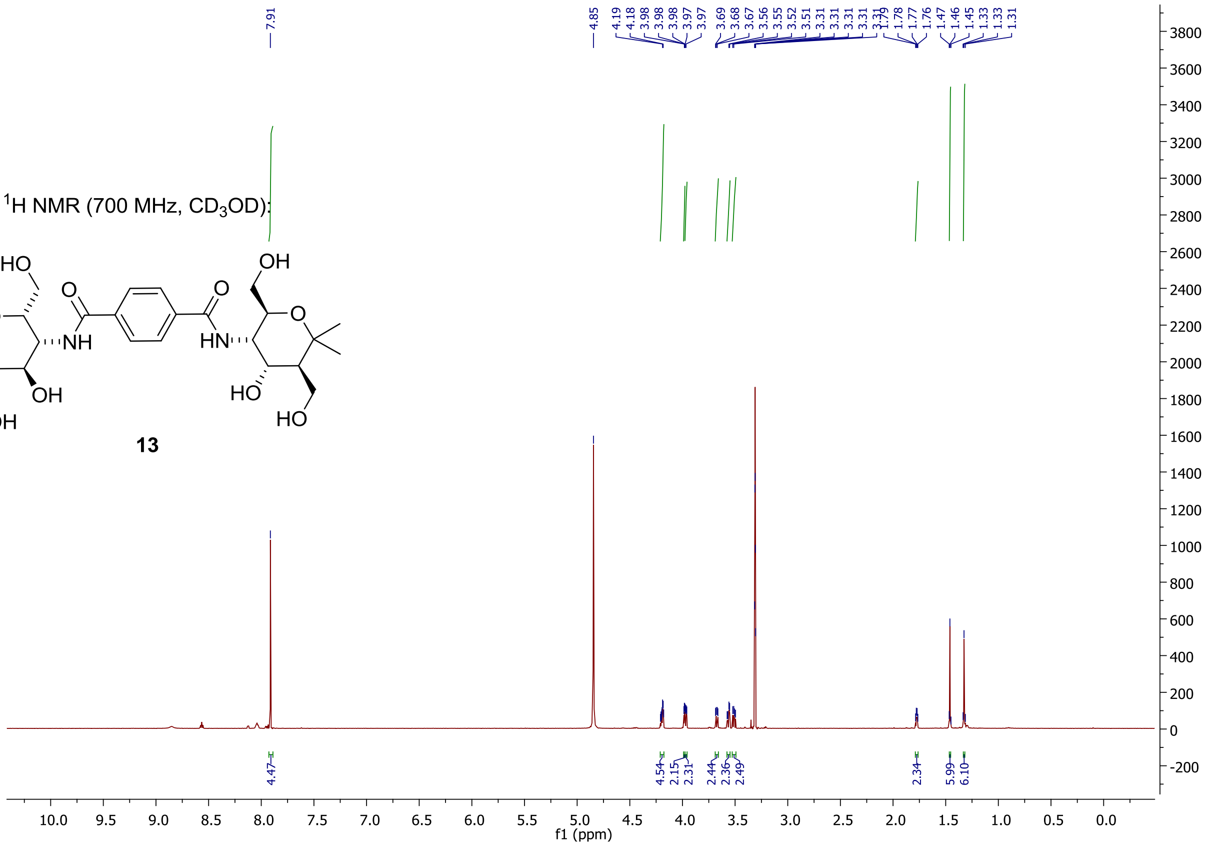

^{13}C NMR (175 MHz, CD_3OD):

13

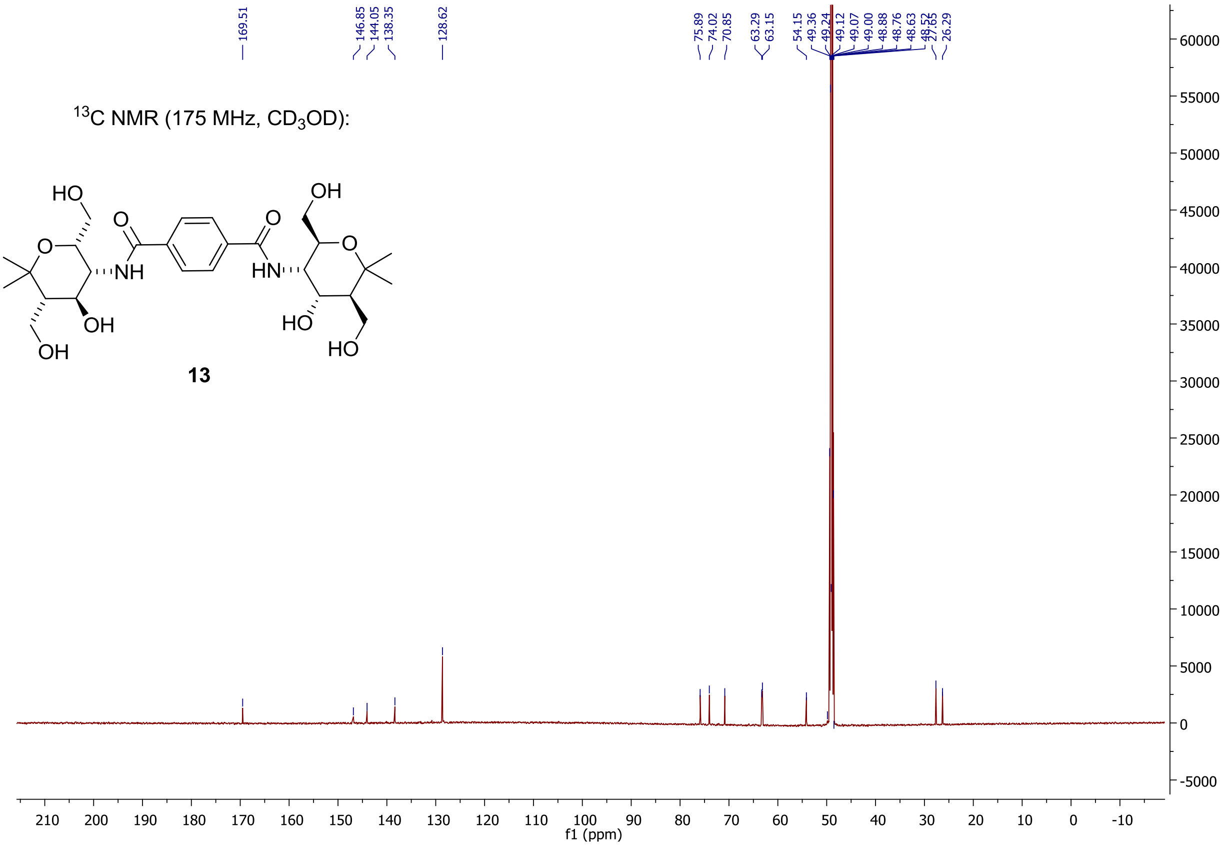

^{1}H NMR (500 MHz, $CDCl_3$):

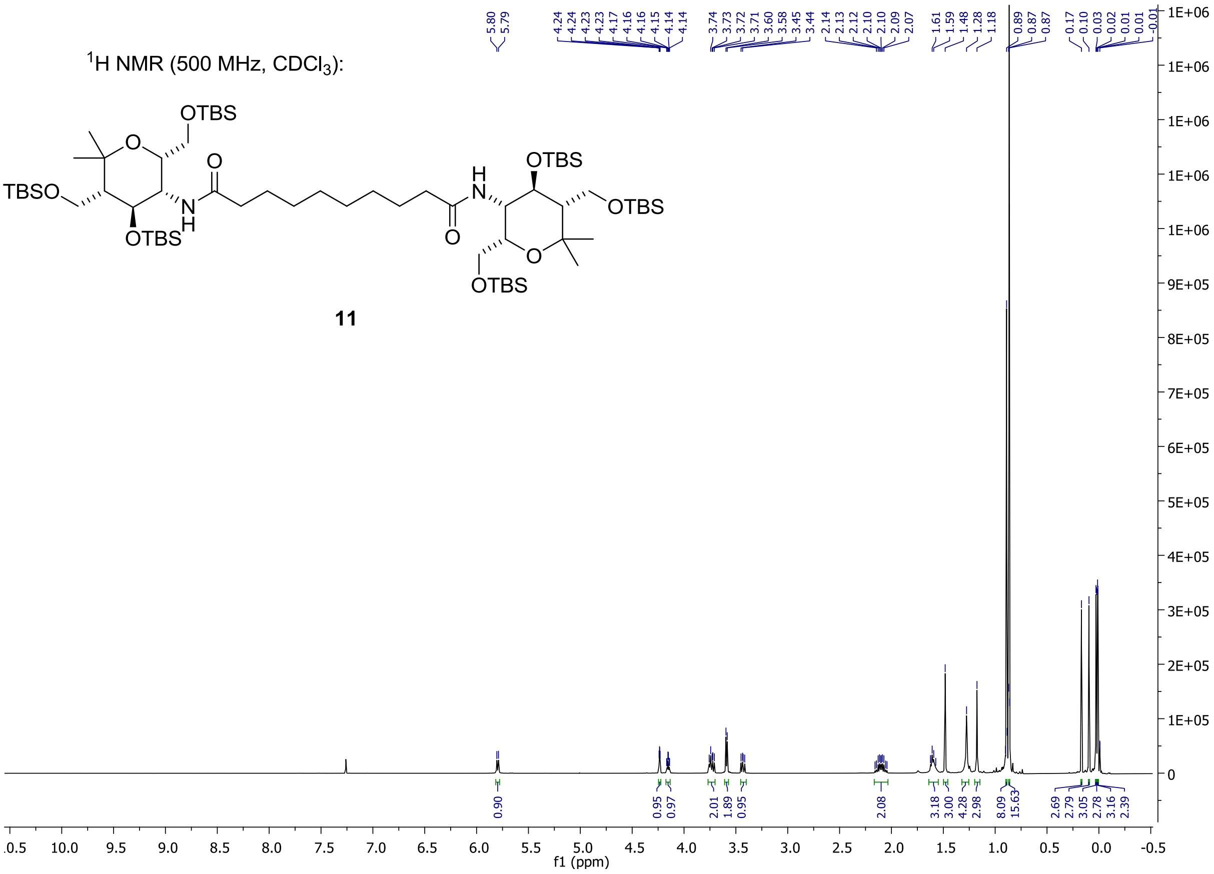

11

^{13}C NMR (125 MHz, $CDCl_3$):

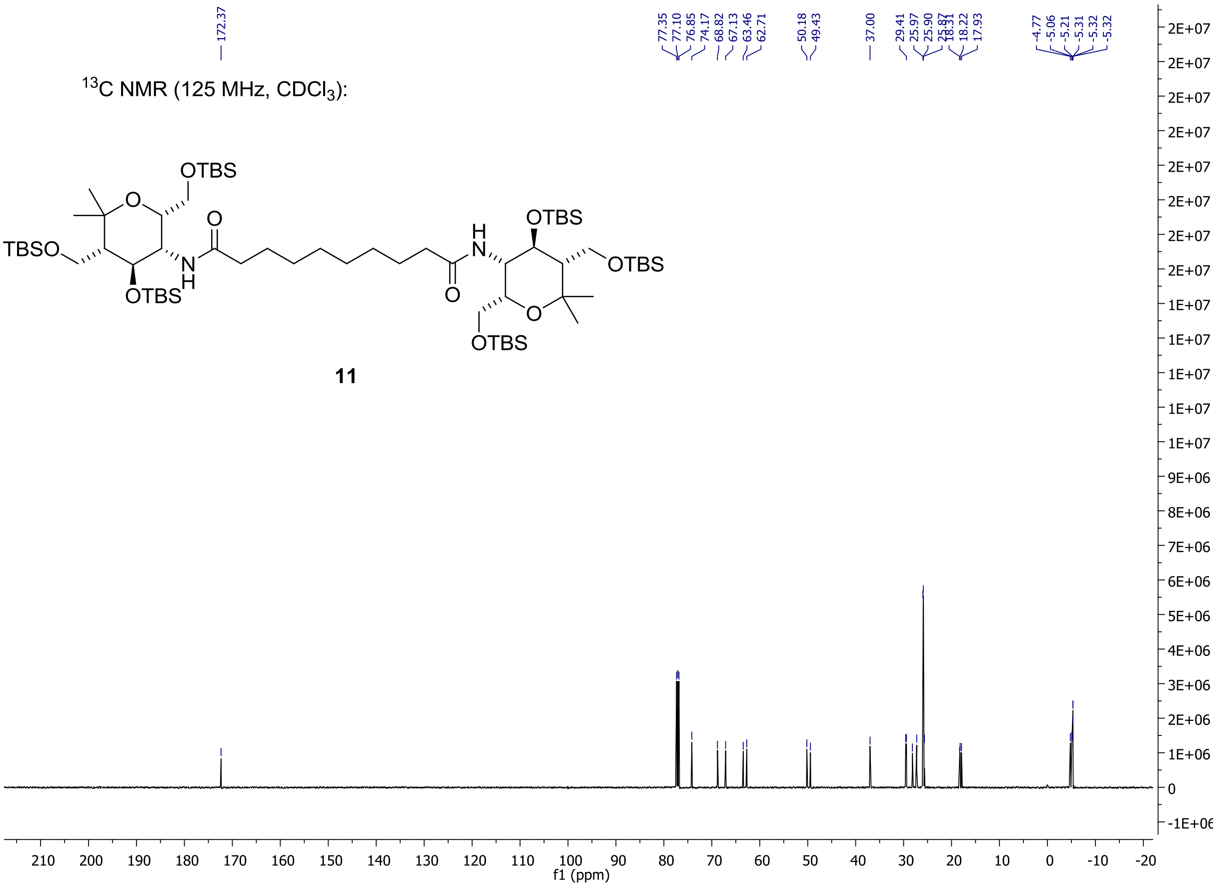

11

^1H NMR (500 MHz, CD_3OD):

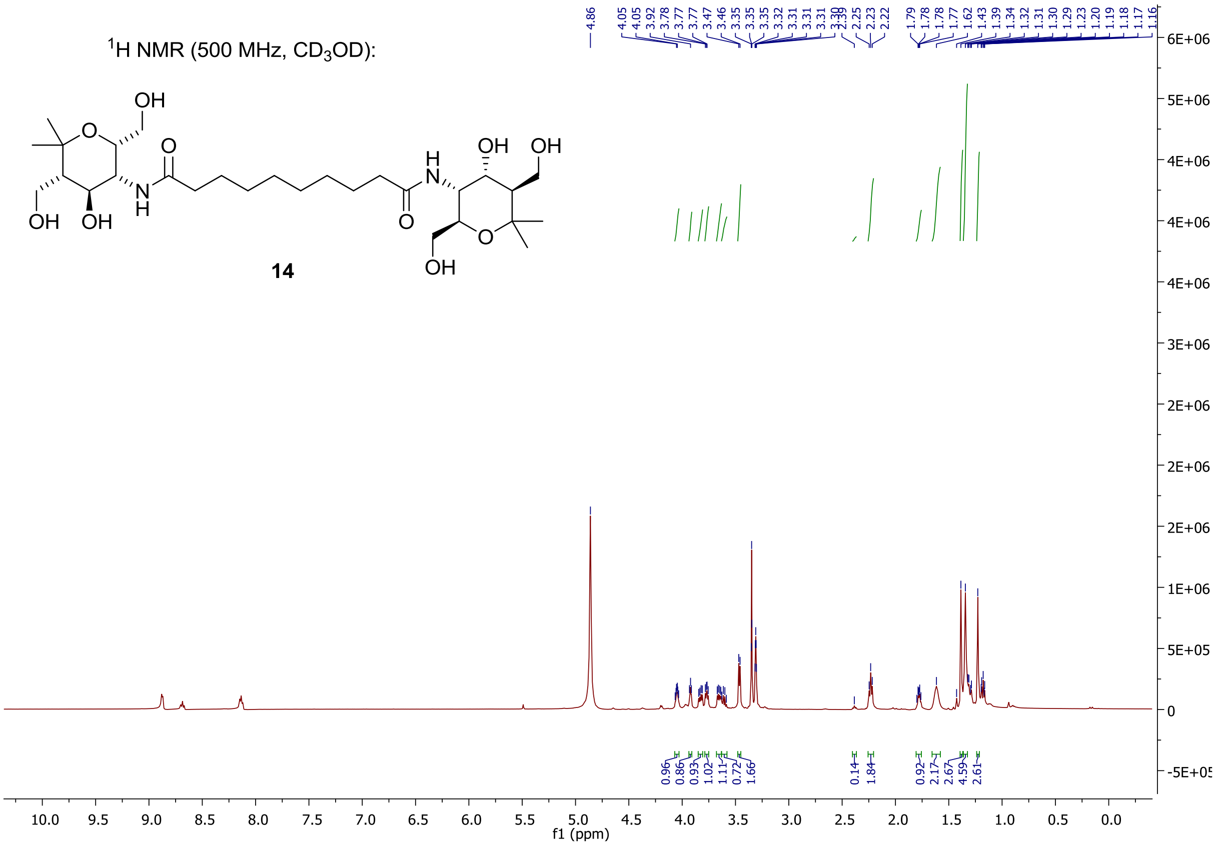

14

^{13}C NMR (125 MHz, CD_3OD):

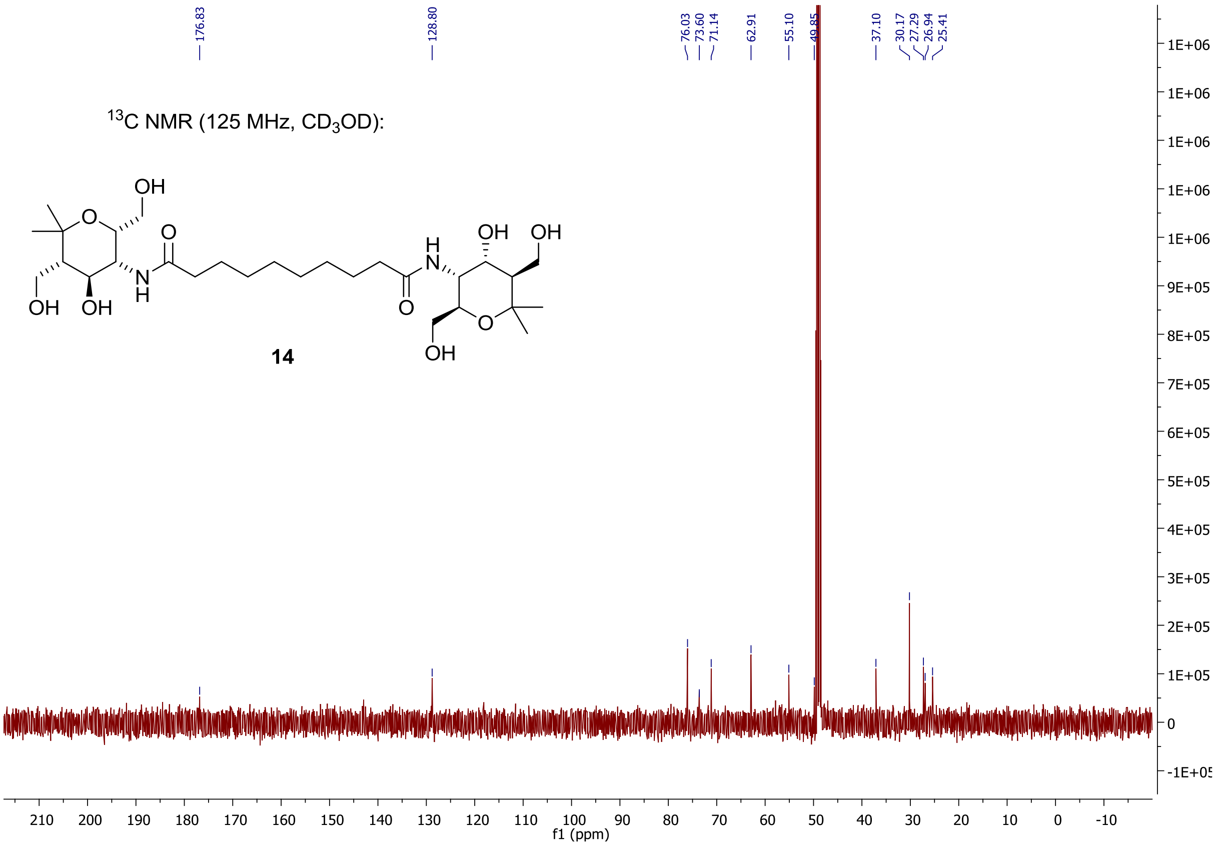

14

^{1}H NMR (500 MHz, $CDCl_3$):

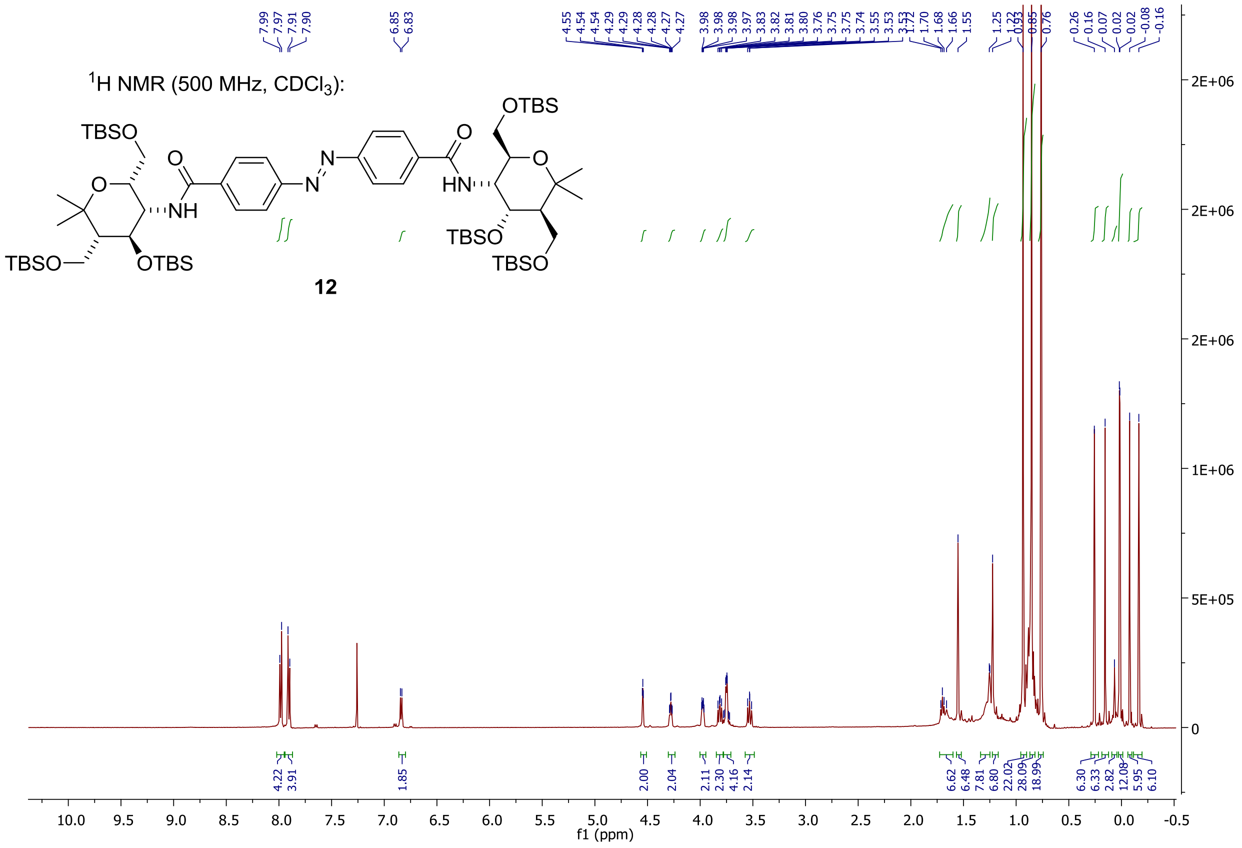

12

^{13}C NMR (125 MHz, $CDCl_3$):

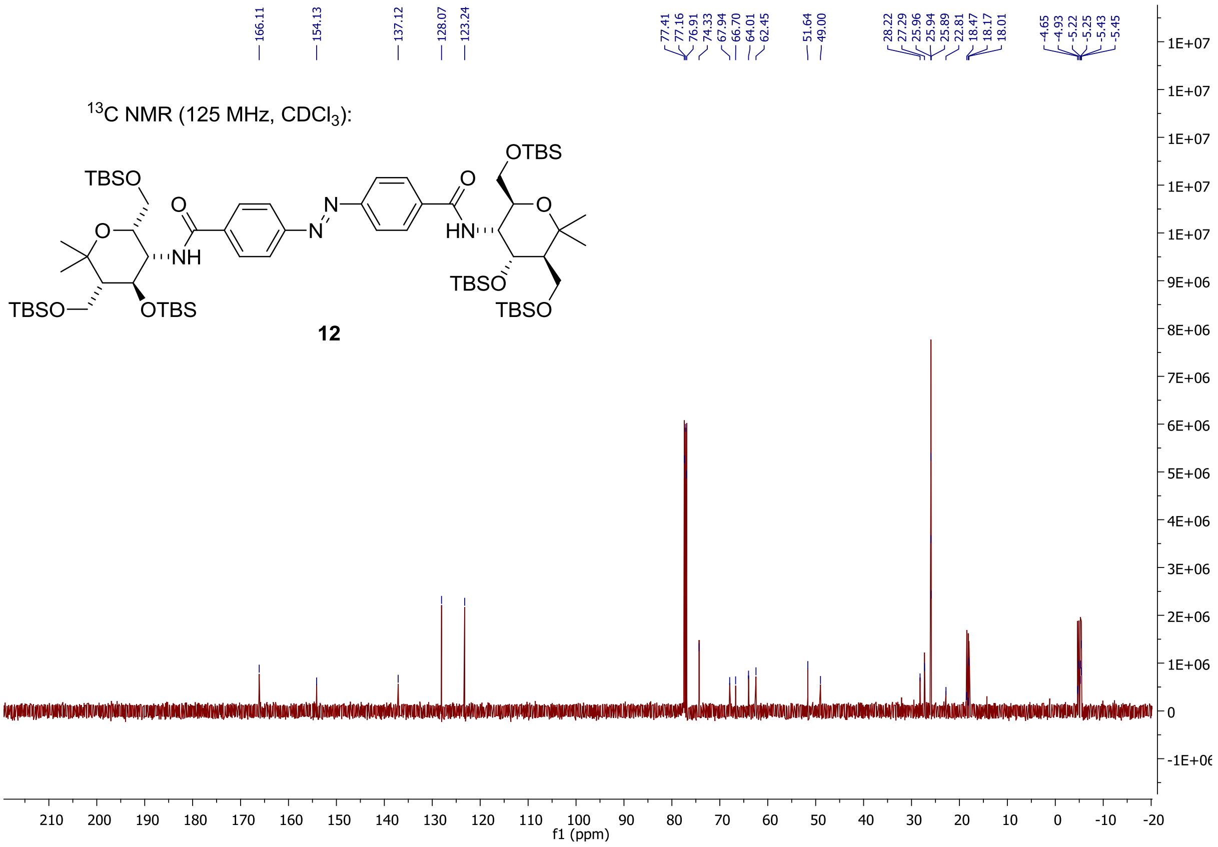

12

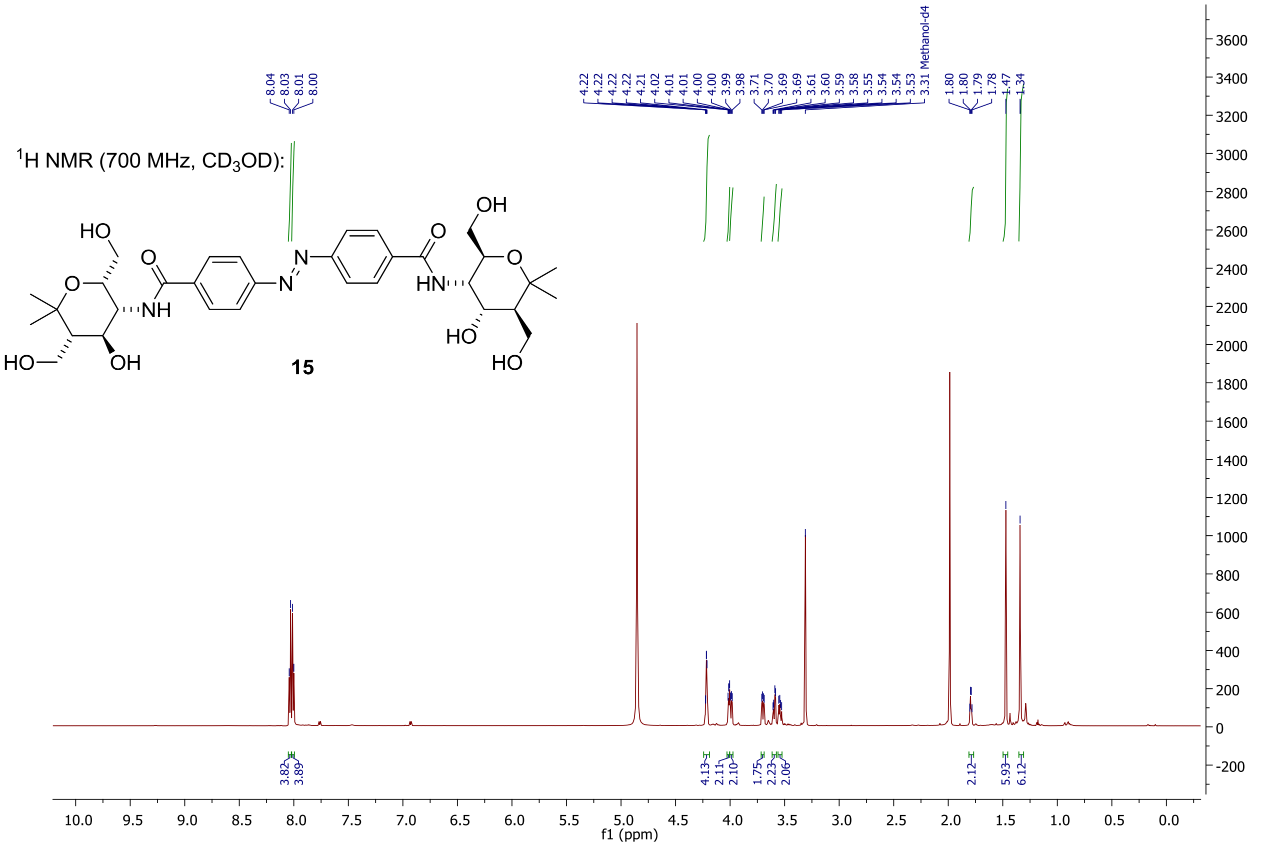

¹H NMR (700 MHz, CD₃OD):
15
8.04 8.03 8.01 8.00
4.22 4.22 4.22 4.22 4.21 4.02 4.01 4.01 4.00 4.00 3.99 3.98 3.71 3.70 3.69 3.69 3.61 3.60 3.59 3.58 3.55 3.54 3.54 3.53 3.31 Methanol-d4
1.80 1.80 1.79 1.78 1.47 1.34
3.82 3.89 4.13 2.11 2.10 1.75 2.23 2.06 2.12 5.93 6.12
f1 (ppm)

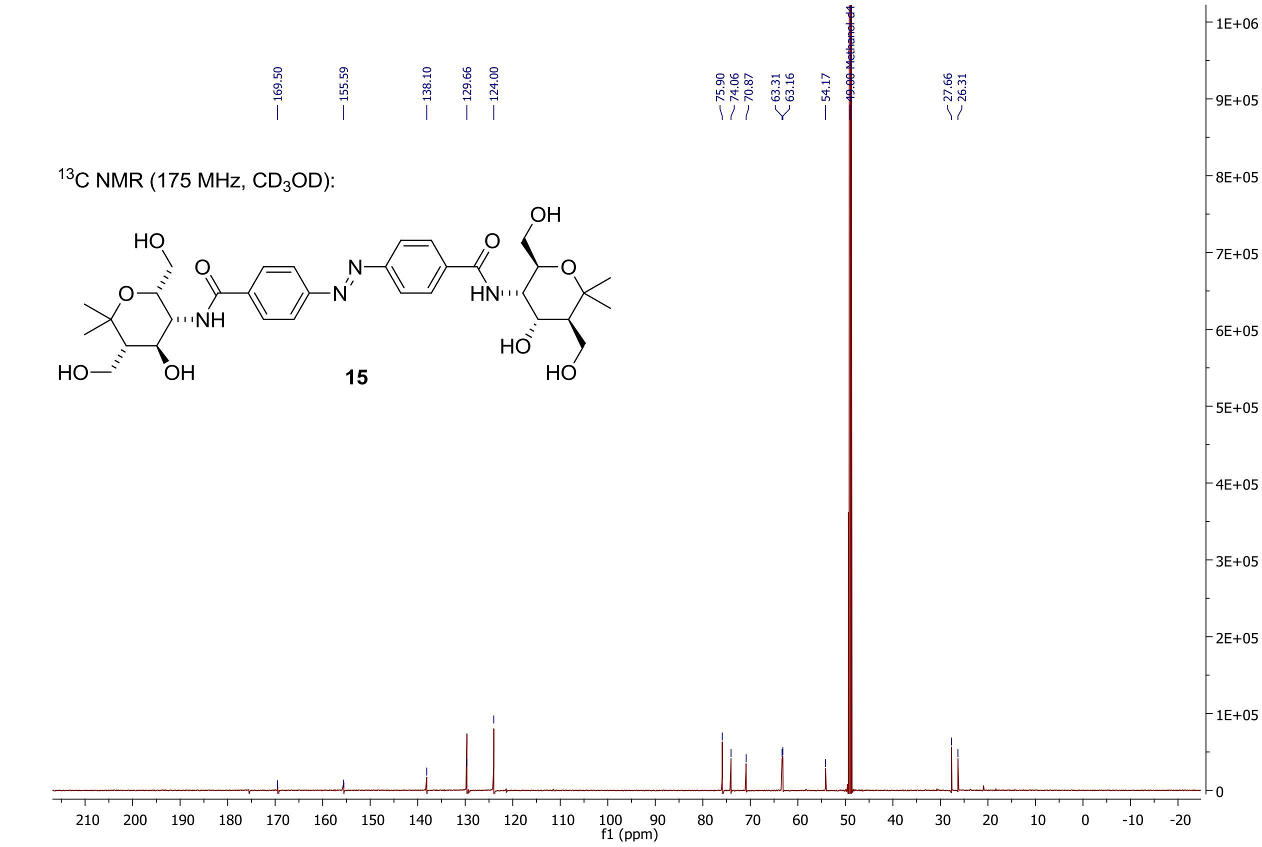

¹³C NMR (175 MHz, CD₃OD):
15
169.50 155.59 138.10 129.66 124.00 75.90 74.06 70.87 63.31 63.16 54.17 49.00 Methanol-d4 27.66 26.31
f1 (ppm)

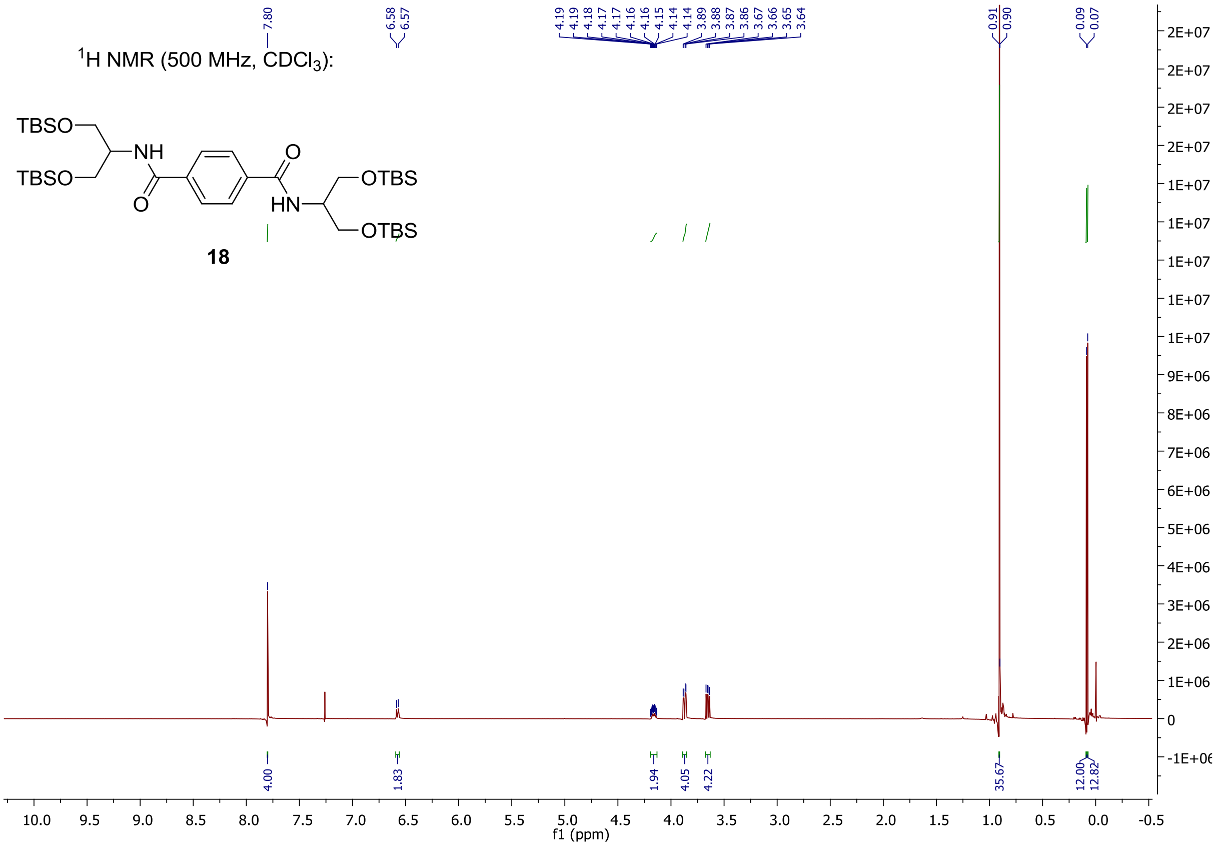

^{1}H NMR (500 MHz, $CDCl_3$):
TBSO
NH
TBSO
O
O
HN
OTBS
OTBS
18
7.80
6.58
6.57
4.19
4.19
4.18
4.17
4.17
4.16
4.15
4.14
4.14
3.89
3.88
3.87
3.86
3.67
3.66
3.65
3.64
0.91
0.90
0.09
0.07
4.00
1.83
1.94
4.05
4.22
35.67
12.00
12.82
f1 (ppm)

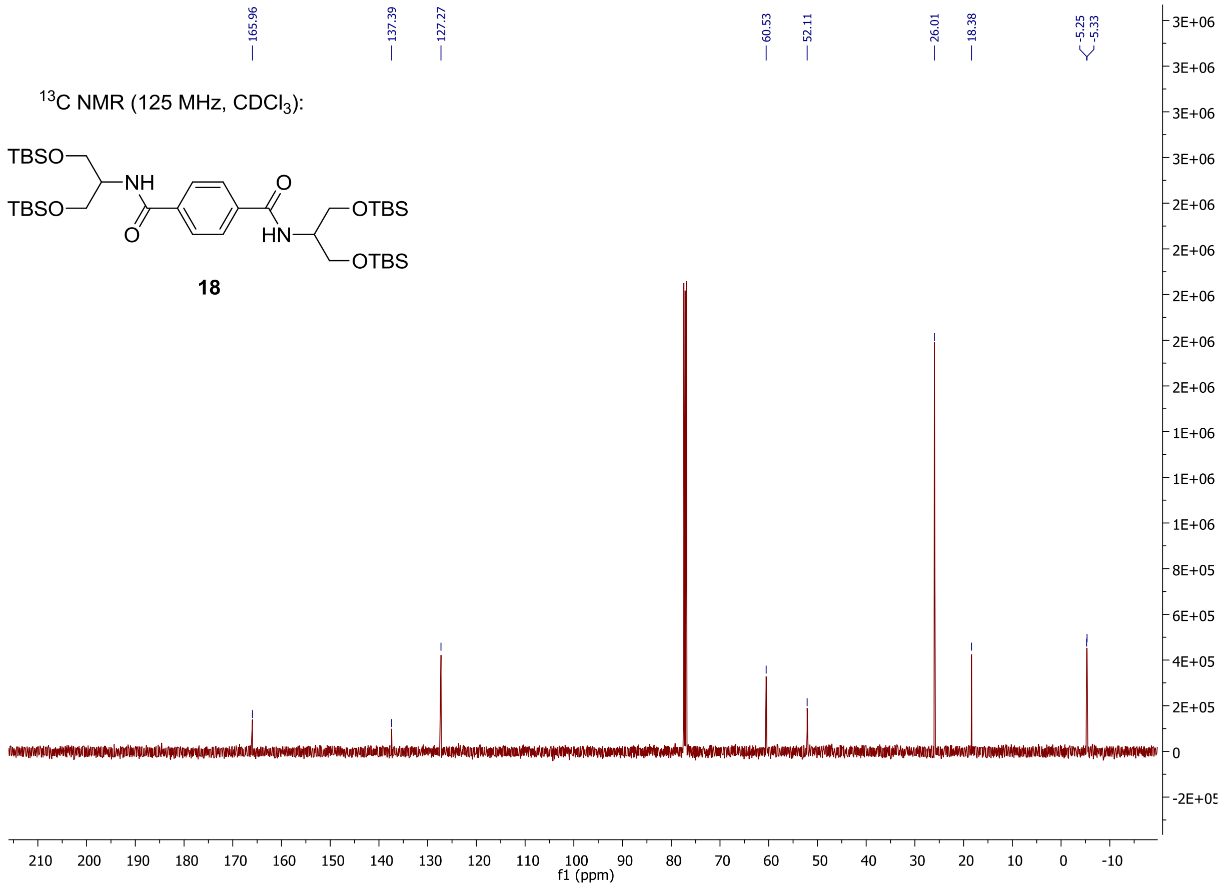

^{13}C NMR (125 MHz, $CDCl_3$):
TBSO
NH
TBSO
O
O
HN
OTBS
OTBS
18
165.96
137.39
127.27
60.53
52.11
26.01
18.38
-5.25
-5.33
f1 (ppm)

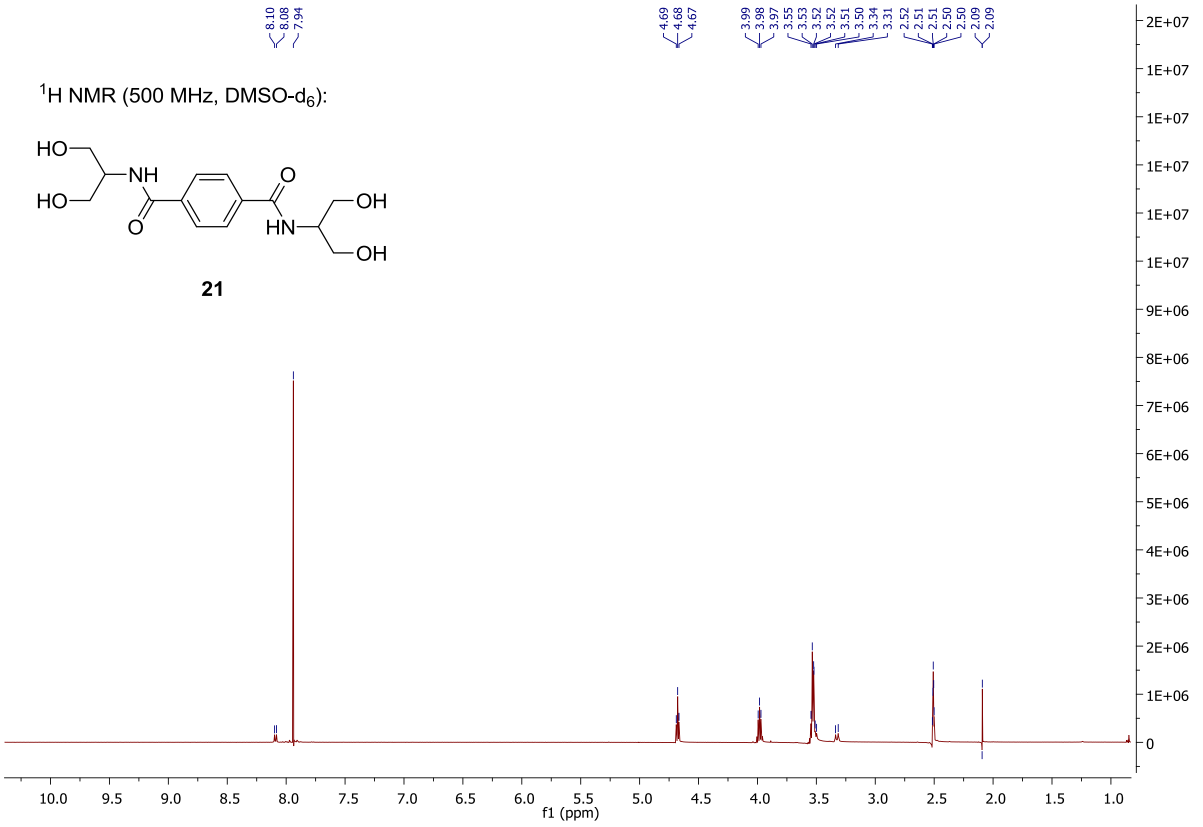

^{1}H NMR (500 MHz, DMSO-d_6):
21
8.10
8.08
7.94
4.69
4.68
4.67
3.99
3.98
3.97
3.55
3.53
3.52
3.52
3.51
3.50
3.34
3.31
2.52
2.51
2.51
2.50
2.50
2.09
2.09
f1 (ppm)

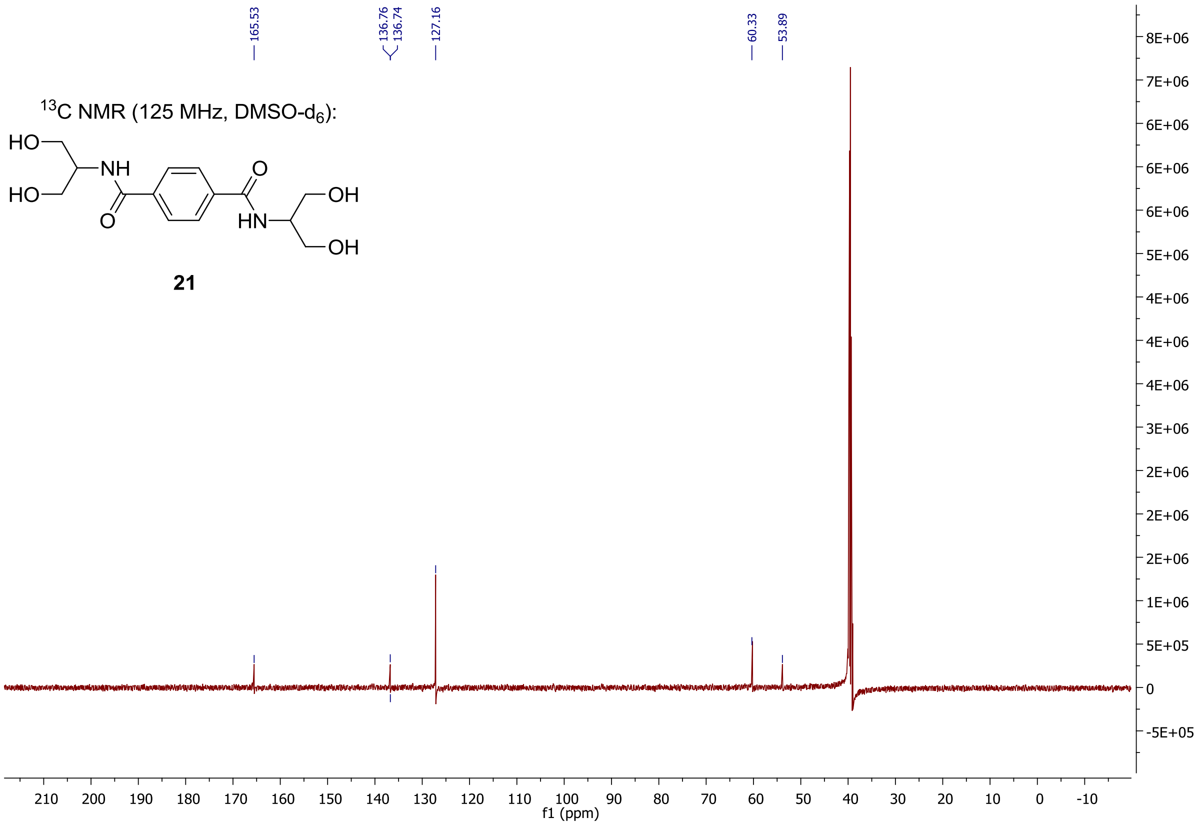

^{13}C NMR (125 MHz, DMSO-d_6):
21
165.53
136.76
136.74
127.16
60.33
53.89
f1 (ppm)

^1H NMR (500 MHz, $CDCl_3$):

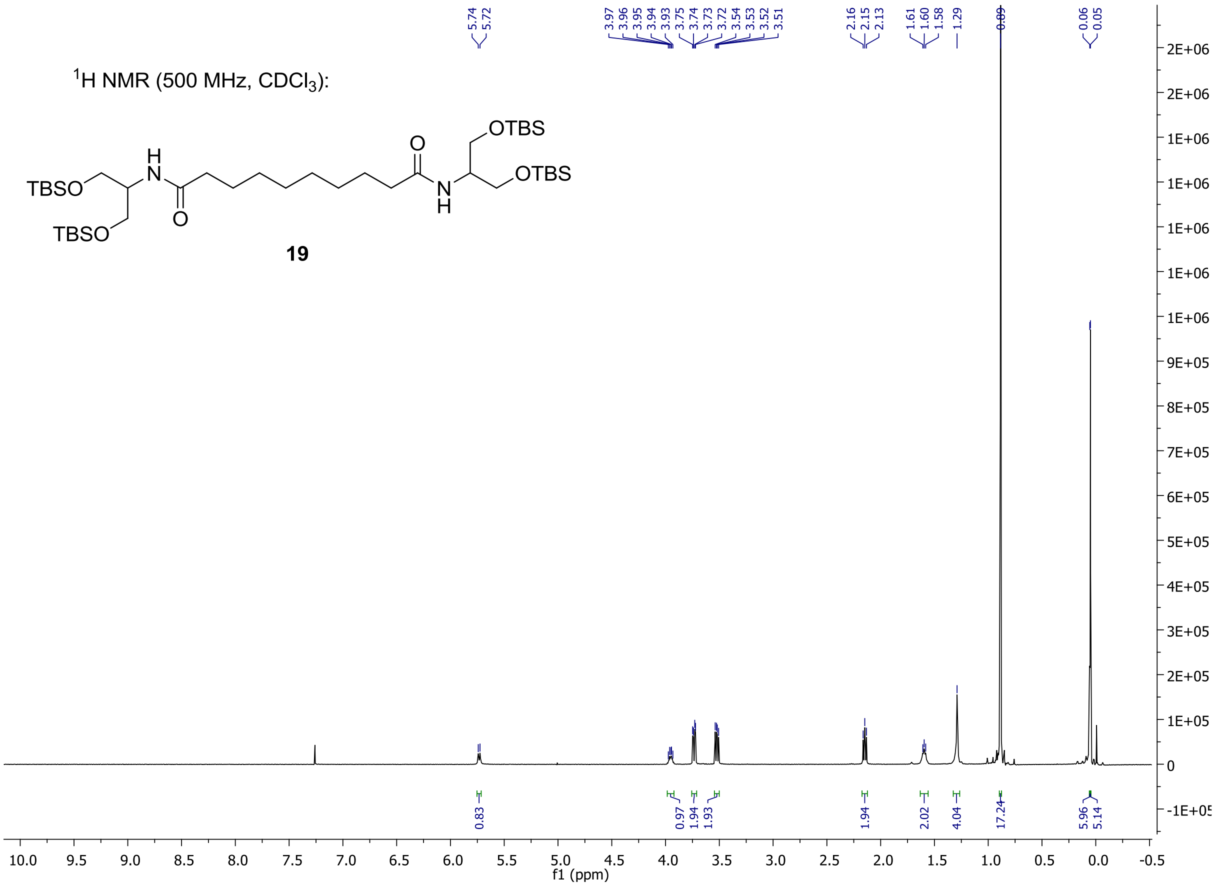

19

^{13}C NMR (125 MHz, $CDCl_3$):

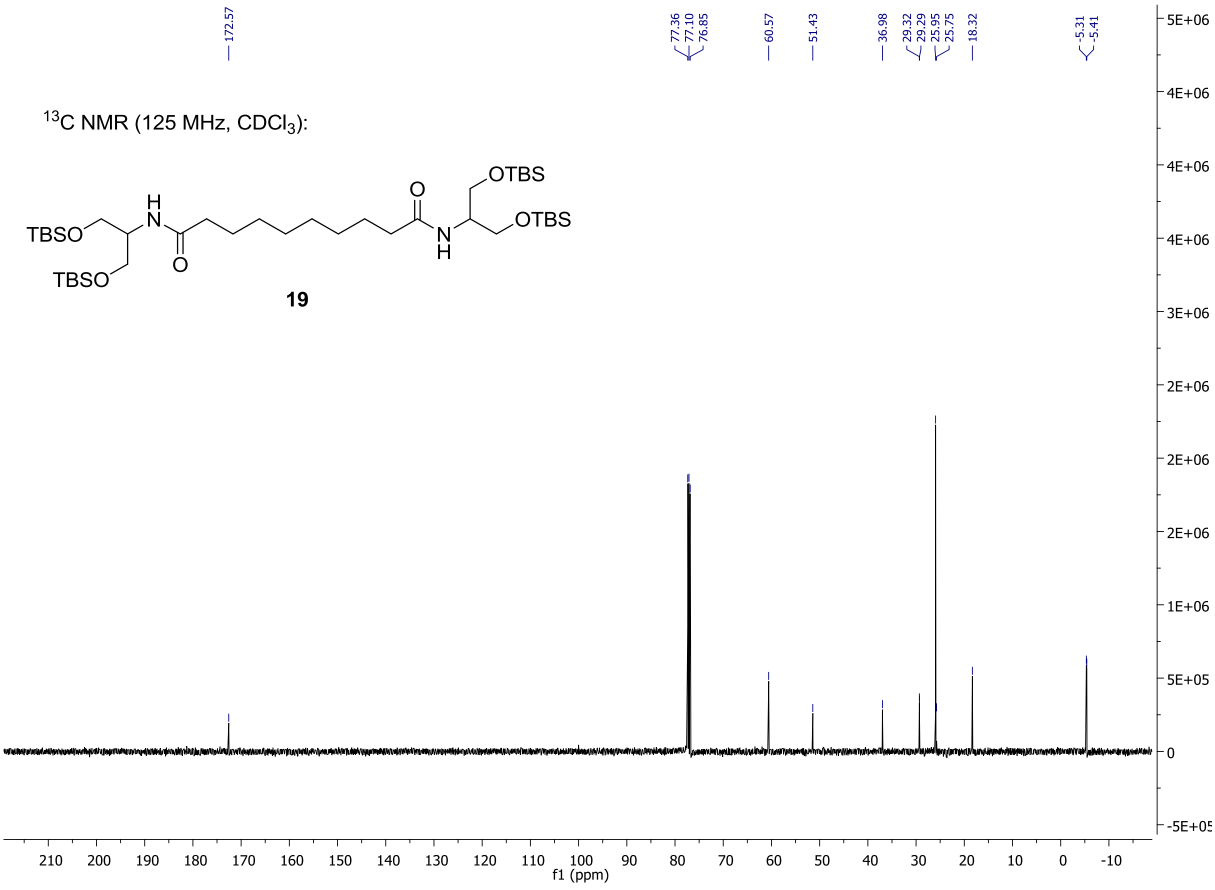

19

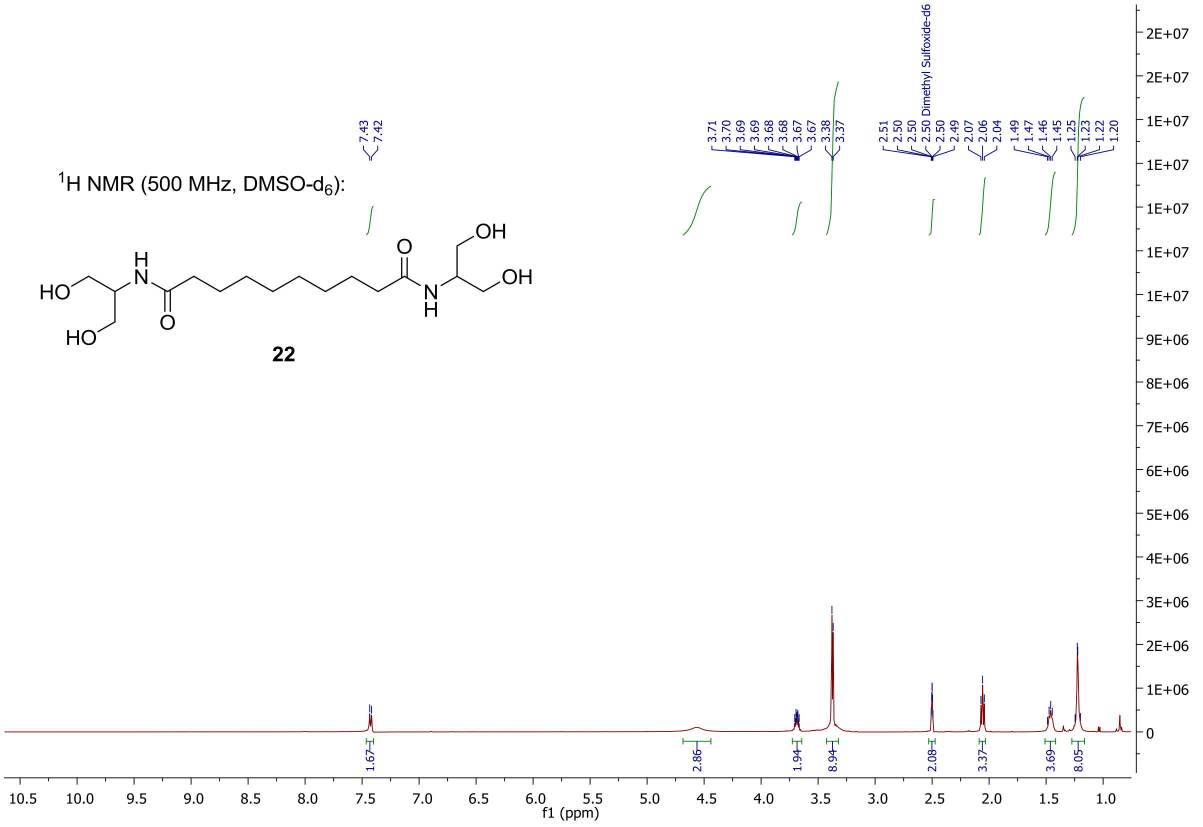

^{1}H NMR (500 MHz, DMSO-d$_6$):
22
7.43
7.42
3.71
3.70
3.69
3.69
3.68
3.68
3.67
3.67
3.38
3.37
2.51
2.50
2.50
2.50 Dimethyl Sulfoxide-d6
2.50
2.49
2.07
2.06
2.04
1.49
1.47
1.46
1.45
1.25
1.23
1.22
1.20
1.67
2.86
1.94
8.94
2.08
3.37
3.69
8.05
f1 (ppm)

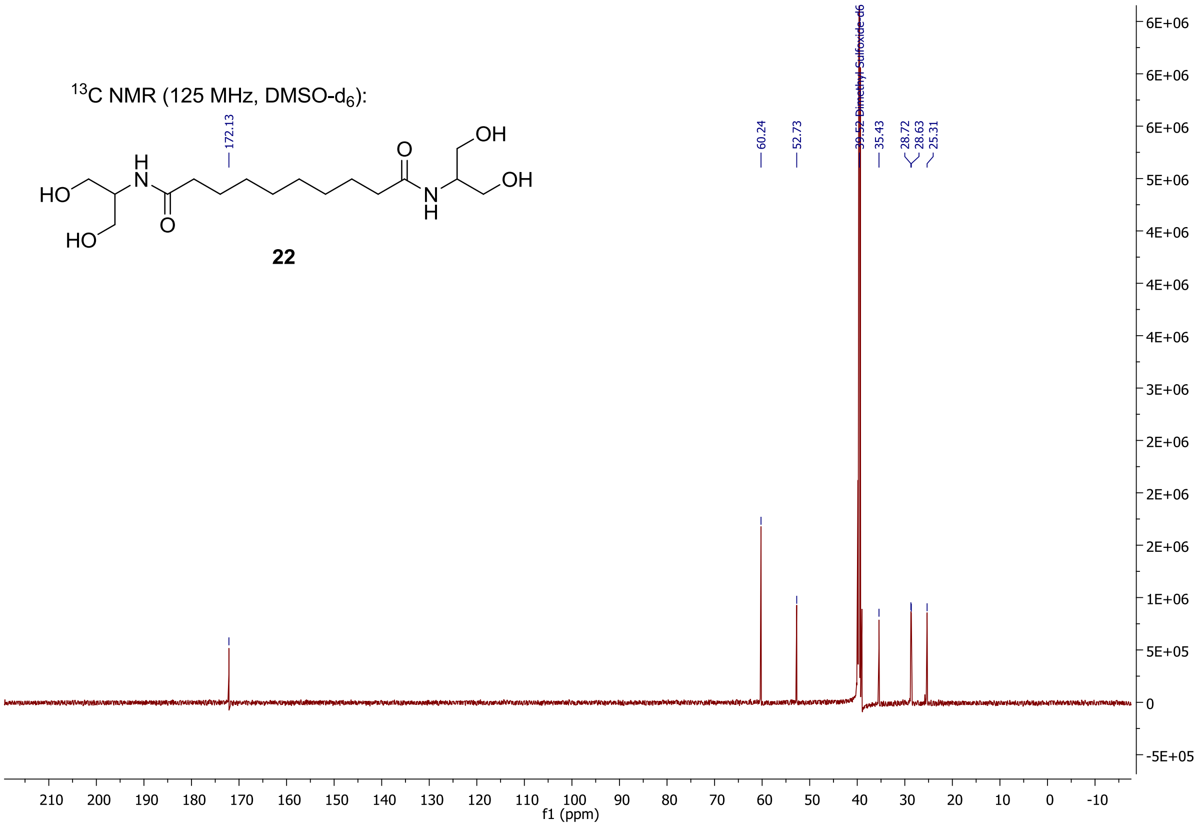

^{13}C NMR (125 MHz, DMSO-d$_6$):
22
172.13
60.24
52.73
39.52 Dimethyl Sulfoxide-d6
35.43
28.72
28.63
25.31
f1 (ppm)

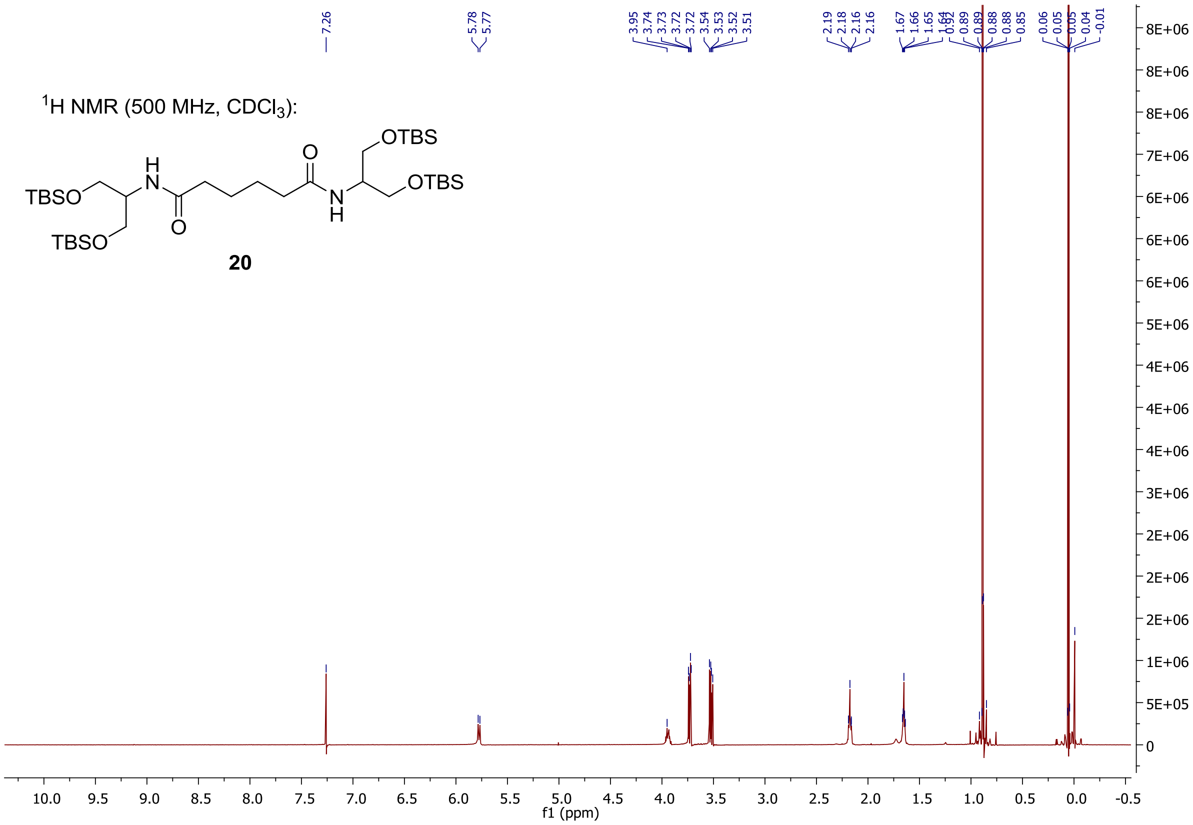
1H NMR (500 MHz, CDCl3):
TBSO
TBSO
OTBS
OTBS
20
7.26
5.78
5.77
3.95
3.74
3.73
3.72
3.72
3.54
3.53
3.52
3.51
2.19
2.18
2.16
2.16
1.67
1.66
1.65
0.89
0.88
0.88
0.85
0.06
0.05
0.04
-0.01
f1 (ppm)

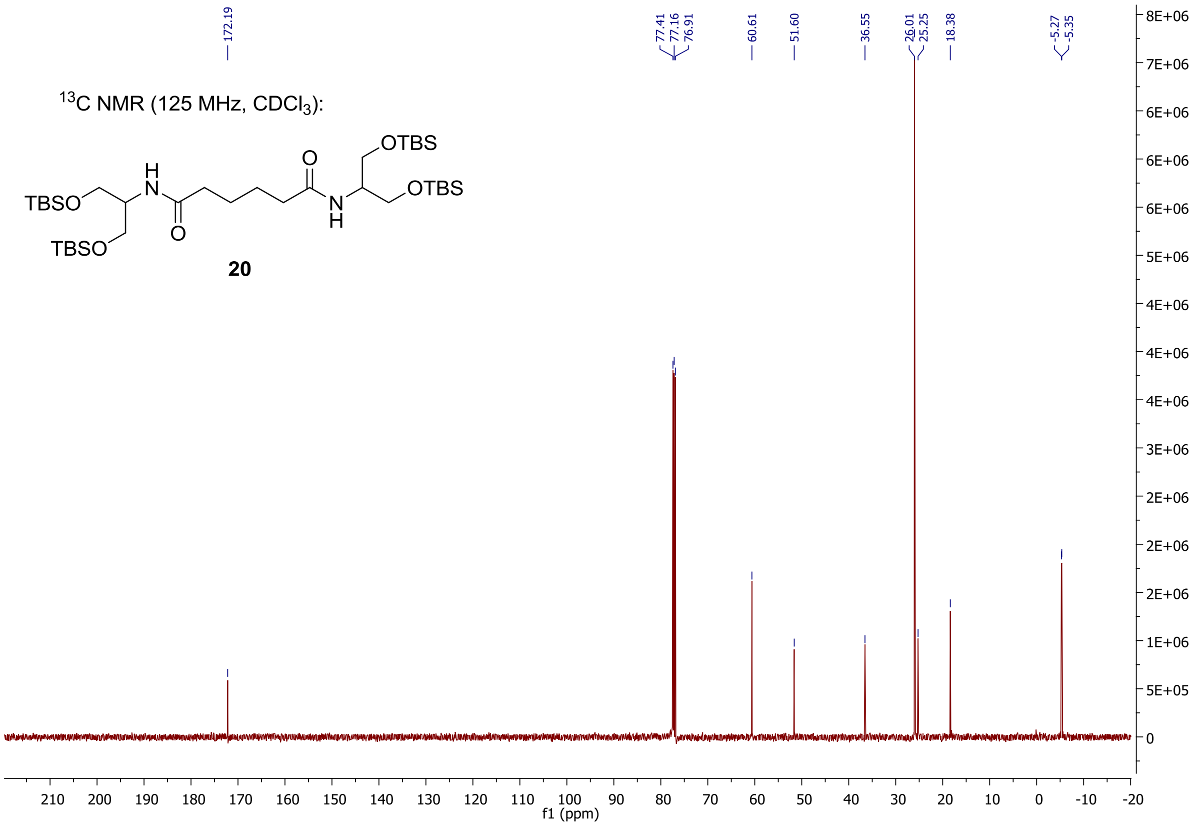
13C NMR (125 MHz, CDCl3):
TBSO
TBSO
OTBS
OTBS
20
172.19
77.41
77.16
76.91
60.61
51.60
36.55
26.01
25.25
18.38
-5.27
-5.35
f1 (ppm)

^{1}H NMR (500 MHz, DMSO-d_6):

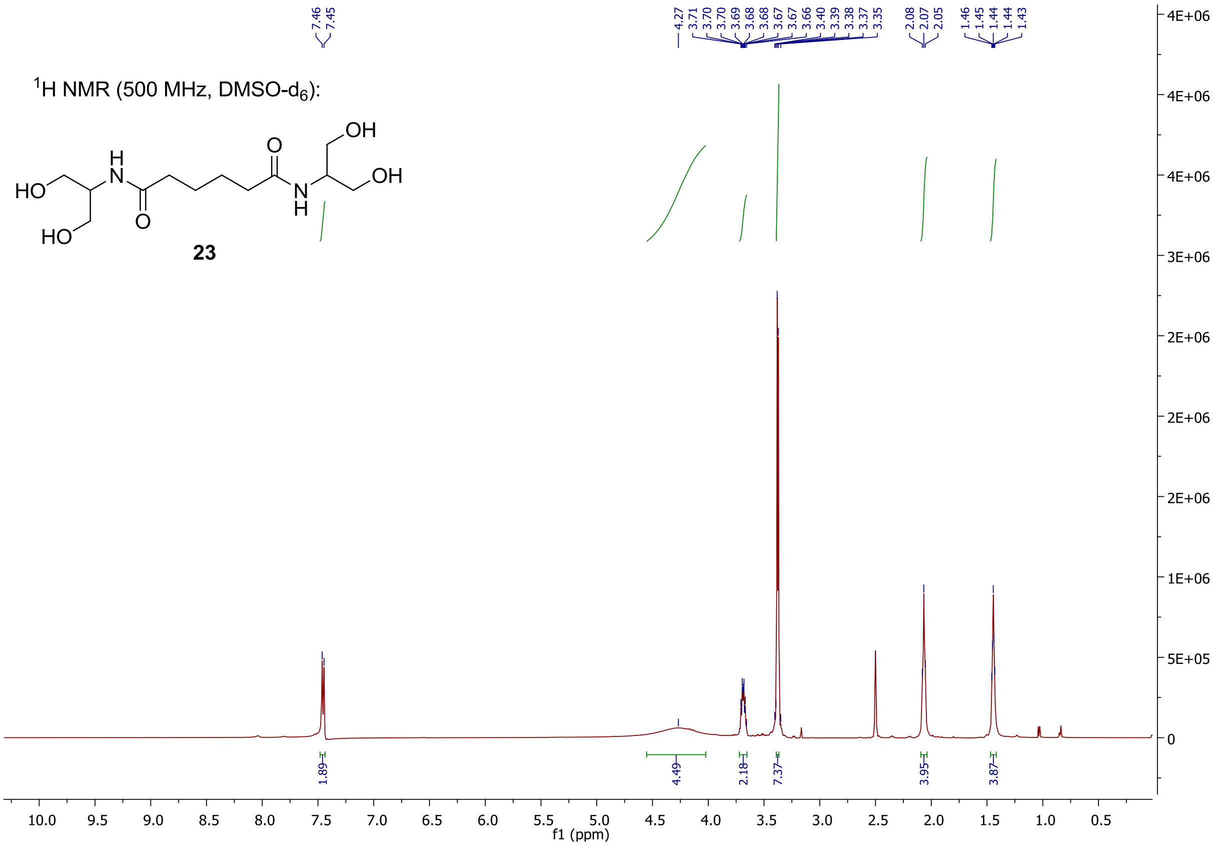

23

^{13}C NMR (125 MHz, DMSO-d_6):

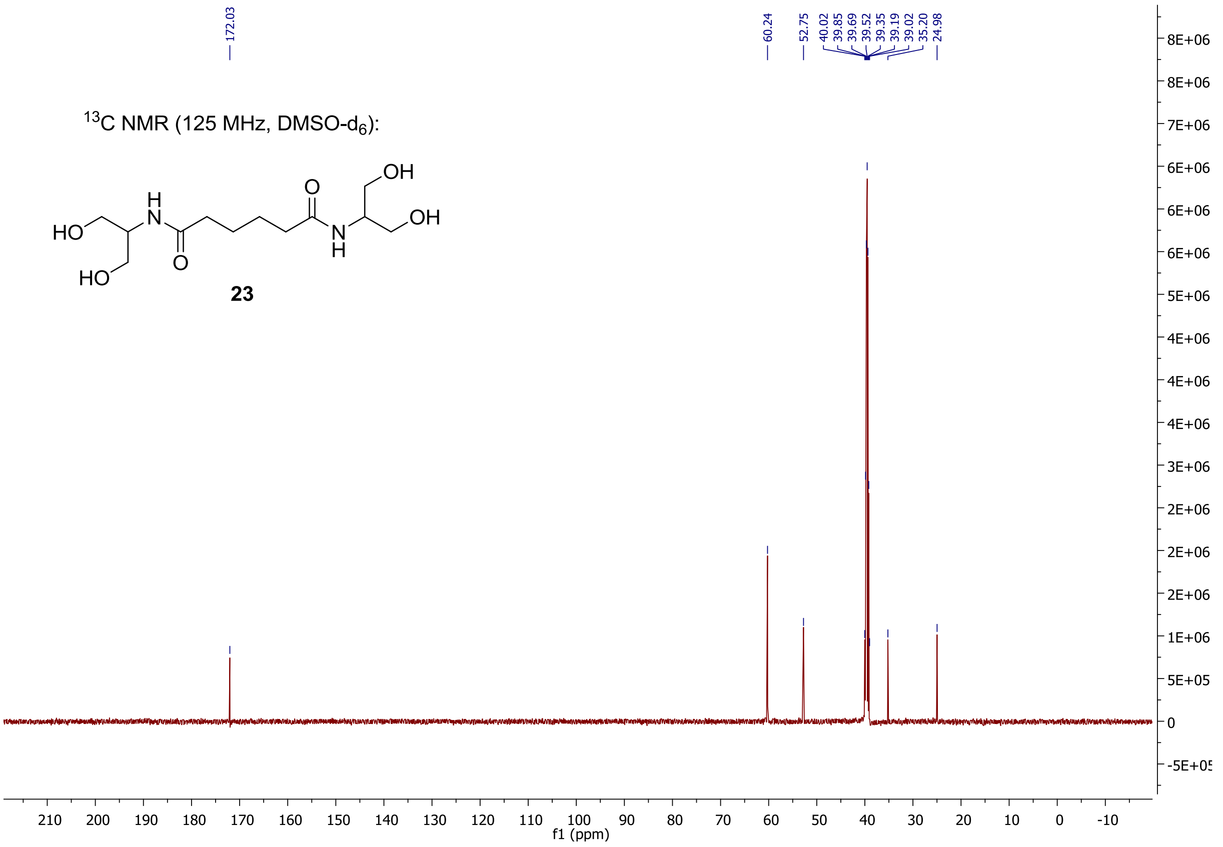

23

^{1}H NMR (500 MHz, CD_3OD):

25

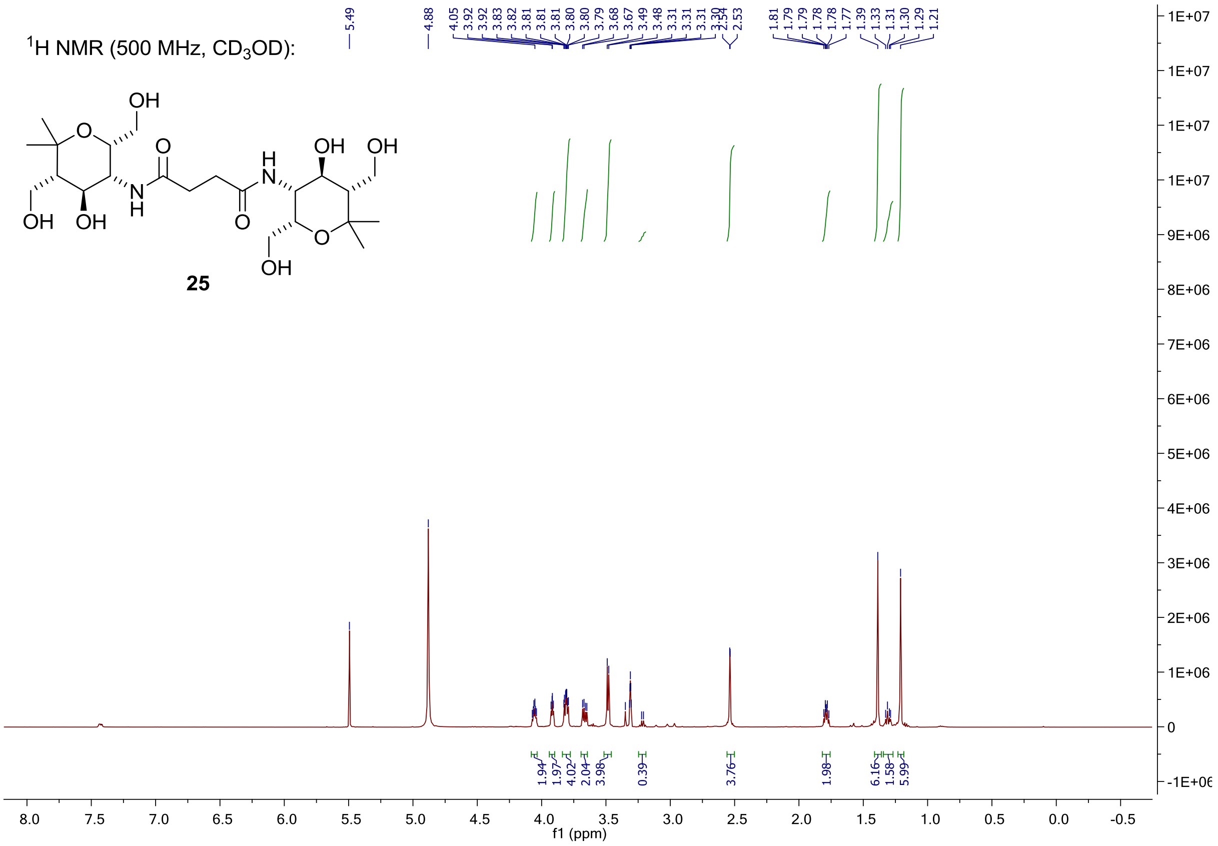

^{13}C NMR (125 MHz, CD_3OD):

25

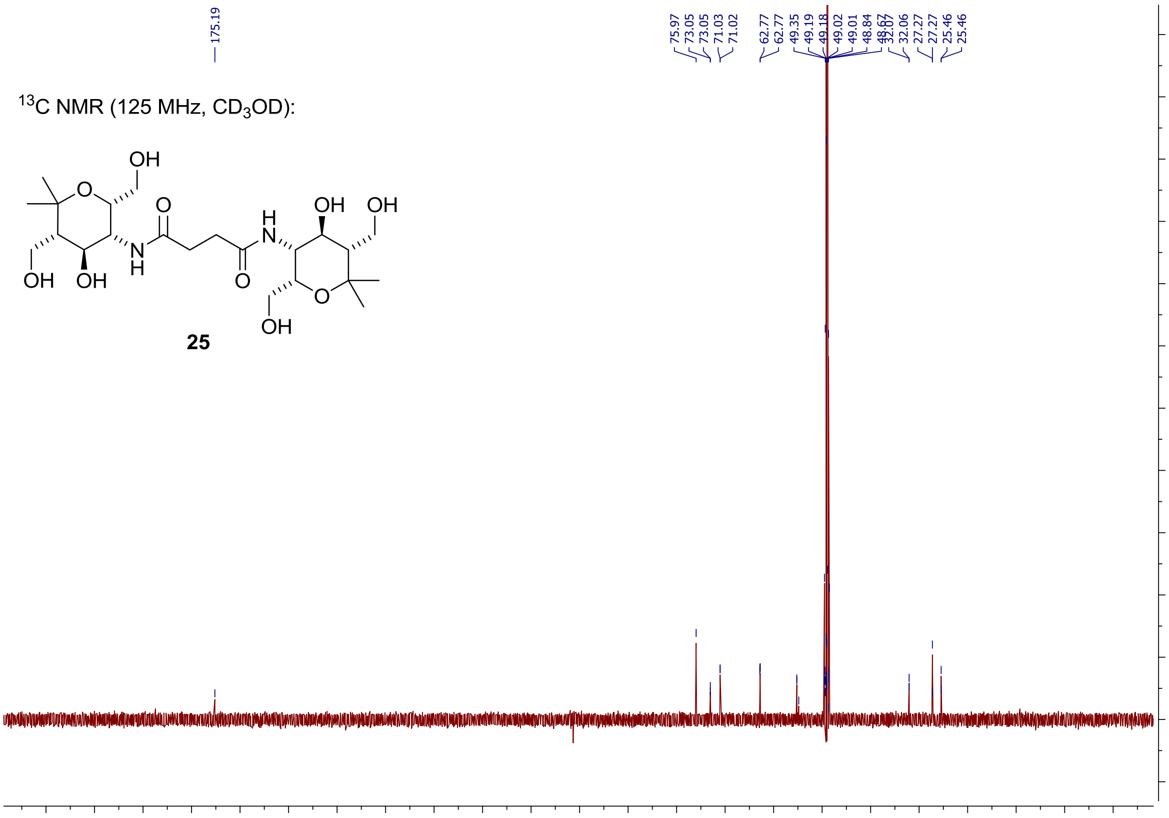

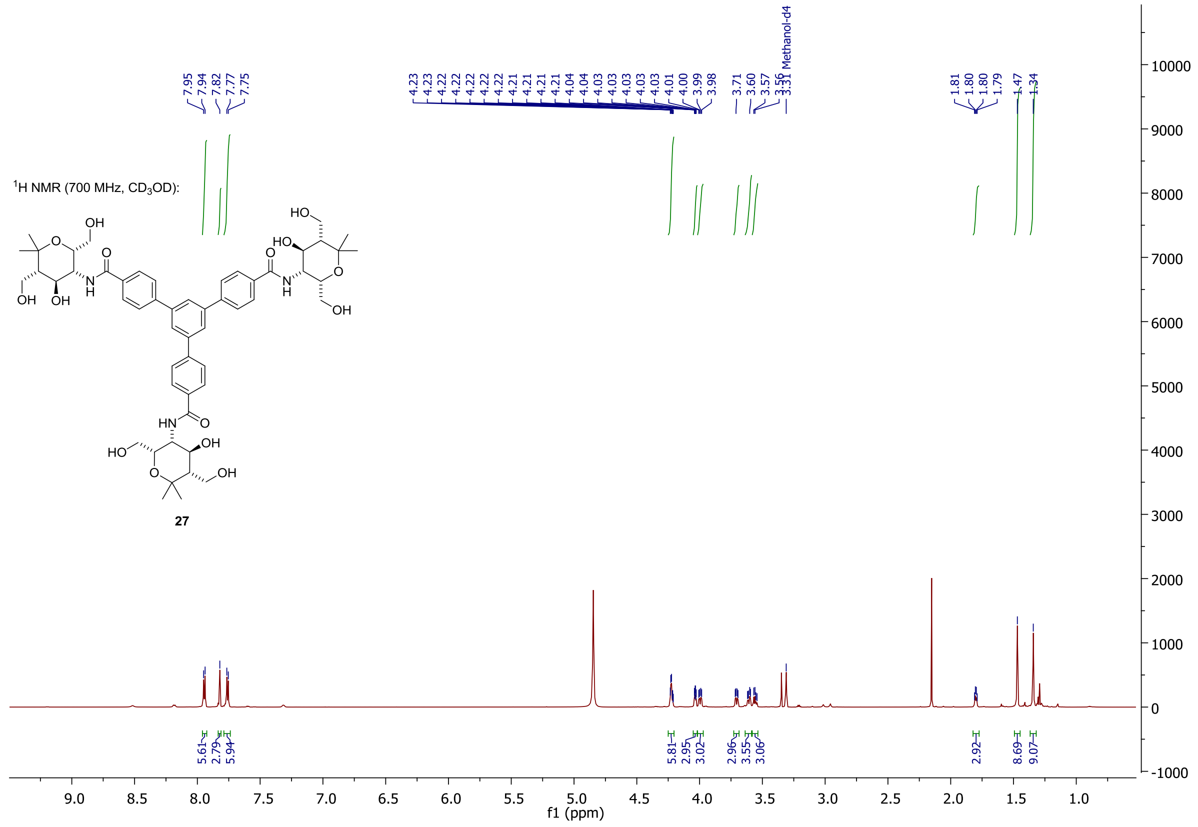

7.95 7.94 7.82 7.77 7.75
4.23 4.23 4.22 4.22 4.22 4.22 4.22 4.21 4.21 4.21 4.21 4.04 4.04 4.03 4.03 4.03 4.03 4.03 4.01 4.00 3.99 3.98
3.71 3.60 3.57 3.56 3.31 Methanol-d4
1.81 1.80 1.80 1.79 1.47 1.34
^{1}H NMR (700 MHz, CD_3OD):
27
5.61 2.79 5.94 5.81 2.95 3.02 2.96 3.55 3.06 2.92 8.69 9.07
f1 (ppm)

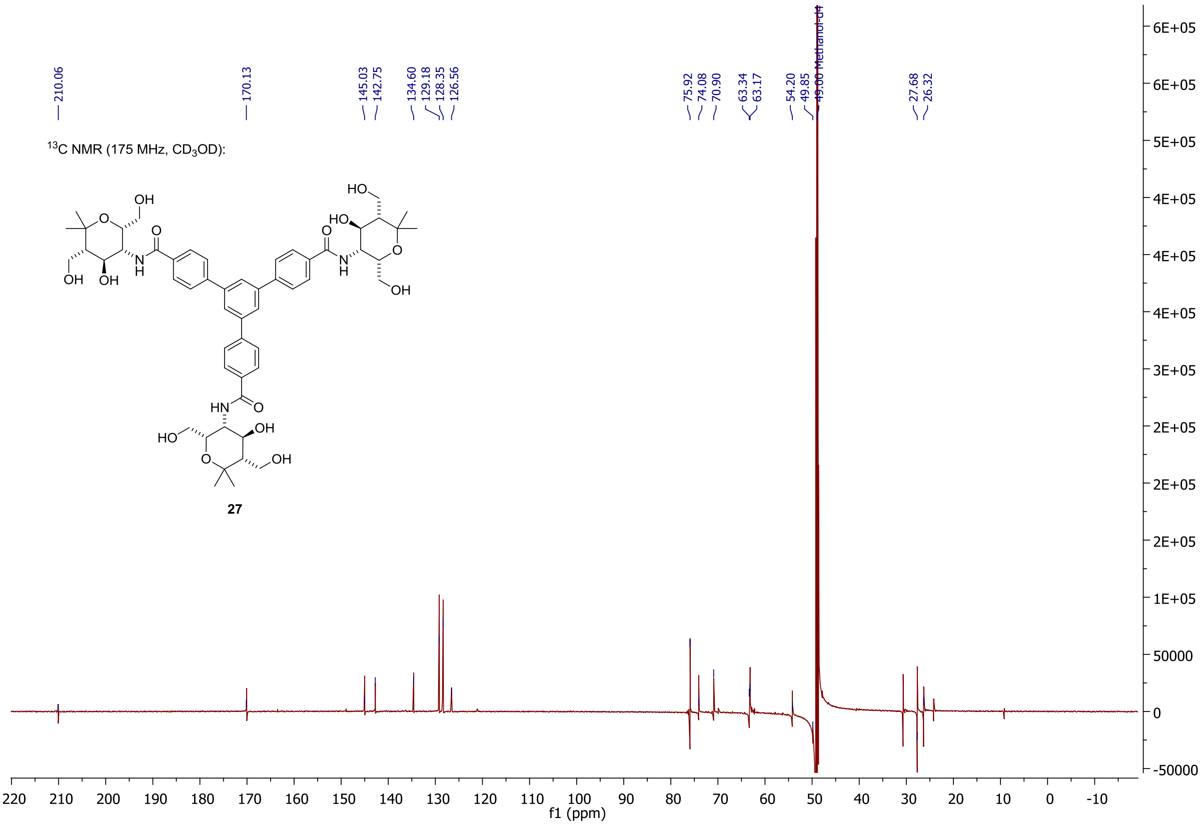

210.06
170.13
145.03 142.75
134.60 129.18 128.35 126.56
75.92 74.08 70.90
63.34 63.17
54.20 49.85 49.00 Methanol-d4
27.68 26.32
^{13}C NMR (175 MHz, CD_3OD):
27
f1 (ppm)

^{1}H NMR (700 MHz, $CDCl_3$):

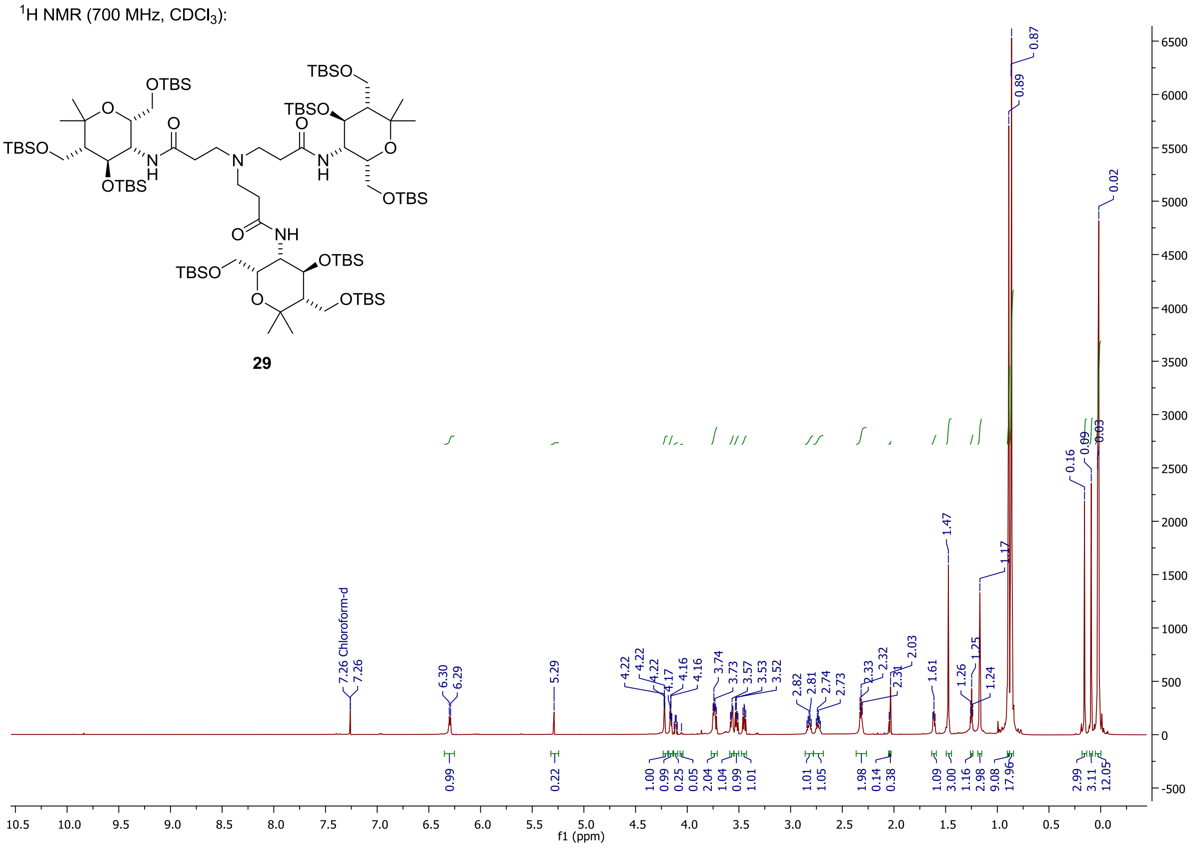

^{13}C NMR (175 MHz, $CDCl_3$):

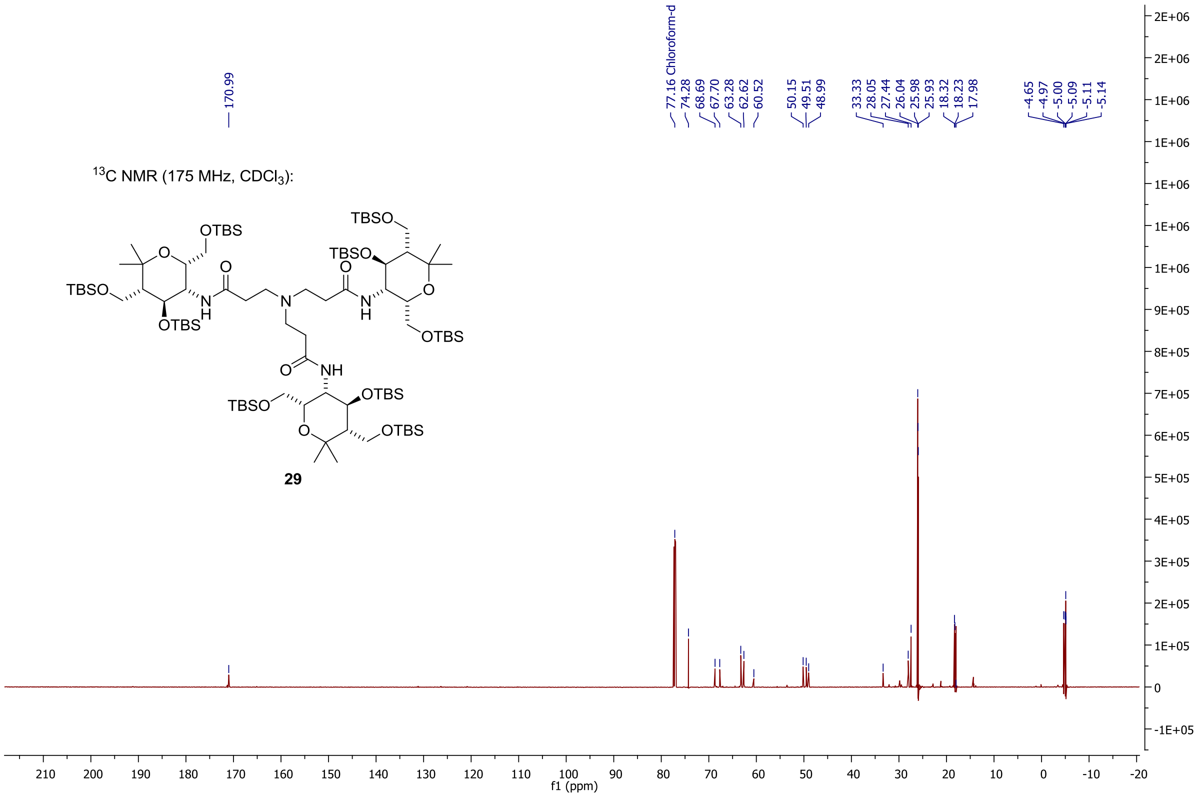

^{1}H NMR (700 MHz, D_2O):

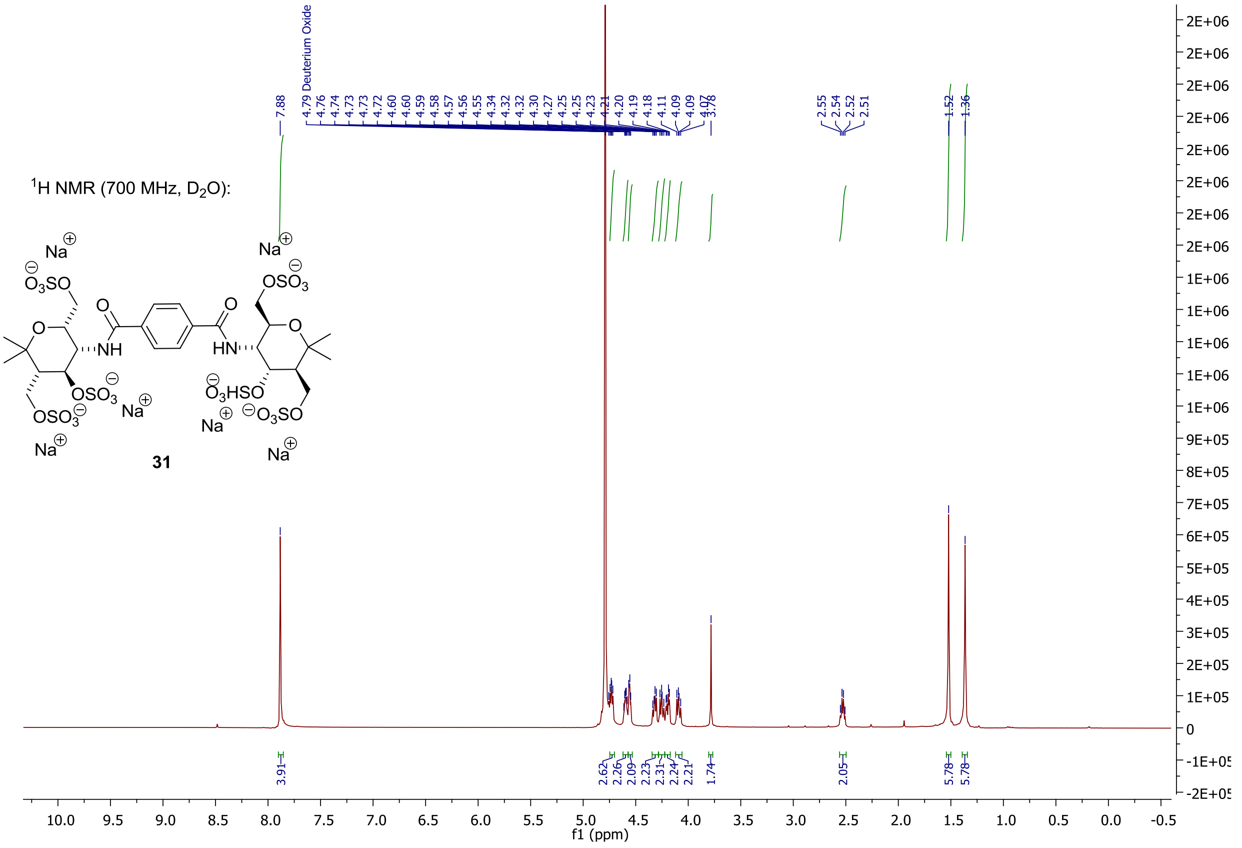

31

^{13}C NMR (175 MHz, D_2O):

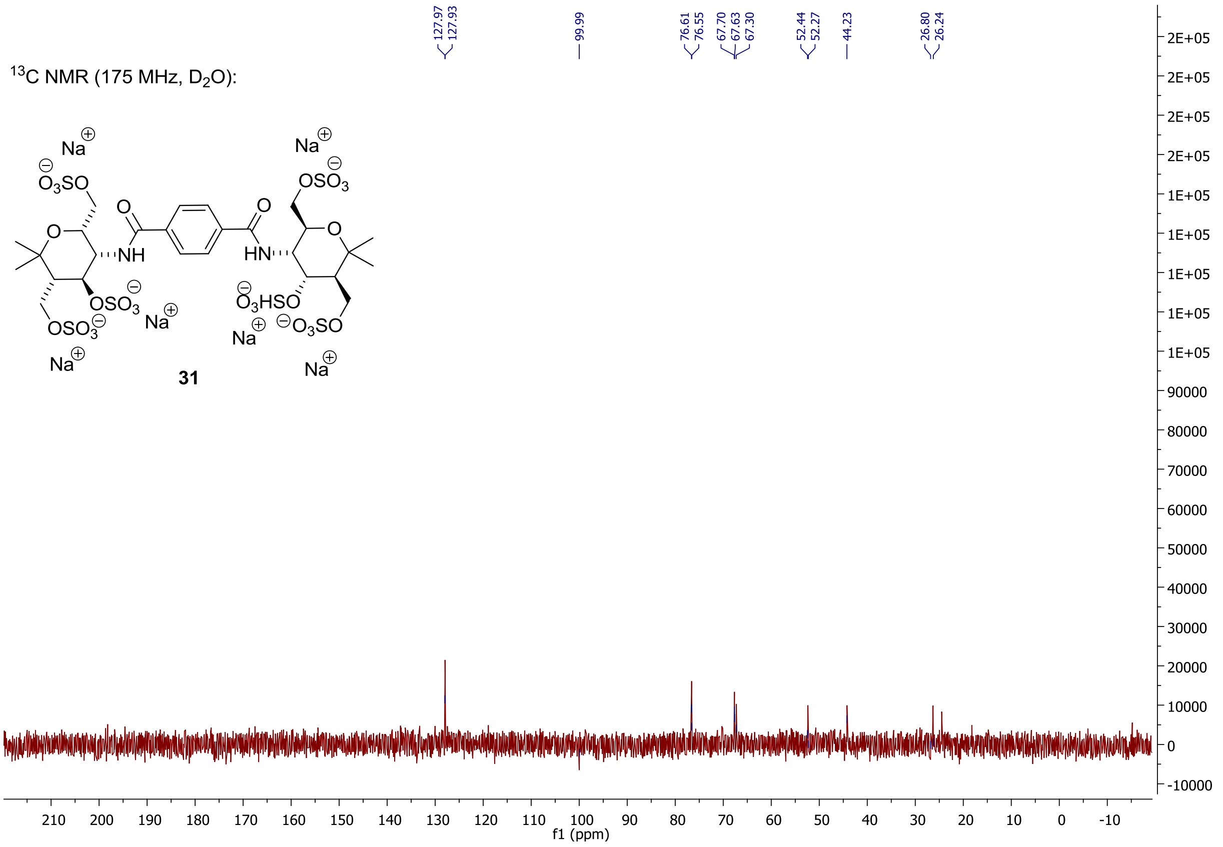

31

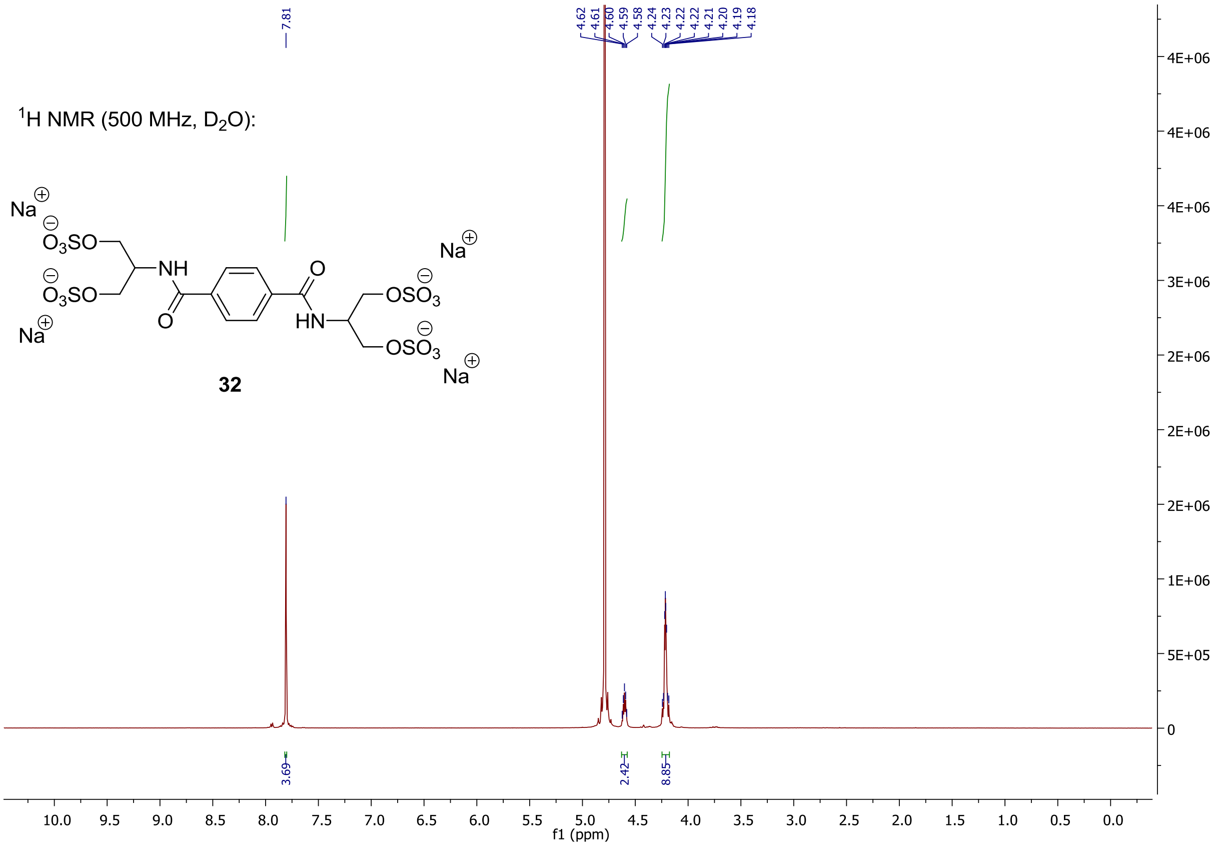

^{1}H NMR (500 MHz, D_2O):
32
7.81
4.62 4.61 4.60 4.59 4.58 4.24 4.23 4.22 4.22 4.21 4.20 4.19 4.18
3.69
2.42
8.85
f1 (ppm)

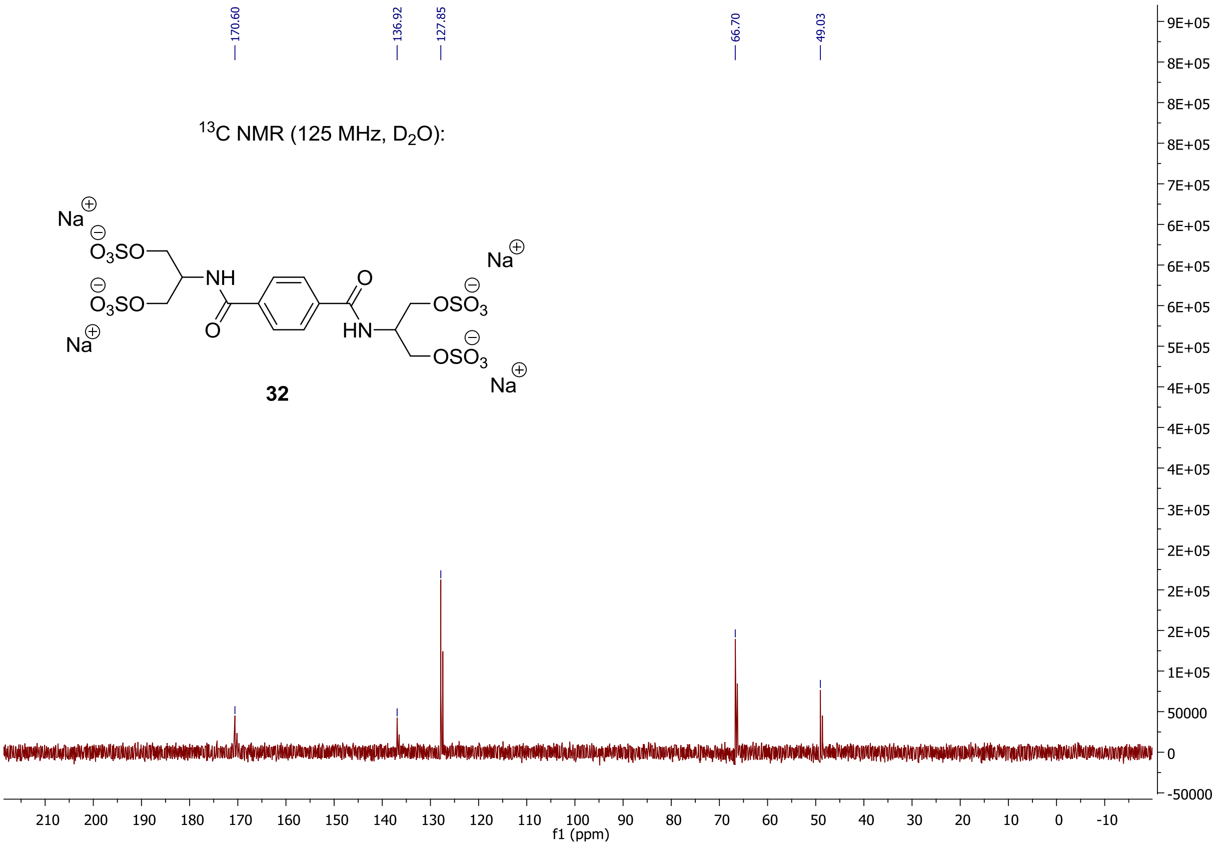

^{13}C NMR (125 MHz, D_2O):
32
170.60
136.92
127.85
66.70
49.03
f1 (ppm)

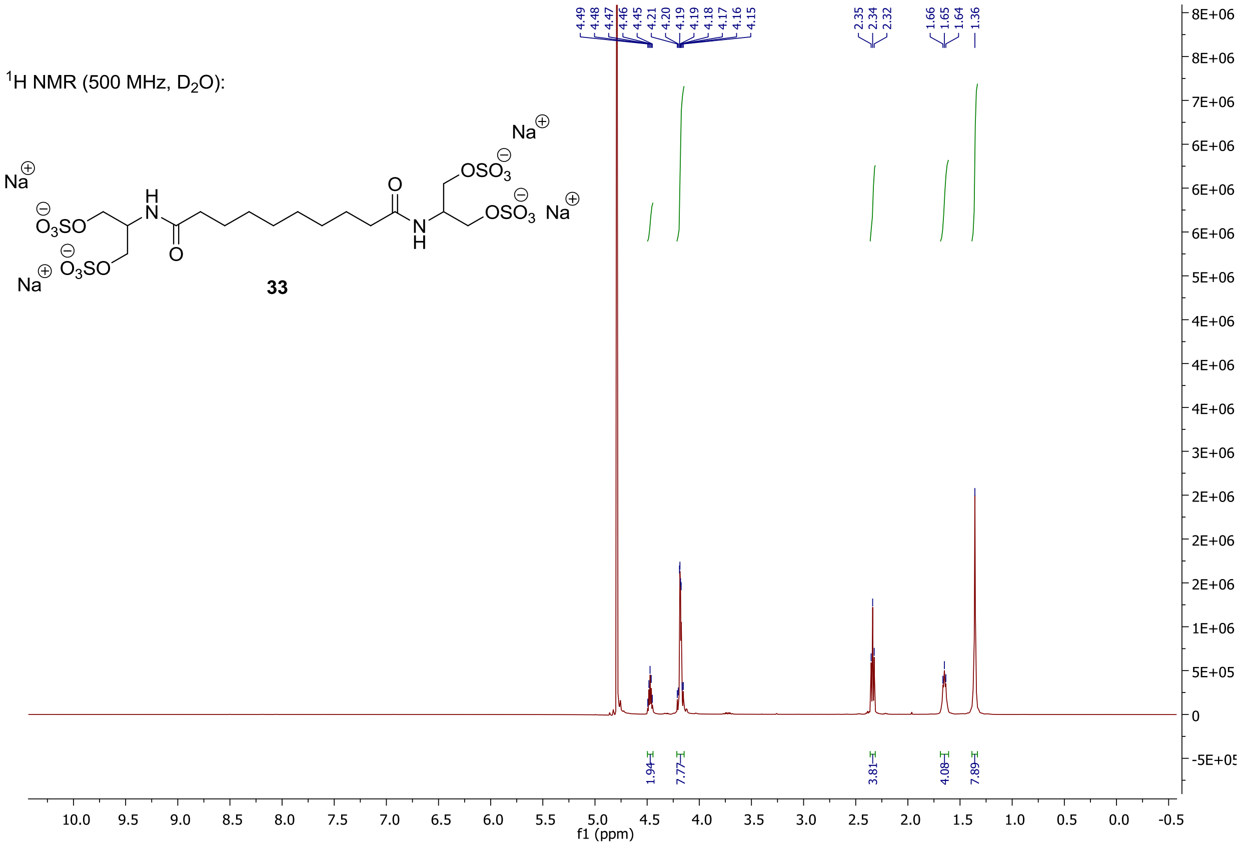

^{1}H NMR (500 MHz, D_2O):
33
4.49 4.48 4.47 4.46 4.45 4.21 4.20 4.19 4.19 4.18 4.17 4.16 4.15
2.35 2.34 2.32
1.66 1.65 1.64
1.36
1.94 7.77 3.81 4.08 7.89
f1 (ppm)

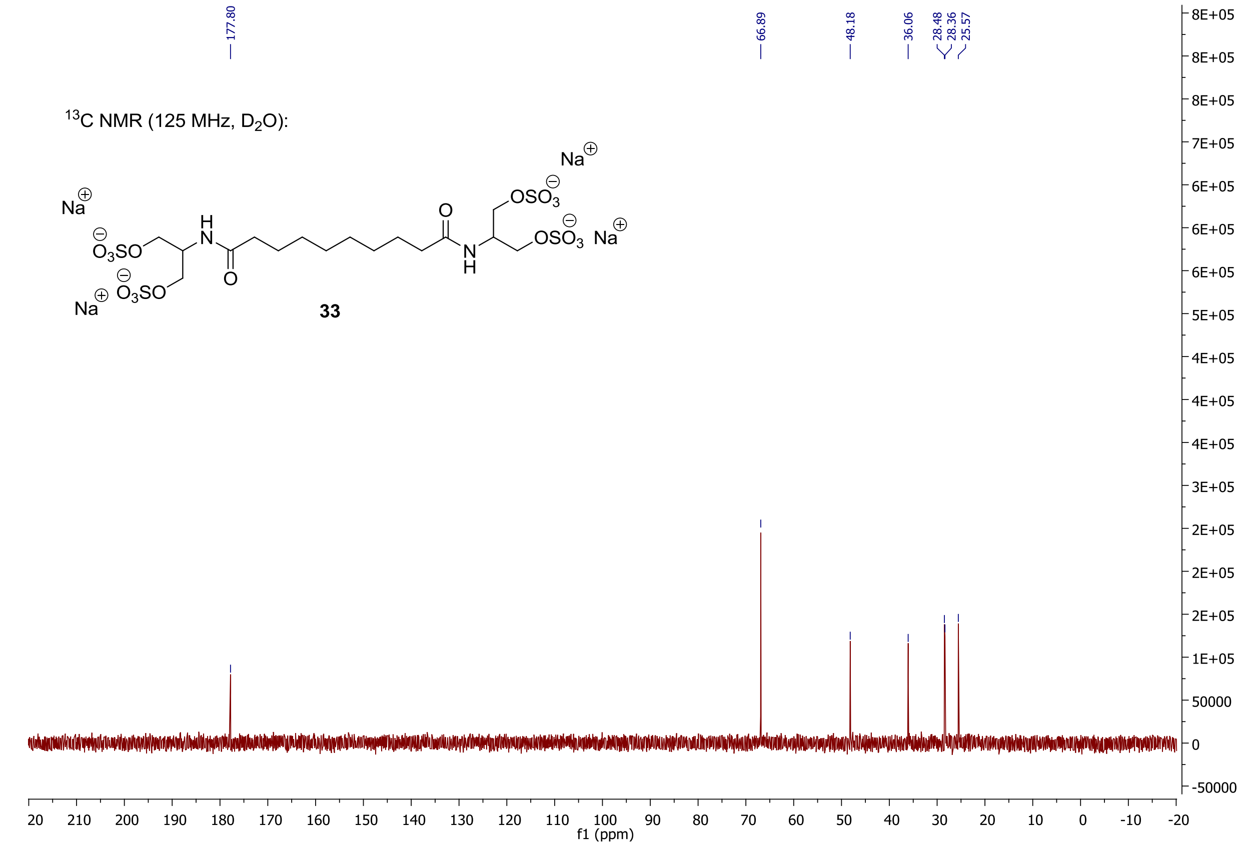

^{13}C NMR (125 MHz, D_2O):
33
177.80
66.89
48.18
36.06
28.48 28.36 25.57
f1 (ppm)

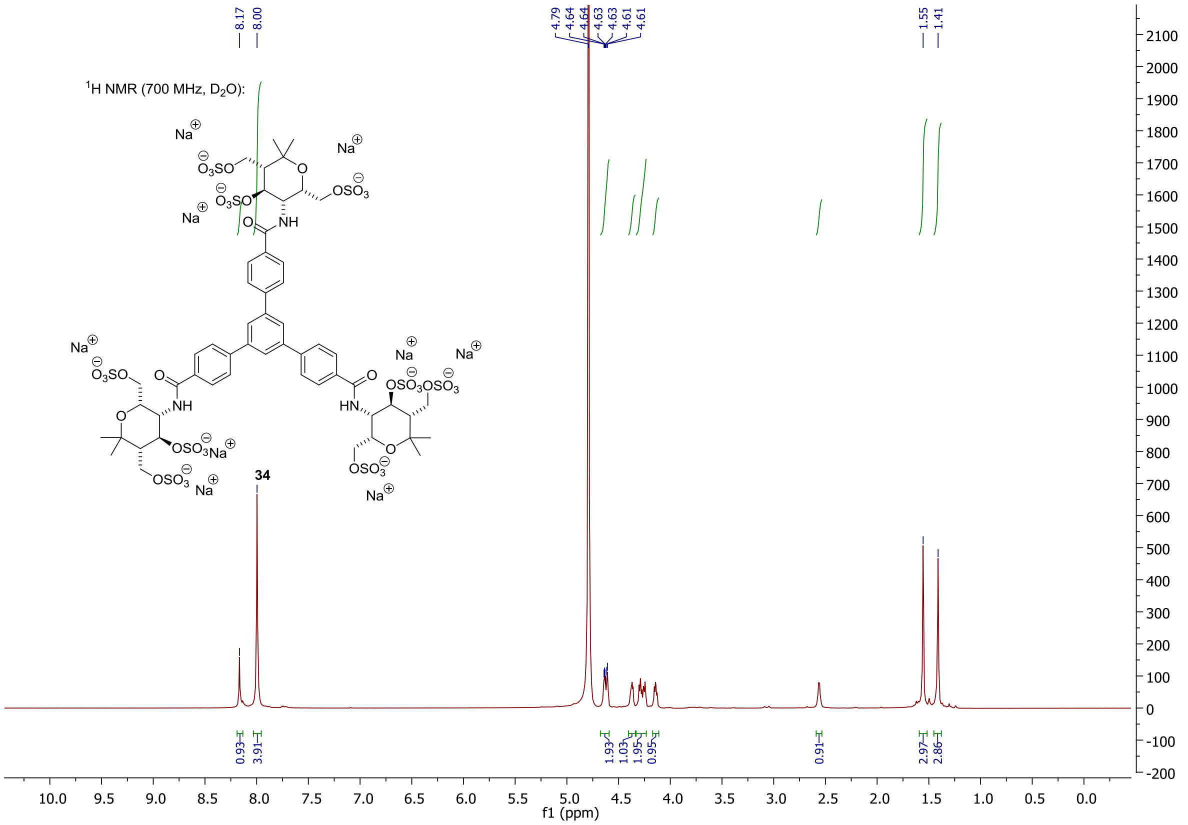

^1H NMR (700 MHz, D_2O):
34
8.17
8.00
4.79
4.64
4.64
4.63
4.63
4.61
4.61
1.55
1.41
0.93
3.91
1.93
1.03
1.95
0.95
0.91
2.97
2.86
f1 (ppm)

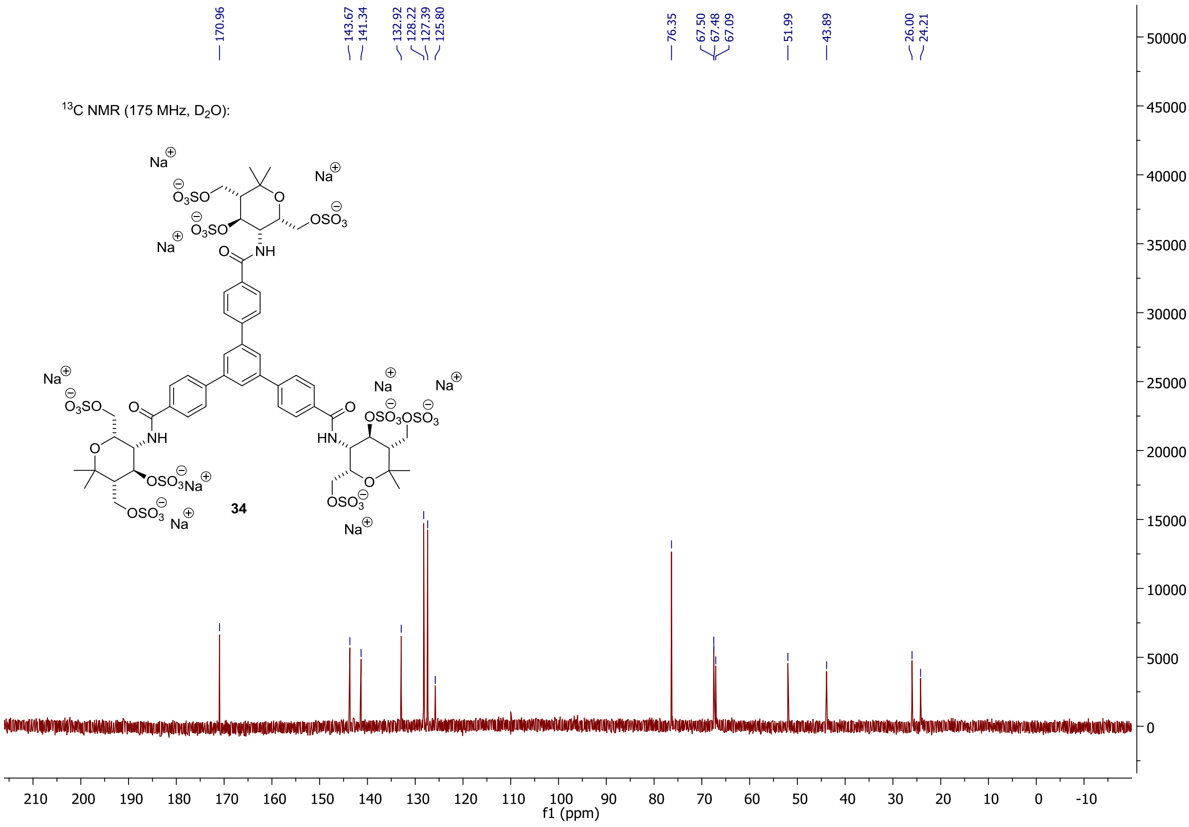

^{13}C NMR (175 MHz, D_2O):
34
170.96
143.67
141.34
132.92
128.22
127.39
125.80
76.35
67.50
67.48
67.09
51.99
43.89
26.00
24.21
f1 (ppm)

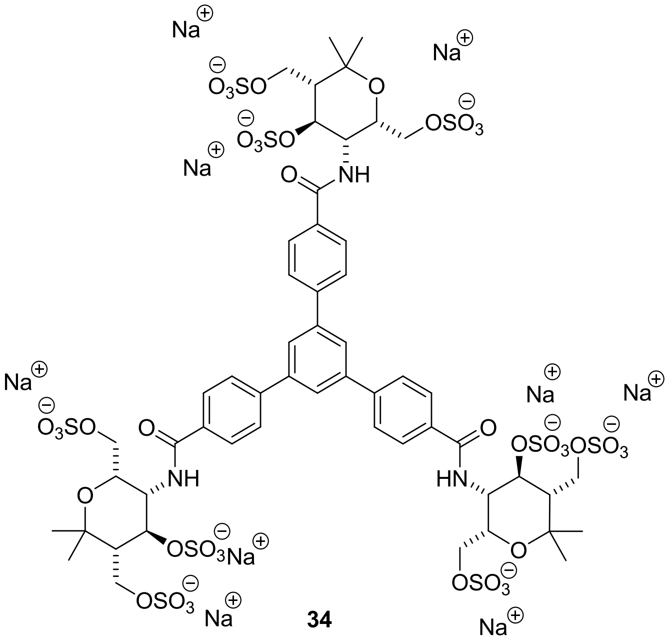

34

Baseline Corrected Values		Inhibitor Concentration [nM]	Error/ Diviation
100%			
2	-0,04	1000000	0,05
3	0,07	100000	0,00
4	0,09	10000	0,02
5	0,73	1000	0,06
6	0,92	100	0,00

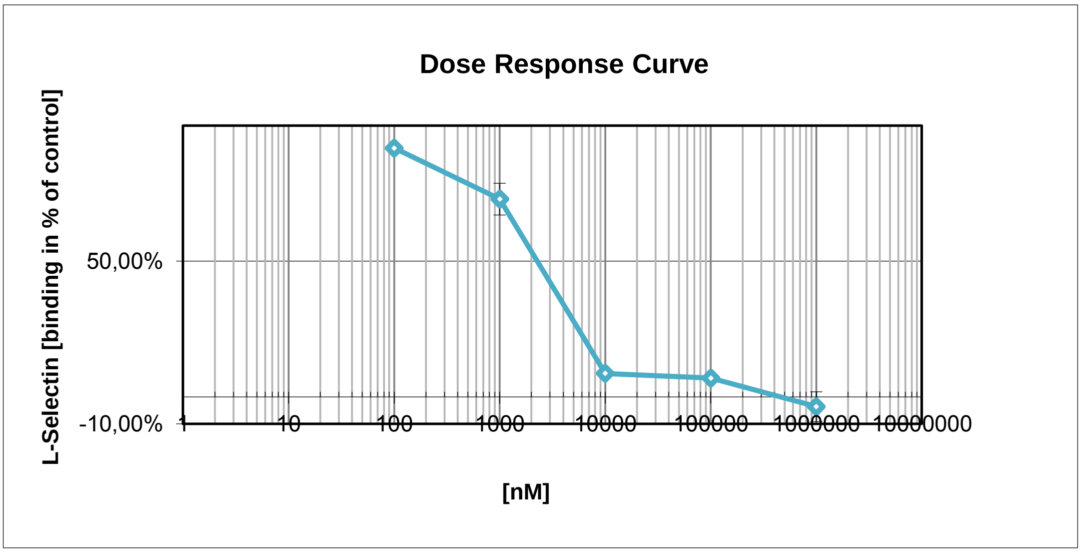

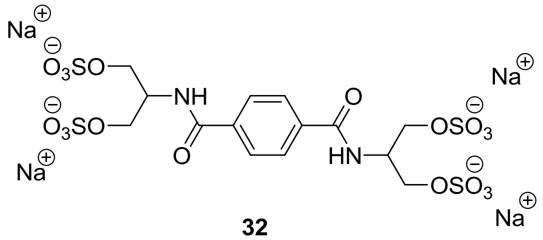

32

Baseline Corrected Values		Inhibitor Concentration [nM]	Error/ Diviation
100%			
2	0,01	30000	0,03
3	0,35	3000	0,02
4	0,65	300	0,01

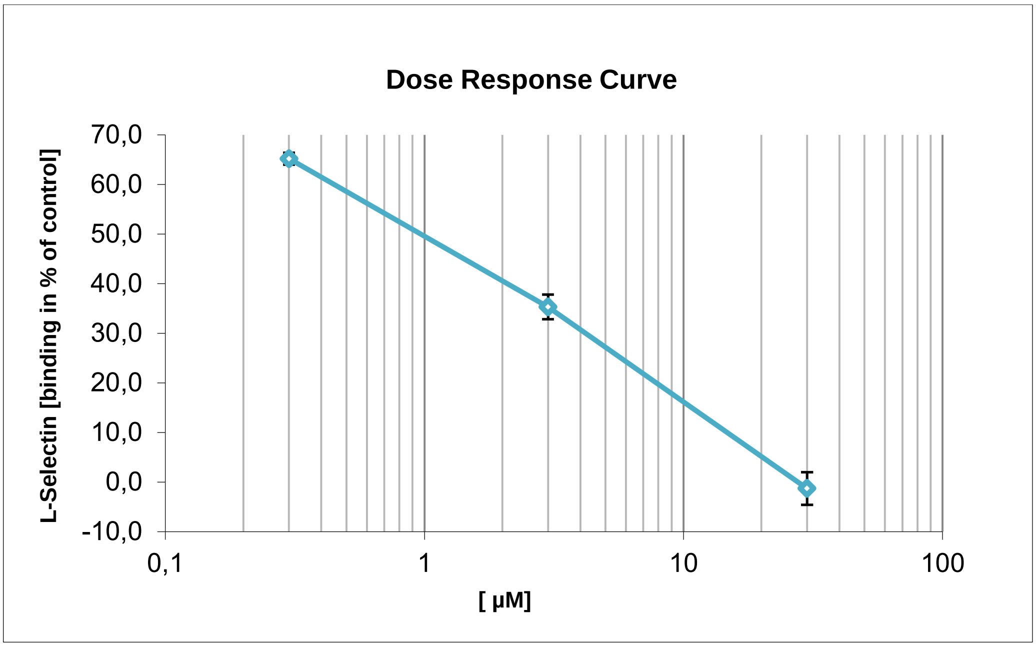

Supplement: File 1 — General information, experimental procedures and analytical data as well as copies of NMR spectra of all compounds. [file Beilstein_J_Org_Chem-11-638-s001.pdf]
